# Cost of cardiovascular diseases and renal complications in people with type 2 diabetes mellitus in the Kingdom of Saudi Arabia: A retrospective analysis of claims database

**DOI:** 10.1371/journal.pone.0273836

**Published:** 2022-10-20

**Authors:** Ahmed Hamden Al-Jedai, Hajer Yousef Almudaiheem, Dema Abdulrahman Alissa, Hadi Saeed Al-Enazy, Ghazwa B. Korayem, Ahlam Alghamdi, Shabab Alghamdi

**Affiliations:** 1 Therapeutic Affairs, Ministry of Health, Riyadh, Saudi Arabia; 2 Clinical Pharmacy, Colleges of Pharmacy and Medicine, Alfaisal University, Riyadh, Saudi Arabia; 3 Therapeutic Affairs Deputyship, Ministry of Health, Riyadh, Saudi Arabia; 4 Department of Family Medicine, Johns Hopkins Aramco Healthcare, Dhahran, Saudi Arabia; 5 Department of Emergency Medicine and Resuscitation, Johns Hopkins Aramco Healthcare, Dhahran, Saudi Arabia; 6 Department of Simulation and Medical Education, Johns Hopkins Aramco Healthcare, Dhahran, Saudi Arabia; 7 Wellness Institute, Johns Hopkins Aramco Healthcare, Dhahran, Saudi Arabia; 8 Central Board for Accreditation of Healthcare Institutions, Riyadh, Saudi Arabia; 9 Council of Health Insurance, Riyadh, Saudi Arabia; 10 Family Medicine Scientific Committee, The Saudi Commission for Health Specialities, Riyadh, Saudi Arabia; 11 Eastern Province Office, Saudi Society of Family Medicine, Riyadh, Saudi Arabia; 12 Department of Pharmacy Practice, College of Pharmacy, Princess Nourah Bint Abdulrahman University, Riyadh, Saudi Arabia; 13 Princess Nourah University, Riyadh, Saudi Arabia; 14 Pharmaceutical Care Services, King Abdullah Bin Abdulaziz University Hospital, Riyadh, Saudi Arabia; 15 Council of Cooperative Health Insurance, Riyadh, Saudi Arabia; Polytechnic Institute of Coimbra: Instituto Politecnico de Coimbra, PORTUGAL

## Abstract

**Background:**

The burden of macro- and microvascular complications in patients with Type 2 diabetes mellitus (T2DM) is substantial in Middle East countries. The current study assessed the healthcare resource utilization (HCRU) and costs related to cardiovascular and renal complications among patients with T2DM.

**Methodology:**

This non-interventional, longitudinal, retrospective, cohort study collected secondary data from three insurance claims databases across Kingdom of Saudi Arabia (KSA) of patients diagnosed with T2DM. The study included adult patients aged ≥18 years diagnosed with first cardiovascular disease (CVD) during index time period and at least one T2DM claim anytime during the study time period. The primary analyses were conducted per database, stratified by three cohorts; patients with at least one claim every six months during the 1-year pre-index and 1-year post-index period (cohort 1), patients with at least one claim every six months during the 1-year pre-index, and two years post-index period (cohort 2) and patients with at least one claim every six months during the 1-year pre-index and 3-year post-index period (cohort 3). For each Payer database, demographics, CVD subgroups, HCRU, and costs were analysed. Descriptive statistics were used to analyse the data.

**Results:**

The study sample comprised of 72–78% male and 22–28% female T2DM patients with CVD and renal complications. Patients in the age group of 35–65 years or above contributed to the significant disease burden. Nearly 68 to 80% of T2DM patients developed one CVD event, and 19 to 31% of patients developed multiple CVD events during the follow-up period. For most patients with comorbid CVD and renal disease, the average HCRU cost for post‑index periods was higher compared to 1-year pre-index period across the different visit types and activities.

**Conclusion:**

The study findings elucidates the need for early initiation of therapies that would reduce the long-term cardiovascular and renal outcomes and the associated costs in patients with T2DM.

## 1. Introduction

Diabetes mellitus and its complications constitute a major public health concern, contributing to substantial morbidity and mortality in both developed and developing countries [1].In 2019, it was estimated that 9.3% (463 million) of the population worldwide suffered from the disease, and the numbers are expected to rise to 10.2% (578 million) by 2030 and 10.9% (700 million) by 2045 [1]. This global diabetes explosion is likely to be fueled by an aging population, economic development, and increasing urbanization leading to more sedentary lifestyles and greater consumption of unhealthy foods linked with obesity [2].Type 2 diabetes mellitus (T2DM) accounts for 90% of all cases of diabetes [3]. In the Middle East and North Africa region, it is estimated that approximately 39 million people have diabetes, and this is expected to rise by 72% reaching 67 million in 2045 [4].According to the World Health Organization (WHO), the Kingdom of Saudi Arabia (KSA) has ranked as the country with second-highest prevalence of diabetes in the Middle East and the seventh globally, with diabetes affecting nearly 7 million individuals [5].

Cardiovascular diseases (CVDs) constitute a major group of comorbidities in patients with T2DM [6].The risk of mortality due to CVD is 2–4 fold higher in patients with T2DM than non-T2DM patients [7]. CVDs also lead to significant disability, reduced quality of life, and increased treatment costs for patients with T2DM [6, 8]. In recent years, focus of diabetic pharmacotherapy has been largely on establishing CV safety and CV benefit of all antidiabetic agents [9].Furthermore, the recently published American Diabetes Association recommend using certain glucose-lowering medications with proven CV benefits in patients with T2DM and atherosclerotic cardiovascular disease (ASCVD) or high ASCVD risk [10].

Evidence indicates that CVD has a substantial impact on direct medical costs both at patient and population level [6, 8]. The KSA reported the highest diabetes-related expenditure among the Gulf Cooperation Council (GCC) countries as it spends 21% of its total health expenditure on diabetes care compared to 16% and 19% in other GCC [7]. In 2013, the direct and indirect costs associated with CVD and diabetes reached almost $11 billion in GCC. This cost is estimated to increase to $67.9 billion by 2022, which is equal to one and a half times the healthcare budget of the GCC countries [11].

Chronic kidney disease is another frequent comorbid condition in T2DM patients and is associated with detrimental clinical outcomes and economic effects. Chronic kidney disease in T2DM patients contributes for increased healthcare resource utilization and costs to patients and healthcare system [12]. In a retrospective cohort study, incremental adjusted costs that occurred over follow-up (from baseline) was substantially higher among patients who progressed from baseline stage 0 to stage 4 CKD compared to those who did not progress. Across all stages of CKD, those who progressed to a higher stage of CKD from baseline had follow-up costs that ranged from 2 to 4 times higher than those who did not progress [13].

Healthcare services in KSA are currently provided free of charge to all Saudi citizens and expatriates working in the public sector, primarily through the Ministry of Health (MOH) [14].The MOH is the largest single Payer in the KSA healthcare system, financing over 60% of the services provided. While for others,healthcare insurance paid by their employers covers Saudi citizens and expatriates working in the private sector [15].

Complementary economic data regarding the cost of CVD and renal complications are critical for payers in understanding the financial implications of the change in prescribing recommendations for improving clinical outcomes in patients with T2DM. However, there is paucity in data pertaining to incremental CVD and renal cost in patients with T2DM. The present study aims to assess the initial and follow-up healthcare resource utilization (HCRU), and costs associated with CVD and renal complications, including breakdown per disease type, and compare with the cost for the preceding year among the patients with T2DM.

## 2. Methods

### 2.1 Study design and setting

**Fig 1:**
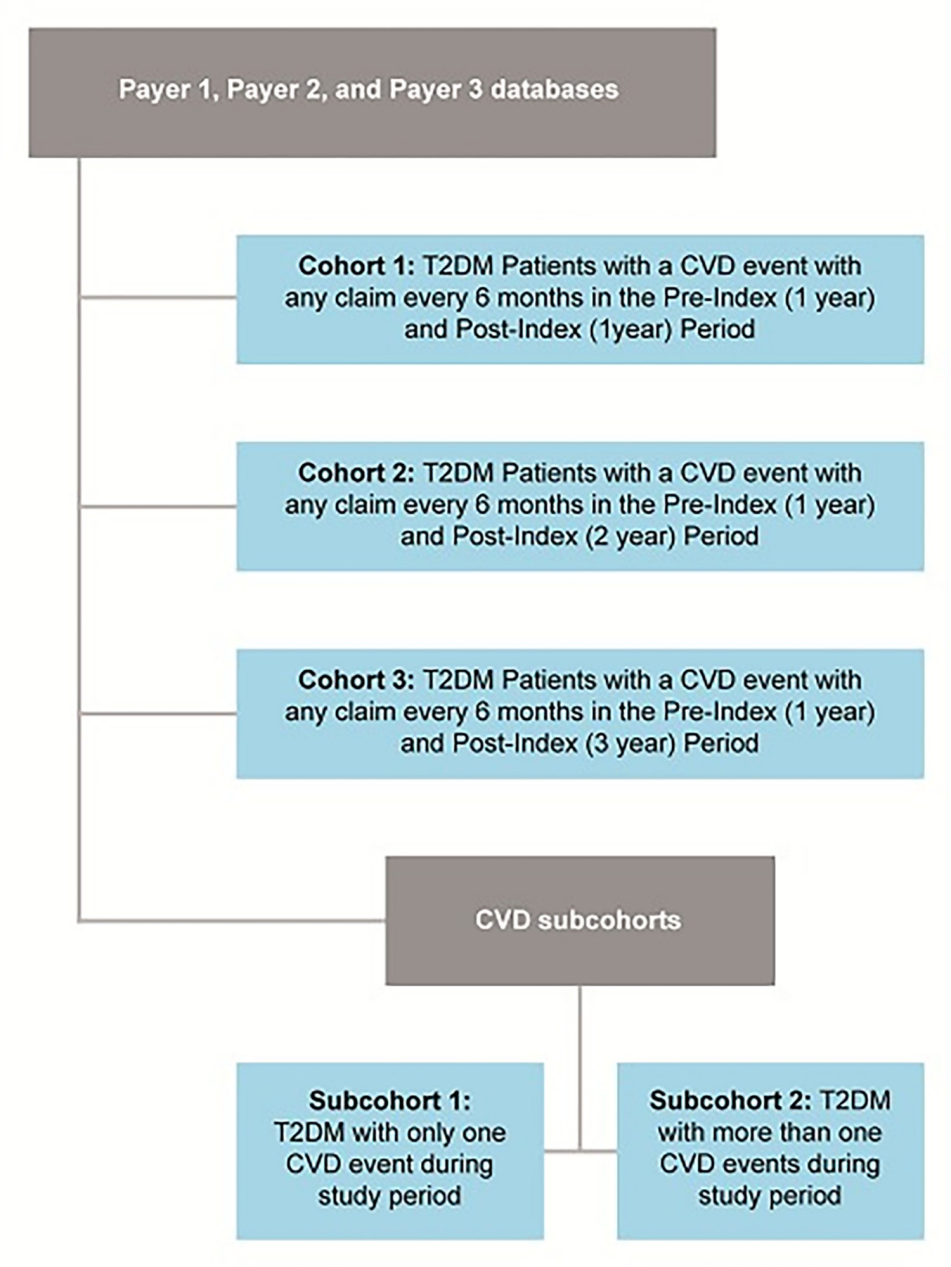
Patient stratification into cohorts and subcohorts based on follow-up duration and number of CVD events.

This was a non-interventional, longitudinal, retrospective, cohort study based on secondary data from 3 insurance claims databases (defined as “Payer 1”, “Payer 2”, and “Payer 3” databases), which recorded data across KSA of patients diagnosed with T2DM. The index date for each patient was defined as the date on which the first diagnosis of CVD was identified in patients with T2DM during the study time period (between 01 January 2016 through 31 December 2019 for Payer 1; 1 January 2015 to 31 December 2018 for Payer 2; and 01 January 2015 to 31 December 2019 for Payer 3). Patients in each database were further stratified into cohorts and subcohorts, as explained in Fig 1. Insurance claims data in KSA cover both the native Saudi population and expatriate population. Since this was an observational study, there were no treatments involved in this study.International Classification of Disease, Version 10 Clinical Modification (ICD‑10‑CM) codes was used to identify diagnoses.

### 2.2 Study population

Patients aged 18 years and above with first CVD diagnosis (index date) in the index time period, and who had at least one T2DM claim anytime during the study time period (defined in Study Design and Setting) were included in the analysis. Patients with any records of paediatric consultations, prescriptions, procedures, or services were not considered eligible for the study. Any patient diagnosed with CVD complication before a diagnosis of T2DM or diagnosed with type 1 diabetes mellitus or gestational diabetes during the study period was excluded from the analysis (Table 1).

**Table 1:** Patient disposition.

	Payer 1	Payer 2	Payer 3
	Number of patients (%)	Number of patients (%)	Number of patients (%)
Patient with first CVD indication on the Index date	58,493	19,636	22,964
Patients with at least one T2DM diagnosis in the entire study time period	29,473 (50)	7,035 (36)	22,798 (99)
Patients with CVD indication after T2DM	22,042 (75)	5,052 (72)	14,866 (65)
Patients with Age ≥18 years on the index date and excluding patient with pediatrics claims	11,215 (51)	5,052 (100)	14,477 (98)
Patients without T1DM and gestational diabetes in the entire study time period	9,419 (84)	3,748 (74)	10,910 (75)

CVD:Cardiovascular disease; T2DM:Type 2 diabetes mellitus;T1DM:Type 1 diabetes mellitus

### 2.3 Baseline variables and outcomes

For each payer databases, the following variables were analysed.

### 2.3.1 Demographics and CVD subgroups

Demographic characteristics such as age (available for Payer 1 and Payer 3), gender (available for Payer 1 and Payer 3), and nationality (available for Payer 1) was extracted from the one year pre-index period claim records.

### 2.3.2. HCRU and costs

Claims and costs (in Saudi Riyal) pertaining to inpatient and outpatient visits and activities including medications, procedures, consultations, services, and consumables were evaluated for post-index period (year 1,2 and 3), across all three databases. All-cause claims included all claims related to T2DM, CVDs, and other comorbid conditions in the patients. Disease-specific claims included claims specific to T2DM and CVD conditions only.

Each database captured overall data from hospitals, pharmacies, clinics, polyclinics, laboratories, and other providers. Databases also included longitudinal, anonymised, and patient-level data, representing a geographically varied range of administrative claims information.

### 2.3.3 Statistical analysis

This study used three analysis sets for Payer 1, Payer 2, and Payer 3. Analyses were conducted individually for each analysis set (data from all analysis sets were not combined). Descriptive statistics were used to summarise patient demographics (i.e., age, gender, and nationality), HCRU, and costs. Descriptive statistics using means for continuous variables and number of observations and percentages for categorical data. Data from each database were extracted and analysed by using SAS version 9.4 software.

### 2.3.4 Ethical consideration

Patient information and consent were not applicable since this study did not involve the interaction or interview with any patient, and the data did not include any individually identifiable variables. Data was anonymous for the purposes of this study and in compliance with applicable laws. Since the core study proposed herein did not involve collecting, using, or transmitting individually identifiable data, the Institutional Review Board’sBoard’s approval to conduct this study was not required.

The datasets were secured in an office that meets the requirements of the Health Insurance Portability and Accountability Act of 1996.

## 3. Results

### 3.1 Demographic characteristics

Overall, the data showed that approximately 50% of patients in the study were in 50–64 years age group; >20% of patients were in 35–49 years age group and approximately 20% of patients were in 65–79 years range. A very low proportion of patients (2–5%) were above 80 years of age. Majority of study population was represented by males (>70%). Approximately 25% of patients were native Saudi population and nearly 75% belonged to expatriate population (Table 2).

**Table 2:** Demographic characteristics of Payer 1 and Payer 3.

Category		T2DM with one CVD			T2DM with multiple CVD		
		Cohort 1	Cohort 2	Cohort 3	Cohort 1	Cohort 2	Cohort 3
Age, years (n) (%)	18–34	96 (3.6)	43 (4.1)	9 (4.1)	12 (1.7)	7 (2.3)	1 (1.3)
	35–49	685 (25.7)	283 (26.8)	48 (22.0)	149 (21.6)	70 (23.5)	20 (26.3)
	50–64	1433 (53.7)	560 (53.1)	126 (57.8)	370 (53.5)	157 (52.7)	37 (48.7)
	65–79	422 (15.8)	154 (14.6)	32 (14.7)	148 (21.4)	55 (18.5)	16 (21.1)
	80 and above	31 (1.2)	15 (1.4)	3 (1.4)	12 (1.7)	9 (3.0)	2 (2.6)
Gender (n) (%)	Female	621 (23.3)	246 (23.3)	61 (28.0)	106 (15.3)	50 (16.8)	21 (27.6)
	Male	2046 (76.7)	809 (76.7)	157 (72.0)	585 (84.7)	248 (83.2)	55 (72.4)
Nationality(n) (%)	Saudi	623 (23.4)	244 (23.1)	50 (22.9)	169 (24.5)	83 (27.9)	22 (28.9)
	Non-saudi	2044 (76.6)	811 (76.9)	168 (77.1)	522 (75.5)	215 (72.1)	54 (71.1)
Age, years (n) (%)	18–34	113 (4.2)	42 (4.6)	6 (3.4)	4 (0.7)	2 (0.8)	1 (1.8)
	35–49	661 (24.8)	224 (24.6)	53 (30.5)	122 (22.1)	59 (24.5)	13 (23.6)
	50–64	1317 (49.4)	472 (51.8)	88 (50.6)	275 (49.8)	118 (49.0)	30 (54.5)
	65–79	496 (18.6)	145 (15.9)	24 (13.8)	130 (23.6)	54 (22.4)	11 (20.0)
	80 and above	81 (3.0)	28 (3.1)	3 (1.7)	21 (3.8)	8 (3.3)	0 (0.0)
Gender (n) (%)	Female	732 (27.4)	261 (28.6)	39 (22.4)	119 (21.5)	49 (20.3)	12 (21.8)
	Male	1936 (72.6)	650 (71.4)	135 (77.6)	433 (74.8)	192 (79.7)	43 (78.2)

CVD:Cardiovascular disease

(Patient demographics was not captured in Payer 2 database)

Across all payers and cohorts, nearly 68%–80% of T2DM patients had one CVD event and 19%–31% had multiple CVD events, during the index period. The most frequently occurring CVD in the T2DM patients was coronary artery disease (CAD) (40%–80%) followed by stroke and angina (10%–20%).

### 3.2. HCRU claims and cost by the encounter

**Fig 2:**
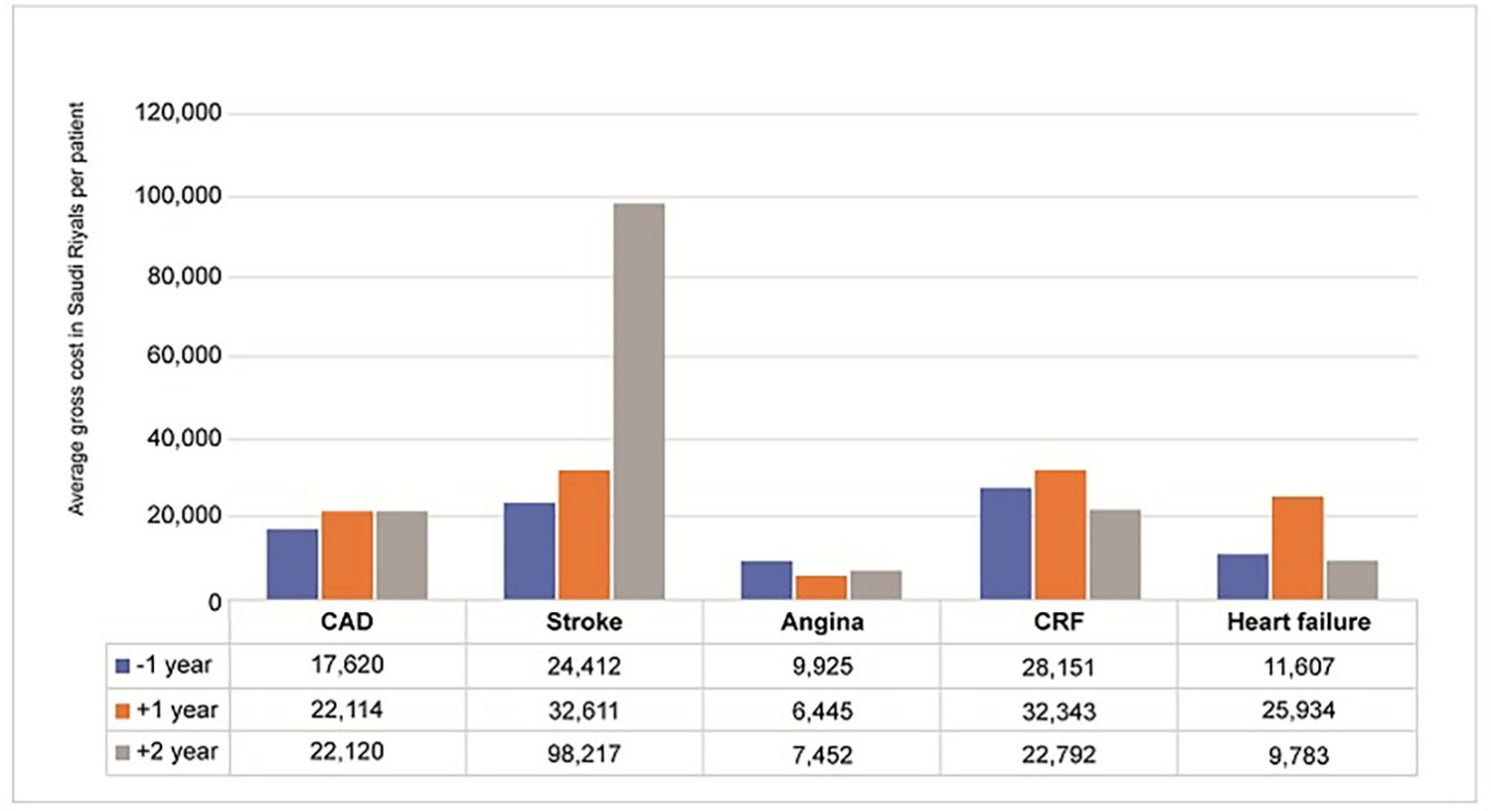
HCRU costs for inpatient claims during 2-year follow-up period for single CVD and renal events (Payer 1, Cohort 2).

**Fig 3:**
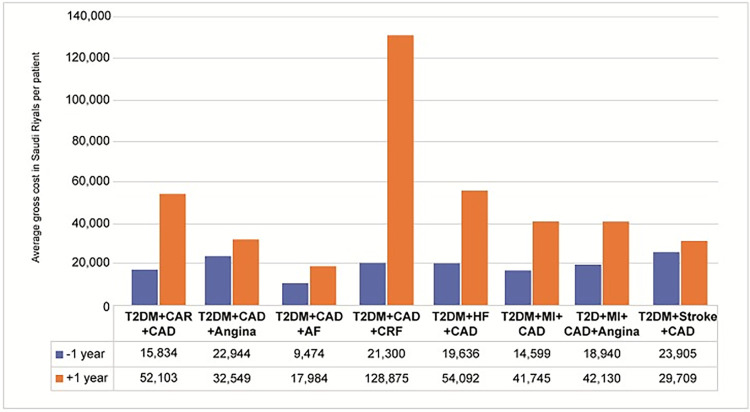
HCRU cost for inpatient claims for multiple CV and renal events during 1-year follow-up period (Payer 1, Cohort 1).

The average HCRU visits in the inpatient category for both all-cause and disease cause for all the three payers ranged from 1 to 2 visits (Table 3, S1–S5 Tables). A proportional increase in inpatient claims and cost was noted during 1-year and 2-year follow-up period compared to pre-index 1-year period, for both single and multiple CVD events in T2DM patients (Fig 2, Table 3). The increase in inpatient claims and cost was more significant for disease-specific claims as compared to all-cause claims (S3–S5 Tables). In T2DM patients with CRF, inpatient claims and cost was high for both all-cause and disease-specific HCRU. In T2DM patients with multiple CVD events, inpatient claims and cost was high in patients with coprevalent CAD and CRF(Fig 3). Outpatient utilization and costs for both single and multiple CV events and for CRF was higher during 1-year follow-up period compared to pre-index 1-year period (S3–S5 Tables).

**Table 3:** Comparison of inpatient and out-patient pre-index and post-index all-cause cost (Payer 1).

	Cohort 1						Cohort 2									Cohort 3											
**All- cause**	**Pre-Index 1 Yr**			**Post-Index 1 Yr**			**Pre-Index 1 Yr**			**Post-Index 1 Yr**			**Post-Index 2 Yr**			**Pre-Index 1 Yr**			**Post-Index 1 Yr**			**Post-Index 2 Yr**			**Post-Index 3 Yr**		
	**N**	**HCRU**	**Cost**	**N**	**HCRU**	**Cost**	**N**	**HCRU**	**Cost**	**N**	**HCRU**	**Cost**	**N**	**HCRU**	**Cost**	**N**	**HCRU**	**Cost**	**N**	**HCRU**	**Cost**	**N**	**HCRU**	**Cost**	**N**	**HCRU**	**Cost**
**Inpatient**																											
**T2DM with one CVD212,754**																											
T2DM+CAD	201	2	21,045	279	1	22,988	84	2	17,620	106	1	22,114	97	2	22,120	22	2	19,549	31	1	23,104	26	2	28,772	23	3	41,873
T2DM+Stroke or TIA	40	1	18,792	71	2	25,321	15	1	24,412	19	1	32,611	13	3	98,217	1	1	8,544	3	1	15,174	4	2	50,360	3	1	11,845
T2DM+Angina	15	1	8,348	23	1	10,841	3	2	9,925	3	1	6,445	4	1	7,452												
Others*	60	10	113,653	80	12	153,604	22	6	72,835	20	5	75,817	26	8	111,275	26	5	58,855	34	4	47,830	30	4	48,451	24	4	42,043
**T2DM with multiple CVD** ^**$**^ **399,185**																											
T2DM+ CAD**+** Angina	15	1	22,944	73	2	32,549	7	1	33,018	24	2	31,437	14	2	25,885				4	2	22,027	2	2	48,618			
T2DM+MI+ CAD	12	1	19,636	23	2	540,92	5	1	17,985	25	1	29,392	12	2	14,614				6	1	33,389	4	2	13,678	3	1	15,560
T2DM+Stroke or TIA+ CAD	17	2	23,905	49	2	29,709	8	2	26,391	25	2	24,851	20	2	42,002	4	3	20,963	7	3	31,673	5	1	22,852	6	2	15,558
T2DM + Heart failure + CAD	16	1	14,599	72	1	41,745	5	1	24,657	8	3	37,892	8	2	37,666	2	1	5,136	1	1	4,408	1	1	13,030	1	1	4,635
**Out patient**																											
**T2DM with one CVD119,033**																											
T2DM+CAD	1,802	15	9,997	1,802	16	10,609	775	15	10,385	775	16	10,959	775	17	12,011	178	15	10,824	178	16	10,883	178	19	13,547	178	15	10,311
T2DM+Stroke or TIA	297	16	9,252	297	18	10,475	105	14	8,537	105	15	8,936	105	16	10,829	17	14	7,898	17	12	6,889	17	20	10,175	17	16	8,425
T2DM+Angina	219	15	7,669	219	14	7,075	75	17	6,452	75	17	7,284	75	17	6,601	8	19	5,178	8	16	5,541	8	20	6,172	8	16	5,598
Others	327	147	83,831	349	150	90,874	100	117	77,836	100	115	74,785	100	113	88,461	15	66	42,035	15	65	49,149	15	82	74,248	15	62	48,230
**T2DM with multiple CVD** ^**$**^																											
T2DM+ CAD**+** Angina	160	15	7,876	160	17	10,756	67	15	8,342	67	17	11,219	67	19	11,720	16	18	9,074	16	20	11,301	16	22	13,137	16	16	10,013
T2DM + MI + CAD	159	12	5,867	159	17	10,946	57	12	6,596	57	17	13,029	57	17	11,206	10	15	8,167	10	21	14,302	10	25	16,231	10	20	13,951
T2DM+Stroke or TIA+ CAD	141	16	11,335	141	22	15,741	71	15	11,654	71	19	14,703	71	20	15,662	23	17	10,820	23	22	15,857	23	24	18,395	23	20	14,752
T2DM + Heart failure + CAD	67	15	10,937	67	20	19,354	33	15	9,633	33	20	13,491	33	17	11,720	6	12	11,464	6	17	19,446	6	15	14,937	6	12	14,484

CAD = Coronary artery diseases, CVD = Cardiovascular disease, HCRU = Healthcare cost utilization, N = Number of patients, T2DM = Type 2 diabetes mellitus, TIA = Transient ischemic attack

Others*- Atrial fibrillation, cardiac ischemia, Chronic renal failure, Coronary Arterial Revascularization, Dysrhythmia, Heart Failure, Myocardial infarction, Other Cardiovascular Disease, Periphery vascular disease

$—Only the most prevalent Multiple CVD complications of T2DM are included

### 3.3 HCRU and cost by activity

Medication and consultation costs dominated the visit claims while costs for claims related to medication and procedure were highest for T2DM patients with both single and multiple CVD events, across all payers. Services costs was significantly higher during post-index period in T2DM patients with comorbidities such as CRF and CAD (Table 4; S6–S23 Tables).

**Table 4:** HCRU disease-specific claims and costs for various activities during 2-year follow-up period (Payer 3, Cohort 2).

Sum of Patient_Count									
	Pre-Index 1 Yr			Post-Index 1 Yr			Post-Index 2 Yr		
Payer 3	Disease Cause			Disease Cause			Disease Cause		
Cohort 2	N	HCRU	Cost	N	HCRU	Cost	N	HCRU	Cost
**T2DM With One CVD**									
** T2DM+Angina**									
Medication	138	4	3,287	134	5	5,073	134	4	3,375
Procedure	130	3	2,814	130	4	4,280	126	3	2,011
Consultation	147	4	637	138	5	961	134	4	557
Consumables	13	3	367	29	3	376	19	2	244
Services	9	1	1,744	19	1	1,816	4	1	71
** T2DM+Chronic renal failure1**									
Medication	64	4	5,456	64	6	8,192	67	6	5,258
Procedure	63	3	3,659	65	5	8,948	66	5	7,171
Consultation	67	4	962	66	6	1,648	70	5	1,104
Consumables	12	3	1,247	15	3	1,081	17	2	370
Services	7	1	1,388	16	2	12,824	12	3	5,038
Others				3	1	43	6	1	19
** T2DM+Coronary Artery Disease**									
Medication	347	4	3,656	341	6	5,651	333	5	3,829
Procedure	276	3	2,644	290	4	5,159	270	3	2,416
Consultation	347	4	642	343	6	927	329	5	563
Consumables	45	3	850	56	3	447	47	2	291
** T2DM+Stroke or TIA**									
Medication	148	5	4,227	155	6	6,020	145	5	3,966
Procedure	142	3	3,064	140	4	5,116	139	3	2,877
Consultation	158	5	876	155	6	1,607	150	5	839
Consumables	24	3	339	28	3	522	33	3	368
Services	17	2	4,950	21	2	2,409	21	1	2,681
Others	9	1	34	7	1	20	5	1	20
**T2DM With Multiple CVD**									
** **Coronary Arterial Revascularization+T2DM+Coronary Artery Disease									
Medication	21	5	6,052	23	7	7,628	22	7	6,326
Procedure	20	4	11,133	23	4	9,305	21	4	11,968
Consultation	23	5	902	23	7	1,442	22	7	1,337
Consumables	3	2	5,434	2	2	3,917	1	2	318
Services	4	1	1,424	7	2	2,855	6	2	1,250
Others	5	1	63	2	1	11	2	1	0
Coronary Arterial Revascularization+T2DM+Coronary Artery Disease+ Angina									
Medication	8	4	6,437	8	7	10,310	8	8	8,824
Procedure	8	3	4,716	6	5	17,844	5	4	12,887
Consultation	8	5	998	8	7	1,166	8	6	1,307
Consumables				2	1	101	1	4	588
Services	1	1	315	1	2	1,500	1	3	9,656
Others				1	2	10,800	1	1	0
T2DM+Coronary Artery Disease+ Angina									
Medication	72	5	4,404	75	8	7,611	72	6	4,702
Procedure	58	4	2,773	67	5	15,304	64	4	7,410
Consultation	71	5	648	74	8	1,347	72	6	792
Consumables	7	2	365	12	3	4,879	12	3	981
Services	12	2	1,214	33	2	5,372	17	1	2,353
Others	3	1	12	10	1	1,811	5	2	216
T2DM+Coronary Artery Disease+ Chronic renal failure									
Medication	13	4	4,326	14	7	9,129	13	5	7,474
Procedure	12	3	4,145	12	5	6,635	12	4	7,362
Consultation	13	4	790	14	6	2,298	13	5	1,120
Consumables	1	1	225	2	2	414	5	1	136
Services	3	1	3,395	5	2	9,217	7	2	7,447

T2DM: Type 2 diabetes mellitus

## 4. Discussion

The alarming rise in the burden of T2DM and associated complications in Middle East countries is a cause of concern. There is limited data in the literature regarding the initial and follow-up HCRU costs associated with complications from CVD and renal disease in T2DM patients in these countries. To our knowledge, this real-world study is the first of its kind to evaluate the initial and follow-up HCRU costs associated with CVD and renal complications, in patients with T2DM in the Saudi population.

This study showed that patients with T2DM in the age group of 50–64 years had an increased incidence of CVD complications, in line with published literature [16, 17]. The incidence of CVD complications was higher among males (72–78%) as compared to females (22–28%), as observed in previous studies [6].Similar to earlier evaluations, study findings indicate that majority of T2DM patients with CRF belonged to age group 50–64 and nearly 76–78% were males [18, 19]. Since this was a private paid insurance, expatriates contributed to 76% of claims across all 3 payer databases studied.

Nearly 68–80% of patients with T2DM had at least one CVD event and CRF during the study period, and multiple CVD complications were noted in 19-31% of patients across all three payers. In a cross-sectional observation study conducted in Gulf, the most prevalent complications associated with T2DM was CKD (44.3%) and CVD (17.3%) and were found to increase substantially with age [20]. In a retrospective study, 97.5% of T2DM patients had atleast one comorbid condition and 88.5% had atleast two; CKD was reported in 24.1% patients and CVD in 21.6% patients and this study also noted an increasing trend in prevalence of comorbidities with advancing age [21]. From these findings we can infer that burden of disease due to comorbid conditions increases with age and progression of disease.

Looking into the HCRU and cost burden, we observed that overall, in majority of T2DM patients with CVD, there was a gradual increase in average HCRU and cost over the 3-year post-index period compared with the 1-year pre-index period. A significant increase in the inpatient numbers and visits for both all-cause and disease-specific causes was noted across all payers during the follow-up period. Observations from a retrospective claims database analysis indicate that higher medical costs for cardiovascular treatment was incurred during initial hospitalisation in patients with T2DM as compared to their non-diabetic counterparts [21]. Furthermore, several reports have demonstrated that CVD events have significant impact on the total and diabetes-related healthcare costs [22–25].

Current study also noted significant cost burden due to comorbid CRF in T2DM patients. Reports from a large US administrative claims database indicate that T2DM patients with newly recognised chronic kidney disease (CKD) had a high prevalence of cardiovascular comorbidities and incurred substantial HCRU and cost. The study emphasised on effective interventions for slowing the progression of CKD [26]. In yet another retrospective study, which estimated incremental all-cause HCRU and costs between T2DM patients who experienced CVD and renal complications vs. T2DM patients without any complications, significant disease and cost burden was reported in T2DM patients with complications [27]. Therefore payers, providers, and policy makers should collaboratively focus on disease management strategies and interventions that aim at reducing comorbidity-related hospitalisations and thereby healthcare costs, in T2DM patients with complications.

Of note, this is the largest study of its kind in the Middle East that assessed the cost of CVDs and renal complications in patients with T2DM. Nonetheless, certain limitations in the study may have led to bias while interpreting the results. Even though the study investigators tried to reduce the bias in the data, biases related to data duplication, possible misclassification, residual confounding bias, and selection bias may still exist. Another limitation is the retrospective nature of the study. It is worth mentioning that the claims data mostly reflected the expatriate population since all Saudi citizens are provided free healthcare mainly covered by the MOH.

## 5. Conclusion

The current analyses shed light on the substantial economic burden in patients with T2DM with comorbid CVD and renal diseases. The study observed a greater frequency of hospitalization in patients with T2DM with associated CVD and renal comorbidities. The findings also suggest a gradual increase in average healthcare resource cost over the 1-year post-index period compared with the 1-year pre-index period.

Evidence suggests that glucagon-like peptide-1 (GLP-1) agonists are associated with reduced risk of CVS and renal events and sodium-glucose-cotransporter-2 (SGLT2) have improved renal outcomes in patients with T2DM. Therefore, early initiation of these T2DM therapies would reduce long-term cardiovascular and renal outcomes and reduce the associated costs in patients with T2DM.

## Supporting information

S1 Table Comparison of inpatient and outpatient pre-index and post-index all-cause cost (Payer 2). (DOCX)

S2 Table Comparison of inpatient and outpatient pre-index and post-index all-cause cost (Payer 3). (DOCX)

S3 Table Comparison of inpatient and outpatient pre-index and post-index disease-specific cause cost (Payer 1). (DOCX)

S4 Table Comparison of inpatient and outpatient pre-index and post-index disease-specific cost (Payer 2). (DOCX)

S5 Table Comparison of inpatient and outpatient pre-index and post-index disease-specific cause cost (Payer 3). (DOCX)

S6 Table Comparison of pre-index and post-index all-cause cost for various activities (Payer 1, Cohort 1). (DOCX)

S7 Table Comparison of pre-index and post-index all-cause cost for various activities (Payer 1, Cohort 2). (DOCX)

S8 Table Comparison of pre-index and post-index all-cause cost for various activities (Payer 1, Cohort 3). (DOCX)

S9 Table Comparison of pre-Index and post-index all-cause cost for various activities (Payer 2, Cohort 1). (DOCX)

S10 Table Comparison of pre-index and post-index all-cause cost for various activities (Payer 2, Cohort 2). (DOCX)

S11 Table Comparison of pre-index and post-index all-cause cost for various activities (Payer 2, Cohort 3). (DOCX)

S12 Table Comparison of pre-index and post-index all-cause cost for various activities (Payer 3, Cohort 1). (DOCX)

S13 Table Comparison of pre-index and post-index all-cause cost for various activities (Payer 3, Cohort 2). (DOCX)

S14 Table Comparison of pre-index and post-index all-cause cost for various activities (Payer 3, Cohort 3). (DOCX)

S15 Table Comparison of pre-index and post-index disease-specific cause cost for various activities (Payer 1, Cohort 1). (DOCX)

S16 Table Comparison of pre-index and post-index disease-specific cause cost for various activities (Payer 1, Cohort 2). (DOCX)

S17 Table Comparison of pre-index and post-index disease-specific cause cost for various activities (Payer 1, Cohort 3). (DOCX)

S18 Table Comparison of pre-index and post-index disease-specific cause cost for various activities (Payer 2, Cohort 1). (DOCX)

S19 Table Comparison of pre-index and post-index disease-specific cause cost for various activities (Payer 2, Cohort 2). (DOCX)

S20 Table Comparison of pre-index and post-index disease-specific cause cost for various activities (Payer 2, Cohort 3). (DOCX)

S21 Table Comparison of pre-index and post-index disease-specific cause cost for various activities (Payer 3, Cohort 1). (DOCX)

S22 Table Comparison of pre-index and post-index disease-specific cause cost for various activities (Payer 3, Cohort 2). (DOCX)

S23 Table Comparison of pre-index and post-index disease-specific cause cost for various activities (Payer 3, Cohort 3). (DOCX)

## References

[1] Saeedi P, Petersohn I, Salpea P, Malanda B, Karuranga S, Unwin N, et al. Global and regional diabetes prevalence estimates for 2019 and projections for 2030 and 2045: Results from the International Diabetes Federation Diabetes Atlas, 9(th) edition. Diabetes Res Clin Pract. 2019;157:107843. doi: 10.1016/j.diabres.2019.107843 31518657

[2] Hu FB. Globalization of diabetes: the role of diet, lifestyle, and genes. Diabetes Care. 2011;34(6):1249–57. doi: 10.2337/dc11-0442 21617109 PMC3114340

[3] Federation ID. About Diabetes 2020. Available from: https://www.idf.org/aboutdiabetes/type-2-diabetes.html.

[4] Federation. ID. Middle East and North Africa [Available from: https://www.idf.org/our-network/regions-members/middle-east-and-north-africa/diabetes-in-mena.html.

[5] Robert AA, Al Dawish MA, Braham R, Musallam MA, Al Hayek AA, Al Kahtany NH. Type 2 Diabetes Mellitus in Saudi Arabia: Major Challenges and Possible Solutions. Curr Diabetes Rev. 2017;13(1):59–64. doi: 10.2174/1573399812666160126142605 26813972

[6] Einarson TR, Acs A, Ludwig C, Panton UH. Prevalence of cardiovascular disease in type 2 diabetes: a systematic literature review of scientific evidence from across the world in 2007–2017. Cardiovasc Diabetol. 2018;17(1):83. doi: 10.1186/s12933-018-0728-6 29884191 PMC5994068

[7] S E. Diabetes and its associated cardiovascular complications in the Arabian Gulf: Challenges and Opportunities. J Clin Exp Cardiolog. 2020;11(1):1–5.

[8] Einarson TR, Acs A, Ludwig C, Panton UH. Economic Burden of Cardiovascular Disease in Type 2 Diabetes: A Systematic Review. Value Health. 2018;21(7):881–90. doi: 10.1016/j.jval.2017.12.019 30005761

[9] Artime E, Romera I, Diaz-Cerezo S, Delgado E. Epidemiology and Economic Burden of Cardiovascular Disease in Patients with Type 2 Diabetes Mellitus in Spain: A Systematic Review. Diabetes Ther. 2021;12(6):1631–59. doi: 10.1007/s13300-021-01060-8 33942247 PMC8179862

[10] American Diabetes A. 9. Pharmacologic Approaches to Glycemic Treatment: Standards of Medical Care in Diabetes-2021. Diabetes Care. 2021;44(Suppl 1):S111–S24. doi: 10.2337/dc21-S009 33298420

[11] Chahine G BJ, Assouad P, Abi Chaker S. The $68 billion challenge, quantifying and tackling the burden of chronic diseases in the GCC. Booz & Co; 2013: Booz & Company; 2013.

[12] Davidson JA. SGLT2 inhibitors in patients with type 2 diabetes and renal disease: overview of current evidence. Postgrad Med. 2019;131(4):251–60. doi: 10.1080/00325481.2019.1601404 30929540

[13] Vupputuri S, Kimes TM, Calloway MO, Christian JB, Bruhn D, Martin AA, et al. The economic burden of progressive chronic kidney disease among patients with type 2 diabetes. J Diabetes Complications. 2014;28(1):10–6. doi: 10.1016/j.jdiacomp.2013.09.014 24211091

[14] Walston S, Al-Harbi Y, Al-Omar B. The changing face of healthcare in Saudi Arabia. Ann Saudi Med. 2008;28(4):243–50. doi: 10.5144/0256-4947.2008.243 18596400 PMC6074349

[15] Al-Hanawi MK, Alsharqi O, Almazrou S, Vaidya K. Healthcare Finance in the Kingdom of Saudi Arabia: A Qualitative Study of Householders’ Attitudes. Appl Health Econ Health Policy. 2018;16(1):55–64. doi: 10.1007/s40258-017-0353-7 28933057 PMC5797208

[16] Alwakeel JS, Sulimani R, Al-Asaad H, Al-Harbi A, Tarif N, Al-Suwaida A, et al. Diabetes complications in 1952 type 2 diabetes mellitus patients managed in a single institution in Saudi Arabia. Ann Saudi Med. 2008;28(4):260–6. doi: 10.5144/0256-4947.2008.260 18596402 PMC6074352

[17] Shah AD, Langenberg C, Rapsomaniki E, Denaxas S, Pujades-Rodriguez M, Gale CP, et al. Type 2 diabetes and incidence of cardiovascular diseases: a cohort study in 1.9 million people. Lancet Diabetes Endocrinol. 2015;3(2):105–13. 25466521 10.1016/S2213-8587(14)70219-0 PMC4303913

[18] Satyavani K, Kothandan H, Jayaraman M, Viswanathan V. Direct costs associated with chronic kidney disease among type 2 diabetic patients in India. Indian J Nephrol. 2014;24(3):141–7. doi: 10.4103/0971-4065.132000 25120290 PMC4127832

[19] Low S, Lim SC, Zhang X, Wang J, Yeo SJD, Yeoh LY, et al. Medical costs associated with chronic kidney disease progression in an Asian population with type 2 diabetes mellitus. Nephrology (Carlton). 2019;24(5):534–41. 30141833 10.1111/nep.13478

[20] Al-Ozairi E, Jallo MK, Hafidh K, Alhajeri DM, Ashour T, Mahmoud EFN, et al. Prevalence of Cardiovascular and Renal Co-morbidities in Patients with Type 2 Diabetes in the Gulf, a Cross-sectional Observational Study. Diabetes Ther. 2021;12(4):1193–207. doi: 10.1007/s13300-021-01038-6 33694092 PMC7994503

[21] Iglay K, Hannachi H, Joseph Howie P, Xu J, Li X, Engel SS, et al. Prevalence and co-prevalence of comorbidities among patients with type 2 diabetes mellitus. Curr Med Res Opin. 2016;32(7):1243–52. doi: 10.1185/03007995.2016.1168291 26986190

[22] Straka RJ, Liu LZ, Girase PS, DeLorenzo A, Chapman RH. Incremental cardiovascular costs and resource use associated with diabetes: an assessment of 29,863 patients in the US managed-care setting. Cardiovasc Diabetol. 2009;8:53. doi: 10.1186/1475-2840-8-53 19781099 PMC2762466

[23] Ward A, Alvarez P, Vo L, Martin S. Direct medical costs of complications of diabetes in the United States: estimates for event-year and annual state costs (USD 2012). J Med Econ. 2014;17(3):176–83. doi: 10.3111/13696998.2014.882843 24410011

[24] Ringborg A, Yin DD, Martinell M, Stalhammar J, Lindgren P. The impact of acute myocardial infarction and stroke on health care costs in patients with type 2 diabetes in Sweden. Eur J Cardiovasc Prev Rehabil. 2009;16(5):576–82. doi: 10.1097/HJR.0b013e32832d193b 19491686

[25] Mody R, Kalsekar I, Kavookjian J, Iyer S, Rajagopalan R, Pawar V. Economic impact of cardiovascular co-morbidity in patients with type 2 diabetes. J Diabetes Complications. 2007;21(2):75–83. doi: 10.1016/j.jdiacomp.2006.02.005 17331855

[26] Folkerts K, Petruski-Ivleva N, Kelly A, Fried L, Blankenburg M, Gay A, et al. Annual health care resource utilization and cost among type 2 diabetes patients with newly recognized chronic kidney disease within a large U.S. administrative claims database. J Manag Care Spec Pharm. 2020;26(12):1506–16. doi: 10.18553/jmcp.2020.26.12.1506 33251992 PMC10391265

[27] Annavarapu S, Ghosh S, Li Y, Moretz C, Shetty S, Prewitt T. Health care resource utilization among patients with T2D and cardiovascular-, heart failure-, or renal-related hospitalizations. Am J Manag Care. 2020;26(6):e166–e71. doi: 10.37765/ajmc.2020.43491 32549065

