# Supplementary material for: Cost of cardiovascular diseases and renal complications in people with type 2 diabetes mellitus in the Kingdom of Saudi Arabia: A retrospective analysis of claims database
Source: PLoS One. 2022 Oct 20;17(10):e0273836. doi: 10.1371/journal.pone.0273836 (PMC9584438; doi:10.1371/journal.pone.0273836)
Supplement: S1 Table — (DOCX) [file pone.0273836.s001.docx]

### S1 Table: Comparison of in-patient and out-patient pre-index and post-index all-cause cost (Payer 2)

|  | **Cohort 1** | | | | | | **Cohort 2** | | | | | | | | | **Cohort 3** | | | | | | | | | | | |
| --- | --- | --- | --- | --- | --- | --- | --- | --- | --- | --- | --- | --- | --- | --- | --- | --- | --- | --- | --- | --- | --- | --- | --- | --- | --- | --- | --- |
| **All- cause** | **Pre-Index 1 Yr** | | | **Post-Index 1 Yr** | | | **Pre-Index 1 Yr** | | | **Post-Index 1 Yr** | | | **Post-Index 2 Yr** | | | **Pre-Index 1 Yr** | | | **Post-Index 1 Yr** | | | **Post-Index 2 Yr** | | | **Post-Index 3 Yr** | | |
|  | **N** | **HCRU** | **Cost** | **N** | **HCRU** | **Cost** | **N** | **HCRU** | **Cost** | **N** | **HCRU** | **Cost** | **N** | **HCRU** | **Cost** | **N** | **HCRU** | **Cost** | **N** | **HCRU** | **Cost** | **N** | **HCRU** | **Cost** | **N** | **HCRU** | **Cost** |
| **In-patient** | | | | | | | | | | | | | | | | | | | | | | | | | | | |
| **T2DM with one CVD212,754** | | | | | | | | | | | | | | | | | | | | | | | | | | | |
| T2DM+CAD | 175 | 2 | 29,074 | 256 | 2 | 27,845 | 43 | 1 | 22,350 | 72 | 2 | 29,603 | 43 | 2 | 17,319 | 3 | 1 | 38,865 | 5 | 1 | 11,218 | 5 | 2 | 22,047 | 1 | 1 | 5,903 |
| T2DM+Stroke or TIA | 43 | 2 | 47,390 | 74 | 2 | 39,616 | 13 | 1 | 23,759 | 19 | 2 | 15,555 | 10 | 1 | 10,975 | 1 | 1 | 86,648 | 1 | 1 | 607 | 1 | 1 | 6,676 | 2 | 2 | 8,785 |
| T2DM+Angina | 21 | 1 | 9,103 | 33 | 2 | 16,418 | 7 | 1 | 13,199 | 10 | 2 | 8,659 | 7 | 1 | 2,704 | 2 | 2 | 7,976 |  |  |  | 1 | 1 | 6,718 |  |  |  |
| Others* | 73 | 13 | 203,728 | 89 | 17 | 344,928 | 20 | 8 | 135,718 | 24 | 14 | 113,238 | 18 | 11 | 115,524 | 2 | 1 | 5,525 | 2 | 13 | 149,640 | 2 | 5 | 53,382 | 2 | 12 | 132,090 |
| **T2DM with multiple CVD^$^399,185** | | | | | | | | | | | | | | | | | | | | | | | | | | | |
| T2DM+ CAD**+** Angina | 19 | 1 | 15,901 | 44 | 2 | 39,161 | 7 | 1 | 15,844 | 16 | 2 | 39,025 | 14 | 2 | 27,223 | 1 | 2 | 8,499 | 2 | 3 | 75,726 | 3 | 1 | 25,242 | 1 | 6 | 10,895 |
| T2DM+MI+ CAD | 10 | 2 | 16,529 | 32 | 2 | 48,673 | 4 | 1 | 15,778 | 15 | 2 | 38,112 | 6 | 2 | 16,665 |  |  |  | 1 | 1 | 25,949 | 1 | 1 | 0 |  |  |  |
| T2DM+Stroke or TIA+ CAD | 11 | 3 | 42,873 | 27 | 2 | 34,127 | 4 | 2 | 15,930 | 11 | 2 | 21,369 | 8 | 2 | 15,509 |  |  |  |  |  |  |  |  |  |  |  |  |
| T2DM + Heart failure + CAD | 12 | 2 | 27,721 | 24 | 3 | 76,533 | 3 | 2 | 17,463 | 9 | 2 | 40,180 | 3 | 3 | 46,817 | 1 | 1 | 24,408 | 1 | 4 | 121,934 | 1 | 4 | 13,284 |  |  |  |
| **Out patient** | | | | | | | | | | | | | | | | | | | | | | | | | | | |
| **T2DM with one CVD119,033** | | | | | | | | | | | | | | | | | | | | | | | | | | | |
| T2DM+CAD | 780 | 19 | 10,867 | 780 | 19 | 9,950 | 246 | 18 | 10,442 | 246 | 22 | 11,719 | 246 | 17 | 8,082 | 23 | 17 | 6,538 | 23 | 24 | 10,064 | 23 | 24 | 10,642 | 23 | 16 | 6,784 |
| T2DM+Stroke or TIA | 189 | 18 | 9,039 | 187 | 19 | 10,663 | 58 | 17 | 8,821 | 58 | 20 | 10,884 | 58 | 16 | 8,751 | 7 | 21 | 9,558 | 7 | 21 | 7,075 | 7 | 21 | 8,076 | 7 | 13 | 6,131 |
| T2DM+Angina | 133 | 17 | 7,864 | 133 | 17 | 7,883 | 41 | 16 | 6,901 | 41 | 20 | 8,210 | 41 | 16 | 6,443 | 5 | 21 | 9,069 | 5 | 19 | 8,323 | 5 | 18 | 8,562 | 5 | 15 | 7,275 |
| Others* | 240 | 141 | 77,294 | 240 | 135 | 89,698 | 65 | 119 | 74,043 | 65 | 142 | 125,378 | 65 | 122 | 93,884 | 6 | 34 | 27,107 | 6 | 36 | 60,027 | 6 | 50 | 105,717 | 6 | 27 | 53,344 |
| **T2DM with multiple CVD^$^** | | | | | | | | | | | | | | | | | | | | | | | | | | | |
| T2DM+ CAD**+** Angina | 82 | 17 | 8,457 | 82 | 21 | 10,307 | 32 | 18 | 8,946 | 32 | 23 | 11,489 | 32 | 21 | 11,436 | 4 | 25 | 11,186 | 4 | 33 | 15,377 | 4 | 26 | 14,017 | 4 | 20 | 4,107 |
| T2DM + MI + CAD | 37 | 15 | 8,303 | 37 | 19 | 11,049 | 18 | 17 | 9,219 | 18 | 20 | 10,392 | 18 | 15 | 7,067 | 1 | 4 | 18,550 | 1 | 9 | 2,681 | 1 | 20 | 2,160 | 1 | 5 | 829 |
| T2DM+Stroke or TIA+ CAD | 41 | 20 | 15,416 | 41 | 25 | 17,849 | 21 | 24 | 19,639 | 21 | 28 | 20,265 | 21 | 25 | 18,918 | 2 | 29 | 21,341 | 2 | 38 | 25,255 | 2 | 28 | 20,738 | 2 | 13 | 10,881 |
| T2DM + Heart failure + CAD | 34 | 19 | 12,466 | 34 | 22 | 19,454 | 13 | 21 | 10,605 | 13 | 25 | 14,526 | 13 | 14 | 9,683 | 1 | 20 | 13,150 | 1 | 42 | 20,196 | 1 | 43 | 48,191 | 1 | 46 | 38,624 |

CAD:Coronary artery diseases;CVD:Cardiovascular disease; HCRU:Healthcare cost utilization; N:Number of patients; T2DM:Type 2 diabetes mellitus;TIA:Transient ischemic attack

Others*- Atrial fibrillation, cardiac ischemia, Chronic renal failure, Coronary Arterial Revascularization, Dysrhythmia, Heart Failure, Myocardial infarction, Other Cardiovascular Disease, Periphery vascular disease

$ - Only the most prevalent Multiple CVD complications of T2DM are included
