# Supplementary material for: Cost of cardiovascular diseases and renal complications in people with type 2 diabetes mellitus in the Kingdom of Saudi Arabia: A retrospective analysis of claims database
Source: PLoS One. 2022 Oct 20;17(10):e0273836. doi: 10.1371/journal.pone.0273836 (PMC9584438; doi:10.1371/journal.pone.0273836)
Supplement: S2 Table — (DOCX) [file pone.0273836.s002.docx]

### S2 Table:Comparison of in-patient and out-patient pre-index and post-index all-cause cost (Payer 3)

|  | **Cohort 1** | | | | | | **Cohort 2** | | | | | | | | | **Cohort 3** | | | | | | | | | | | |
| --- | --- | --- | --- | --- | --- | --- | --- | --- | --- | --- | --- | --- | --- | --- | --- | --- | --- | --- | --- | --- | --- | --- | --- | --- | --- | --- | --- |
| **All- cause** | **Pre-Index 1 Yr** | | | **Post-Index 1 Yr** | | | **Pre-Index 1 Yr** | | | **Post-Index 1 Yr** | | | **Post-Index 2 Yr** | | | **Pre-Index 1 Yr** | | | **Post-Index 1 Yr** | | | **Post-Index 2 Yr** | | | **Post-Index 3 Yr** | | |
|  | **N** | **HCRU** | **Cost** | **N** | **HCRU** | **Cost** | **N** | **HCRU** | **Cost** | **N** | **HCRU** | **Cost** | **N** | **HCRU** | **Cost** | **N** | **HCRU** | **Cost** | **N** | **HCRU** | **Cost** | **N** | **HCRU** | **Cost** | **N** | **HCRU** | **Cost** |
| **In-patient** | | | | | | | | | | | | | | | | | | | | | | | | | | | |
| **T2DM with one CVD212,754** | | | | | | | | | | | | | | | | | | | | | | | | | | | |
| T2DM+CAD | 202 | 1 | 29,276 | 187 | 2 | 37,660 | 71 | 1 | 31,479 | 69 | 2 | 34,089 | 64 | 2 | 30,893 | 8 | 2 | 23,133 | 11 | 3 | 27,142 | 9 | 2 | 20,166 | 3 | 2 | 44,626 |
| T2DM+Stroke or TIA | 112 | 2 | 30,369 | 144 | 2 | 44,332 | 39 | 1 | 24,207 | 48 | 2 | 30,907 | 33 | 3 | 38,230 | 5 | 1 | 31,157 | 7 | 1 | 7,616 | 5 | 2 | 23,551 | 6 | 1 | 28,745 |
| T2DM+Angina | 54 | 2 | 21,605 | 73 | 2 | 27,080 | 14 | 2 | 24,363 | 20 | 2 | 19,391 | 20 | 1 | 16,673 | 3 | 1 | 5,580 | 4 | 2 | 22,936 | 7 | 2 | 15,777 | 5 | 2 | 26,142 |
| Others* | 185 | 15 | 244,257 | 175 | 19 | 497,639 | 63 | 12 | 216,870 | 67 | 18 | 361,547 | 48 | 11 | 240,015 | 10 | 9 | 161,107 | 15 | 10 | 224,704 | 12 | 9 | 130378 | 10 | 12 | 193,478 |
| **T2DM with multiple CVD^$^399,185** | | | | | | | | | | | | | | | | | | | | | | | | | | | |
| T2DM+ CAD**+** Angina | 43 | 1 | 23,203 | 99 | 2 | 47,576 | 13 | 2 | 28,944 | 35 | 2 | 38,287 | 18 | 2 | 39,485 | 1 | 1 | 5,150 | 3 | 1 | 41,369 | 2 | 2 | 15,663 | 5 | 3 | 38,806 |
| T2DM+MI+ CAD | 11 | 1 | 23,482 | 24 | 2 | 49,815 | 6 | 1 | 24,001 | 10 | 1 | 32,668 | 2 | 3 | 37,685 | 2 | 2 | 38,715 | 3 | 2 | 63,022 |  |  |  | 1 | 1 | 6,908 |
| T2DM+Stroke or TIA+ CAD | 20 | 2 | 39,963 | 29 | 2 | 44,682 | 5 | 1 | 19,361 | 9 | 1 | 24,370 | 9 | 3 | 198,839 | 1 | 1 | 16,819 | 1 | 1 | 51,009 |  |  |  |  |  |  |
| T2DM + Heart failure + CAD | 18 | 2 | 51,268 | 27 | 2 | 68,471 | 4 | 1 | 37,909 | 9 | 1 | 57,367 | 6 | 1 | 21,202 | 1 | 1 | 14,771 | 1 | 1 | 8,307 |  |  |  | 1 | 1 | 12,553 |
| **Out patient** | | | | | | | | | | | | | | | | | | | | | | | | | | | |
| **T2DM with one CVD119,033** | | | | | | | | | | | | | | | | | | | | | | | | | | | |
| T2DM+CAD | 1,052 | 16 | 14,398 | 1,052 | 18 | 16,982 | 369 | 17 | 15,337 | 369 | 20 | 20,018 | 369 | 16 | 12,632 | 59 | 16 | 9,526 | 59 | 20 | 19,642 | 59 | 19 | 13,420 | 59 | 13 | 8,410 |
| T2DM+Stroke or TIA | 524 | 17 | 17,249 | 524 | 21 | 21,375 | 171 | 18 | 19,804 | 171 | 23 | 26,679 | 171 | 18 | 16,503 | 32 | 15 | 16,894 | 32 | 22 | 33,284 | 32 | 20 | 20,468 | 32 | 15 | 13,785 |
| T2DM+Angina | 458 | 17 | 13,758 | 458 | 18 | 15,687 | 156 | 16 | 14,426 | 156 | 19 | 20,408 | 156 | 15 | 11,691 | 37 | 14 | 10,754 | 37 | 20 | 25,425 | 37 | 17 | 16,535 | 37 | 13 | 10,981 |
| Others* | 634 | 162 | 164,649 | 634 | 172 | 189,519 | 215 | 173 | 162,857 | 215 | 204 | 246,280 | 215 | 145 | 142,338 | 46 | 179 | 120,681 | 46 | 234 | 285,094 | 46 | 162 | 170,165 | 46 | 136 | 126,529 |
| **T2DM with multiple CVD^$^** | | | | | | | | | | | | | | | | | | | | | | | | | | | |
| T2DM+ CAD**+** Angina | 43 | 1 | 23,203 | 99 | 2 | 47,576 | 76 | 19 | 18,810 | 76 | 25 | 28,886 | 76 | 18 | 16,480 | 11 | 21 | 21,201 | 11 | 21 | 44,510 | 11 | 19 | 20,789 | 11 | 16 | 18,368 |
| T2DM + MI + CAD | 11 | 1 | 23,482 | 24 | 2 | 49,815 | 9 | 10 | 10,228 | 9 | 21 | 22,780 | 9 | 13 | 11,193 | 5 | 14 | 11,752 | 5 | 23 | 33,801 | 5 | 18 | 16,507 | 5 | 15 | 12,054 |
| T2DM+Stroke or TIA+ CAD | 20 | 2 | 39,963 | 29 | 2 | 44,682 | 24 | 19 | 24,382 | 24 | 27 | 37,684 | 24 | 22 | 30,240 | 4 | 22 | 15,152 | 4 | 28 | 31,262 | 4 | 26 | 20,477 | 4 | 10 | 7,589 |
| T2DM + Heart failure + CAD | 18 | 2 | 51,268 | 27 | 2 | 68,471 | 25 | 17 | 18,907 | 25 | 25 | 28,895 | 25 | 21 | 22,049 | 3 | 21 | 18,492 | 3 | 29 | 35,148 | 3 | 23 | 16,962 | 3 | 20 | 13,085 |

CAD:Coronary artery diseases;CVD:Cardiovascular disease;HCRU:Healthcare cost utilization; N:Number of patients;T2DM:Type 2 diabetes mellitus; TIA:Transient ischemic attack

Others*- Atrial fibrillation, cardiac ischemia, Chronic renal failure, Coronary Arterial Revascularization, Dysrhythmia, Heart Failure, Myocardial infarction, Other Cardiovascular Disease, Periphery vascular disease

$ - Only the most prevalent Multiple CVD complications of T2DM are included
