# Supplementary material for: Cost of cardiovascular diseases and renal complications in people with type 2 diabetes mellitus in the Kingdom of Saudi Arabia: A retrospective analysis of claims database
Source: PLoS One. 2022 Oct 20;17(10):e0273836. doi: 10.1371/journal.pone.0273836 (PMC9584438; doi:10.1371/journal.pone.0273836)
Supplement: S3 Table — (DOCX) [file pone.0273836.s003.docx]

### S3 Table :Comparison of in-patient and out-patient pre-index and post-index disease-specific cause cost (Payer 1)

|  | **Cohort 1** | | | | | | **Cohort 2** | | | | | | | | | **Cohort 3** | | | | | | | | | | | |
| --- | --- | --- | --- | --- | --- | --- | --- | --- | --- | --- | --- | --- | --- | --- | --- | --- | --- | --- | --- | --- | --- | --- | --- | --- | --- | --- | --- |
| **Disease-specific**  **cause** | **Pre-Index 1 Yr** | | | **Post-Index 1 Yr** | | | **Pre-Index 1 Yr** | | | **Post-Index 1 Yr** | | | **Post-Index 2 Yr** | | | **Pre-Index 1 Yr** | | | **Post-Index 1 Yr** | | | **Post-Index 2 Yr** | | | **Post-Index 3 Yr** | | |
|  | **N** | **HCRU** | **Cost** | **N** | **HCRU** | **Cost** | **N** | **HCRU** | **Cost** | **N** | **HCRU** | **Cost** | **N** | **HCRU** | **Cost** | **N** | **HCRU** | **Cost** | **N** | **HCRU** | **Cost** | **N** | **HCRU** | **Cost** | **N** | **HCRU** | **Cost** |
| **In-patient** | | | | | | | | | | | | | | | | | | | | | | | | | | | |
| **T2DM with one CVD212,754** | | | | | | | | | | | | | | | | | | | | | | | | | | | |
| T2DM+CAD | 26 | 1 | 5,178 | 85 | 1 | 16,787 | 9 | 1 | 925 | 33 | 1 | 16,894 | 11 | 1 | 16,071 | 1 | 1 | 5,239 | 8 | 1 | 15,831 | 3 | 1 | 3,891 | 8 | 1 | 21,861 |
| T2DM+Stroke or TIA | 7 | 1 | 4,850 | 27 | 1 | 27,196 | 4 | 1 | 3,924 | 6 | 1 | 54,655 | 2 | 1 | 156 |  |  |  |  |  |  |  |  |  | 1 | 1 | 6,052 |
| T2DM+Angina | 1 | 1 | 450 | 2 | 1 | 38 |  |  |  |  |  |  | 1 | 1 | 2,113 |  |  |  |  |  |  |  |  |  |  |  |  |
| Others* | 15 | 6 | 17,980 | 35 | 11 | 77,513 | 6 | 4 | 19,496 | 8 | 5 | 36,600 | 12 | 5 | 26,905 |  |  |  | 1 | 5 | 39,673 | 3 | 2 | 2197 |  |  | 41,881 |
| **T2DM with multiple CVD^$^399,185** | | | | | | | | | | | | | | | | | | | | | | | | | | | |
| T2DM+ CAD**+** Angina | 5 | 1 | 652 | 62 | 1 | 28,434 | 2 | 1 | 6 | 23 | 1 | 25,630 | 5 | 1 | 35,272 |  |  |  | 4 | 1 | 20,905 | 1 | 1 | 80,000 |  |  |  |
| T2DM+MI+ CAD | 1 | 1 | 17 | 61 | 1 | 38,015 | 1 | 1 | 17 | 20 | 1 | 32,996 | 5 | 1 | 13,428 |  |  |  | 5 | 1 | 38,559 | 2 | 1 | 10,486 | 1 | 1 | 14,331 |
| T2DM+Stroke or TIA+ CAD | 5 | 1 | 5,420 | 29 | 2 | 24,162 | 2 | 2 | 7,552 | 13 | 1 | 28,919 | 10 | 2 | 43,920 | 1 | 1 | 1,000 | 4 | 2 | 29,803 | 1 | 1 | 8,583 | 2 | 1 | 7,740 |
| T2DM + Heart failure + CAD | 2 | 1 | 665 | 16 | 1 | 35,558 | 1 | 1 | 1,100 | 4 | 2 | 7,142 | 5 | 1 | 26,648 |  |  |  | 1 | 1 | 4,401 | 1 | 1 | 165 |  |  |  |
| **Out patient** | | | | | | | | | | | | | | | | | | | | | | | | | | | |
| **T2DM with one CVD119,033** | | | | | | | | | | | | | | | | | | | | | | | | | | | |
| T2DM+CAD | 1,602 | 5 | 3,812 | 1,608 | 6 | 4,940 | 697 | 5 | 3,930 | 695 | 6 | 5,272 | 680 | 6 | 5,925 | 164 | 5 | 3,754 | 162 | 6 | 5,582 | 157 | 7 | 6,238 | 148 | 6 | 5,262 |
| T2DM+Stroke or TIA | 247 | 5 | 3,038 | 238 | 6 | 4,676 | 83 | 4 | 3,030 | 82 | 5 | 4,136 | 80 | 5 | 4,290 | 14 | 5 | 2,969 | 14 | 6 | 4,586 | 13 | 7 | 4,296 | 13 | 6 | 4,138 |
| T2DM+Angina | 182 | 4 | 2,453 | 156 | 5 | 3,161 | 66 | 4 | 2,401 | 51 | 5 | 3,793 | 54 | 6 | 3,472 | 7 | 5 | 2,964 | 6 | 6 | 3,138 | 6 | 7 | 3,189 | 7 | 4 | 3,351 |
| Others* | 297 | 34 | 26,123 | 295 | 46 | 37,255 | 91 | 36 | 32,326 | 88 | 38 | 34,437 | 86 | 42 | 46,430 | 14 | 15 | 9,975 | 13 | 22 | 20,386 | 13 | 38 | 34,384 | 11 | 36 | 32,549 |
| **T2DM with multiple CVD^$^** | | | | | | | | | | | | | | | | | | | | | | | | | | | |
| T2DM+ CAD**+** Angina | 146 | 5 | 3,330 | 153 | 6 | 5,064 | 62 | 5 | 3,840 | 66 | 6 | 5,413 | 60 | 7 | 5,942 | 14 | 4 | 3,240 | 16 | 6 | 5,021 | 15 | 7 | 6,702 | 15 | 7 | 5,879 |
| T2DM + MI + CAD | 135 | 5 | 2,820 | 155 | 8 | 6,401 | 46 | 4 | 3,072 | 54 | 8 | 7,340 | 52 | 7 | 6,834 | 8 | 5 | 3,546 | 10 | 8 | 7,162 | 10 | 10 | 8,667 | 10 | 8 | 6,938 |
| T2DM+Stroke or TIA+ CAD | 121 | 5 | 3,564 | 135 | 7 | 6,577 | 63 | 4 | 3,802 | 68 | 6 | 6,040 | 69 | 7 | 6,922 | 22 | 4 | 3,595 | 23 | 6 | 6,164 | 22 | 8 | 8,170 | 18 | 8 | 9,048 |
| T2DM + Heart failure + CAD | 59 | 4 | 4,122 | 66 | 8 | 6,984 | 30 | 4 | 3,292 | 33 | 8 | 6,817 | 28 | 7 | 5,945 | 5 | 3 | 5,542 | 6 | 10 | 11,422 | 6 | 6 | 7,175 | 6 | 5 | 7,127 |

CAD:Coronary artery diseases;CVD:Cardiovascular disease;HCRU:Healthcare cost utilization; N:Number of patients;T2DM:Type 2 diabetes mellitus; TIA:Transient ischemic attack

Others*- Atrial fibrillation, cardiac ischemia, Chronic renal failure, Coronary Arterial Revascularization, Dysrhythmia, Heart Failure, Myocardial infarction, Other Cardiovascular Disease, Periphery vascular disease

$ - Only the most prevalent Multiple CVD complications of T2DM are included
