# Supplementary material for: Cost of cardiovascular diseases and renal complications in people with type 2 diabetes mellitus in the Kingdom of Saudi Arabia: A retrospective analysis of claims database
Source: PLoS One. 2022 Oct 20;17(10):e0273836. doi: 10.1371/journal.pone.0273836 (PMC9584438; doi:10.1371/journal.pone.0273836)
Supplement: S4 Table — (DOCX) [file pone.0273836.s004.docx]

### S4 Table :Comparison of in-patient and out-patient pre-index and post-index disease-specific cause cost (Payer 2)

|  | **Cohort 1** | | | | | | **Cohort 2** | | | | | | | | | **Cohort 3** | | | | | | | | | | | |
| --- | --- | --- | --- | --- | --- | --- | --- | --- | --- | --- | --- | --- | --- | --- | --- | --- | --- | --- | --- | --- | --- | --- | --- | --- | --- | --- | --- |
| **Disease-specific**  **cause** | **Pre-Index 1 Yr** | | | **Post-Index 1 Yr** | | | **Pre-Index 1 Yr** | | | **Post-Index 1 Yr** | | | **Post-Index 2 Yr** | | | **Pre-Index 1 Yr** | | | **Post-Index 1 Yr** | | | **Post-Index 2 Yr** | | | **Post-Index 3 Yr** | | |
|  | **N** | **HCRU** | **Cost** | **N** | **HCRU** | **Cost** | **N** | **HCRU** | **Cost** | **N** | **HCRU** | **Cost** | **N** | **HCRU** | **Cost** | **N** | **HCRU** | **Cost** | **N** | **HCRU** | **Cost** | **N** | **HCRU** | **Cost** | **N** | **HCRU** | **Cost** |
| **In-patient** | | | | | | | | | | | | | | | | | | | | | | | | | | | |
| **T2DM with one CVD212,754** | | | | | | | | | | | | | | | | | | | | | | | | | | | |
| T2DM+CAD | 6 | 1 | 14,152 | 123 | 1 | 27,351 |  |  |  | 2 | 1 | 1,555 | 1 | 1 | 0 |  |  |  | 1 | 1 | 11,921 | 2 | 2 | 19,808 | 1 | 1 | 5,903 |
| T2DM+Stroke or TIA | 3 | 1 | 36,914 | 32 | 1 | 33,477 | 1 | 1 | 6,397 | 6 | 1 | 2,811 |  |  |  |  |  |  |  |  |  |  |  |  | 2 | 1 | 1,669 |
| T2DM+Angina | 1 | 2 | 2,635 | 13 | 1 | 1,697 |  |  |  | 2 | 1 | 1,555 | 1 | 1 | 0 |  |  |  |  |  |  |  |  |  |  |  |  |
| Others* | 9 | 6 | 35,603 | 35 | 10 | 382,846 | 2 | 1 | 10927 | 12 | 6 | 40727 | 10 | 3 | 40,726 |  |  |  | 1 | 1 | 14,806 | 1 | 2 | 41,406 | 1 | 7 | 97,171 |
| **T2DM with multiple CVD^$^399,185** | | | | | | | | | | | | | | | | | | | | | | | | | | | |
| T2DM+ CAD**+** Angina | 2 | 1 | 10,800 | 32 | 2 | 35,546 | 1 | 1 | 21,427 | 12 | 2 | 40,324 | 8 | 2 | 28,357 |  |  |  | 2 | 1 | 44,576 | 1 | 1 | 0 | 1 | 3 | 10,895 |
| T2DM+MI+ CAD | 2 | 2 | 3,565 | 26 | 2 | 43,143 |  |  |  | 12 | 2 | 38,602 | 3 | 2 | 27,550 |  |  |  | 1 | 1 | 25,949 | 1 | 1 | 0 |  |  |  |
| T2DM+Stroke or TIA+ CAD | 2 | 2 | 30,630 | 18 | 2 | 25,498 |  |  |  | 8 | 1 | 18,092 | 3 | 1 | 13,293 |  |  |  |  |  |  |  |  |  |  |  |  |
| T2DM + Heart failure + CAD | 2 | 2 | 23,099 | 16 | 2 | 63,496 | 1 | 1 | 5,811 | 4 | 1 | 31,998 | 2 | 2 | 5,229 |  |  |  |  |  |  | 1 | 1 | 8,189 |  |  |  |
| **Out patient** | | | | | | | | | | | | | | | | | | | | | | | | | | | |
| **T2DM with one CVD119,033** | | | | | | | | | | | | | | | | | | | | | | | | | | | |
| T2DM+CAD | 727 | 5 | 3,274 | 692 | 6 | 4,162 | 39 | 3 | 1,849 | 28 | 5 | 3,219 | 27 | 5 | 2,301 | 22 | 4 | 2,692 | 22 | 6 | 4,578 | 18 | 6 | 5,373 | 18 | 5 | 3,626 |
| T2DM+Stroke or TIA | 170 | 4 | 2,792 | 161 | 6 | 3,920 | 50 | 4 | 2,448 | 48 | 6 | 3,582 | 41 | 5 | 3,478 | 7 | 5 | 3,048 | 7 | 5 | 2,812 | 6 | 7 | 5,052 | 5 | 7 | 5,460 |
| T2DM+Angina | 120 | 4 | 2,310 | 96 | 5 | 3,200 | 39 | 3 | 1,849 | 28 | 5 | 3,219 | 27 | 5 | 2,301 | 5 | 3 | 2,362 | 3 | 5 | 5,965 | 4 | 3 | 2,376 | 4 | 3 | 2,314 |
| Others* | 214 | 36 | 23,327 | 202 | 39 | 34,719 | 59 | 36 | 21,044 | 53 | 40 | 45,173 | 50 | 42 | 44,293 | 6 | 9 | 6621 | 5 | 14 | 12965 | 6 | 12 | 52,972 | 6 | 11 | 24,919 |
| **T2DM with multiple CVD^$^** | | | | | | | | | | | | | | | | | | | | | | | | | | | |
| T2DM+ CAD**+** Angina | 74 | 4 | 2,432 | 81 | 7 | 4,308 | 28 | 4 | 2,479 | 32 | 7 | 4,555 | 32 | 6 | 4,389 | 4 | 4 | 1,447 | 4 | 6 | 2,942 | 4 | 8 | 3,117 | 3 | 5 | 1,406 |
| T2DM + MI + CAD | 36 | 5 | 2,680 | 35 | 9 | 6,607 | 18 | 5 | 2,331 | 17 | 9 | 5,297 | 18 | 5 | 3,002 | 1 | 1 | 237 | 1 | 7 | 2,373 | 1 | 15 | 1,452 | 1 | 2 | 256 |
| T2DM+Stroke or TIA+ CAD | 37 | 4 | 3,245 | 38 | 8 | 6,602 | 19 | 4 | 4,068 | 18 | 9 | 8,266 | 21 | 6 | 5,910 | 2 | 4 | 3,304 | 2 | 7 | 6,353 | 2 | 5 | 4,683 | 2 | 4 | 3,684 |
| T2DM + Heart failure + CAD | 32 | 6 | 5,033 | 34 | 9 | 11,385 | 13 | 6 | 3,466 | 13 | 8 | 6,185 | 12 | 5 | 3,124 | 1 | 2 | 1,356 | 1 | 11 | 3,237 | 1 | 2 | 1,609 | 1 | 14 | 17,835 |

CAD:Coronary artery diseases;CVD:Cardiovascular disease;HCRU:Healthcare cost utilization; N:Number of patients;T2DM:Type 2 diabetes mellitus; TIA:Transient ischemic attack

Others*- Atrial fibrillation, cardiac ischemia, Chronic renal failure, Coronary Arterial Revascularization, Dysrhythmia, Heart Failure, Myocardial infarction, Other Cardiovascular Disease, Periphery vascular disease

$ - Only the most prevalent Multiple CVD complications of T2DM are included
