# Supplementary material for: Cost of cardiovascular diseases and renal complications in people with type 2 diabetes mellitus in the Kingdom of Saudi Arabia: A retrospective analysis of claims database
Source: PLoS One. 2022 Oct 20;17(10):e0273836. doi: 10.1371/journal.pone.0273836 (PMC9584438; doi:10.1371/journal.pone.0273836)
Supplement: S5 Table — (DOCX) [file pone.0273836.s005.docx]

### S5 Table :Comparison of in-patient and out-patient pre-index and post-index disease-specific cause cost (Payer 3)

|  | **Cohort 1** | | | | | | **Cohort 2** | | | | | | | | | **Cohort 3** | | | | | | | | | | | |
| --- | --- | --- | --- | --- | --- | --- | --- | --- | --- | --- | --- | --- | --- | --- | --- | --- | --- | --- | --- | --- | --- | --- | --- | --- | --- | --- | --- |
| **Disease-specific cause** | **Pre-Index 1 Yr** | | | **Post-Index 1 Yr** | | | **Pre-Index 1 Yr** | | | **Post-Index 1 Yr** | | | **Post-Index 2 Yr** | | | **Pre-Index 1 Yr** | | | **Post-Index 1 Yr** | | | **Post-Index 2 Yr** | | | **Post-Index 3 Yr** | | |
|  | **N** | **HCRU** | **Cost** | **N** | **HCRU** | **Cost** | **N** | **HCRU** | **Cost** | **N** | **HCRU** | **Cost** | **N** | **HCRU** | **Cost** | **N** | **HCRU** | **Cost** | **N** | **HCRU** | **Cost** | **N** | **HCRU** | **Cost** | **N** | **HCRU** | **Cost** |
| **In-patient** | | | | | | | | | | | | | | | | | | | | | | | | | | | |
| **T2DM with one CVD212,754** | | | | | | | | | | | | | | | | | | | | | | | | | | | |
| T2DM+CAD | 12 | 1 | 23,138 | 31 | 1 | 52,746 | 6 | 1 | 30,949 | 11 | 1 | 67,000 | 4 | 1 | 31,845 |  |  |  | 1 | 1 | 17,083 | 1 | 1 | 5,163 | 1 | 1 | 90,151 |
| T2DM+Stroke or TIA | 6 | 1 | 18,682 | 30 | 1 | 20,073 | 2 | 2 | 28,651 | 5 | 1 | 11,318 | 5 | 1 | 12,233 | 1 | 1 | 6,955 | 1 | 1 | 4,793 |  |  |  |  |  |  |
| T2DM+Angina | 5 | 1 | 30,938 | 11 | 1 | 19,701 | 1 | 1 | 14,191 | 6 | 1 | 16,991 |  |  |  |  |  |  |  |  |  |  |  |  | 1 | 1 | 7,891 |
| Others* | 14 | 6 | 263,391 | 43 | 12 | 394,653 | 6 | 4 | 190,616 | 18 | 11 | 501,885 | 11 | 3 | 38415 | 2 | 2 | 123,087 | 5 | 8 | 392,958 | 2 | 2 | 45060 | 3 | 3 | 17842 |
| **T2DM with multiple CVD$399,185** | | | | | | | | | | | | | | | | | | | | | | | | | | | |
| T2DM+ CAD**+** Angina | 4 | 2 | 18,955 | 68 | 1 | 37,154 | 1 | 2 | 39,685 | 24 | 1 | 39,635 | 7 | 1 | 51,164 |  |  |  | 3 | 1 | 41,369 | 1 | 1 | 1,691 | 1 | 1 | 151 |
| T2DM+MI+ CAD | 3 | 1 | 7,638 | 12 | 1 | 66,683 | 2 | 1 | 6,313 | 4 | 1 | 40,604 |  |  |  | 1 | 1 | 4,457 | 3 | 1 | 51,639 |  |  |  | 1 | 1 | 6,908 |
| T2DM+Stroke or TIA+ CAD | 1 | 1 | 29,063 | 12 | 1 | 41,611 |  |  |  |  |  |  | 3 | 3 | 421,546 |  |  |  |  |  |  |  |  |  |  |  |  |
| T2DM + Heart failure + CAD |  |  |  | 14 | 1 | 76,325 |  |  |  | 4 | 1 | 92,559 | 1 | 2 | 26,893 |  |  |  |  |  |  | 1 | 1 | 15,952 |  |  |  |
| **Out patient** | | | | | | | | | | | | | | | | | | | | | | | | | | | |
| **T2DM with one CVD119,033** | | | | | | | | | | | | | | | | | | | | | | | | | | | |
| T2DM+CAD | 993 | 4 | 5,376 | 946 | 6 | 7,656 | 363 | 5 | 5,817 | 350 | 7 | 8,911 | 339 | 6 | 6,061 | 59 | 5 | 4,486 | 58 | 8 | 10,432 | 56 | 8 | 7,291 | 59 | 6 | 5,244 |
| T2DM+Stroke or TIA | 482 | 5 | 6,148 | 471 | 7 | 9,537 | 164 | 5 | 7,527 | 158 | 7 | 12,071 | 152 | 5 | 7,291 | 31 | 5 | 7,557 | 32 | 7 | 16,475 | 32 | 6 | 9,547 | 32 | 5 | 7,167 |
| T2DM+Angina | 428 | 4 | 5,077 | 403 | 5 | 6,806 | 148 | 4 | 6,218 | 139 | 6 | 9,443 | 139 | 5 | 5,650 | 37 | 4 | 4,698 | 34 | 6 | 12,761 | 34 | 6 | 8,735 | 34 | 5 | 6,036 |
| Others* | 597 | 40 | 55,703 | 564 | 61 | 92,126 | 206 | 47 | 60,717 | 198 | 81 | 131,822 | 202 | 50 | 65,704 | 41 | 49 | 57,276 | 40 | 88 | 122,965 | 40 | 49 | 66,407 | 40 | 45 | 55,763 |
| **T2DM with multiple CVD^$^** | | | | | | | | | | | | | | | | | | | | | | | | | | | |
| T2DM+ CAD**+** Angina | 185 | 5 | 6,278 | 187 | 8 | 10,431 | 76 | 5 | 6,598 | 76 | 9 | 13,139 | 74 | 7 | 7,629 | 11 | 6 | 7,563 | 11 | 8 | 22,964 | 11 | 6 | 8,951 | 11 | 5 | 9,180 |
| T2DM + MI + CAD | 51 | 5 | 4,502 | 54 | 9 | 10,913 | 21 | 4 | 3,306 | 22 | 8 | 10,223 | 22 | 7 | 6,617 | 5 | 3 | 2,505 | 5 | 10 | 13,190 | 5 | 6 | 6,184 | 5 | 4 | 3,716 |
| T2DM+Stroke or TIA+ CAD | 64 | 5 | 5,700 | 68 | 10 | 14,703 | 23 | 6 | 8,012 | 24 | 11 | 14,477 | 22 | 11 | 22,858 | 4 | 8 | 8,671 | 4 | 10 | 10,145 | 4 | 10 | 9,445 | 4 | 4 | 2,887 |
| T2DM + Heart failure + CAD | 67 | 5 | 5,162 | 67 | 7 | 9,256 | 25 | 5 | 5,834 | 24 | 8 | 10,493 | 23 | 7 | 7,861 | 3 | 6 | 4,212 | 3 | 8 | 12,630 | 2 | 9 | 11,101 | 3 | 7 | 5,269 |

CAD:Coronary artery diseases;CVD:Cardiovascular disease;HCRU:Healthcare cost utilization; N:Number of patients;T2DM:Type 2 diabetes mellitus; TIA:Transient ischemic attack

Others*- Atrial fibrillation, cardiac ischemia, Chronic renal failure, Coronary Arterial Revascularization, Dysrhythmia, Heart Failure, Myocardial infarction, Other Cardiovascular Disease, Periphery vascular disease

$ - Only the most prevalent Multiple CVD complications of T2DM are included
