# Supplementary material for: Cost of cardiovascular diseases and renal complications in people with type 2 diabetes mellitus in the Kingdom of Saudi Arabia: A retrospective analysis of claims database
Source: PLoS One. 2022 Oct 20;17(10):e0273836. doi: 10.1371/journal.pone.0273836 (PMC9584438; doi:10.1371/journal.pone.0273836)
Supplement: S6 Table — (DOCX) [file pone.0273836.s006.docx]

### S6 Table: Comparison of pre-index and post-index all-cause cost for various activities (Payer 1, Cohort 1)

|  | **Pre-Index 1 Yr** |  |  | **Post-Index 1 Yr** |  |  |
| --- | --- | --- | --- | --- | --- | --- |
| **Payer 1** | **All-Cause** | | | **All-Cause** | | |
| **Cohort 1** | **N** | **HCRU** | **Cost** | **N** | **HCRU** | **Cost** |
| **T2DM With One CVD2,486** | | | | | | |
| T2DM+Angina**6** | | | | | | |
| Medication | 218 | 11 | 4,455 | 219 | 10 | 3,651 |
| Procedure | 217 | 7 | 2,730 | 215 | 6 | 3,324 |
| Consultation | 219 | 11 | 671 | 219 | 11 | 625 |
| Consumables | 69 | 2 | 681 | 73 | 2 | 564 |
| Services | 66 | 2 | 289 | 64 | 2 | 625 |
| Others | 70 | 2 | 400 | 75 | 2 | 898 |
| T2DM+Atrial fibrillation**18,726** | | | | | | |
| Medication | 57 | 11 | 4,390 | 57 | 12 | 6,496 |
| Procedure | 57 | 7 | 5,985 | 55 | 6 | 6,038 |
| Consultation | 57 | 11 | 1,127 | 57 | 12 | 1,270 |
| Consumables | 18 | 3 | 2,639 | 34 | 2 | 1,026 |
| Services | 19 | 3 | 1,417 | 26 | 2 | 2,440 |
| Others | 22 | 2 | 1,046 | 26 | 3 | 1,457 |
| T2DM+cardiac ischemia | | | | | | |
| Medication | 1 | 9 | 2,136 | 1 | 11 | 2,367 |
| Procedure | 1 | 3 | 710 | 1 | 2 | 155 |
| Consultation | 1 | 9 | 210 | 1 | 11 | 161 |
| Consumables |  |  |  | 1 | 1 | 400 |
| Services |  |  |  |  |  |  |
| Others |  |  |  | 1 | 1 | 86 |
| T2DM+Chronic renal failure | | | | | | |
| Medication | 134 | 13 | 7,198 | 134 | 14 | 8,766 |
| Procedure | 134 | 9 | 8,806 | 134 | 10 | 18,012 |
| Consultation | 134 | 13 | 1,488 | 133 | 12 | 1,547 |
| Consumables | 62 | 2 | 840 | 72 | 3 | 2,950 |
| Services | 47 | 2 | 2,282 | 48 | 3 | 5,012 |
| Others | 62 | 3 | 707 | 69 | 3 | 734 |
| T2DM+Coronary Arterial Revascularization | | | | | | |
| Medication | 1 | 15 | 2,553 | 1 | 16 | 3,193 |
| Procedure | 1 | 10 | 4,926 | 1 | 5 | 1,630 |
| Consultation | 1 | 15 | 1,418 | 1 | 17 | 1,260 |
| Consumables |  |  |  | 1 | 1 | 110 |
| Services |  |  |  |  |  |  |
| Others | 1 | 6 | 818 | 1 | 1 | 50 |
| T2DM+Coronary Artery Disease | | | | | | |
| Medication | 1,802 | 11 | 5,480 | 1,798 | 11 | 5,760 |
| Procedure | 1,771 | 7 | 4,942 | 1,758 | 7 | 6,064 |
| Consultation | 1,790 | 11 | 980 | 1,796 | 11 | 1,054 |
| Consumables | 731 | 2 | 850 | 793 | 2 | 1,239 |
| Services | 513 | 2 | 1,276 | 494 | 2 | 1,568 |
| Others | 837 | 2 | 701 | 856 | 3 | 1,016 |
| T2DM+Dysrhythmia**0**  **13,525** | | | | | | |
| Medication | 27 | 13 | 6,115 | 27 | 13 | 6,768 |
| Procedure | 26 | 7 | 3,969 | 27 | 7 | 4,099 |
| Consultation | 27 | 13 | 810 | 27 | 13 | 985 |
| Consumables | 8 | 3 | 703 | 13 | 2 | 430 |
| Services | 7 | 1 | 893 | 6 | 1 | 320 |
| Others | 16 | 2 | 442 | 7 | 3 | 923 |
| T2DM+Heart Failure | | | | | | |
| Medication | 70 | 13 | 6,920 | 70 | 13 | 7,223 |
| Procedure | 69 | 8 | 7,382 | 69 | 8 | 6,642 |
| Consultation | 70 | 13 | 1,163 | 70 | 13 | 1,250 |
| Consumables | 22 | 3 | 889 | 31 | 2 | 902 |
| Services | 23 | 3 | 1,799 | 25 | 3 | 2,129 |
| Others | 34 | 2 | 1,078 | 27 | 3 | 3,207 |
| T2DM+Myocardial infarction | | | | | | |
| Medication | 37 | 8 | 1,926 | 37 | 8 | 2,395 |
| Procedure | 36 | 5 | 1,968 | 35 | 6 | 7,497 |
| Consultation | 37 | 8 | 430 | 37 | 9 | 681 |
| Consumables | 13 | 1 | 649 | 12 | 1 | 950 |
| Services | 8 | 2 | 396 | 11 | 2 | 1,442 |
| Others | 12 | 1 | 295 | 15 | 3 | 648 |
| T2DM+Other Cardiovascular Disease | | | | | | |
| Medication | 14 | 12 | 4,163 | 14 | 11 | 2,982 |
| Procedure | 13 | 8 | 5,884 | 13 | 7 | 3,736 |
| Consultation | 14 | 12 | 1,101 | 13 | 11 | 792 |
| Consumables | 2 | 2 | 227 | 2 | 1 | 725 |
| Services | 2 | 1 | 773 | 3 | 1 | 53 |
| Others | 4 | 2 | 602 | 4 | 2 | 6,229 |
| T2DM+Periphery vascular disease | | | | | | |
| Medication | 8 | 13 | 5,236 | 8 | 12 | 6,324 |
| Procedure | 8 | 9 | 7,592 | 8 | 9 | 5,871 |
| Consultation | 8 | 13 | 1,442 | 8 | 13 | 1,725 |
| Consumables | 4 | 2 | 806 | 6 | 3 | 675 |
| Services | 1 | 1 | 10 | 2 | 2 | 213 |
| Others | 3 | 1 | 297 | 4 | 2 | 1,986 |
| T2DM+Stroke or TIA | | | | | | |
| Medication | 296 | 12 | 4,106 | 297 | 12 | 4,627 |
| Procedure | 290 | 7 | 5,237 | 289 | 8 | 7,580 |
| Consultation | 295 | 12 | 1,038 | 295 | 12 | 1,334 |
| Consumables | 137 | 2 | 1,191 | 153 | 3 | 1,375 |
| Services | 95 | 2 | 2,137 | 110 | 3 | 5,017 |
| Others | 134 | 3 | 692 | 156 | 3 | 1,208 |
| **T2DM With Multiple CVD** | | | | | | |
| T2DM+Coronary Arterial Revascularization+Coronary Artery Disease | | | | | | |
| Medication | 23 | 10 | 3,718 | 23 | 11 | 8,205 |
| Procedure | 22 | 6 | 3,790 | 23 | 7 | 11,899 |
| Consultation | 23 | 10 | 633 | 23 | 12 | 1,018 |
| Consumables | 5 | 3 | 3,546 | 11 | 2 | 916 |
| Services | 5 | 1 | 187 | 8 | 5 | 4,934 |
| Others | 9 | 2 | 453 | 10 | 2 | 321 |
| T2DM+Coronary Artery Disease+Angina | | | | | | |
| Medication | 159 | 11 | 3,920 | 160 | 13 | 6,714 |
| Procedure | 158 | 7 | 4,388 | 158 | 8 | 13,385 |
| Consultation | 159 | 11 | 812 | 160 | 13 | 1,110 |
| Consumables | 65 | 2 | 1,629 | 88 | 2 | 4,854 |
| Services | 43 | 2 | 534 | 78 | 2 | 3,136 |
| Others | 71 | 2 | 418 | 86 | 3 | 884 |
| T2DM+Coronary Artery Disease+Atrial fibrillation | | | | | | |
| Medication | 48 | 12 | 5,176 | 48 | 14 | 7,157 |
| Procedure | 48 | 7 | 4,783 | 48 | 9 | 9,111 |
| Consultation | 47 | 14 | 1,176 | 48 | 15 | 1,568 |
| Consumables | 25 | 2 | 862 | 29 | 2 | 1,189 |
| Services | 13 | 2 | 496 | 19 | 2 | 2,176 |
| Others | 23 | 2 | 518 | 26 | 3 | 1,055 |
| T2DM+Coronary Artery Disease+Chronic renal failure**2,242** | | | | | | |
| Medication | 41 | 13 | 8,551 | 41 | 16 | 21,021 |
| Procedure | 41 | 10 | 6,257 | 41 | 12 | 19,088 |
| Consultation | 41 | 14 | 1,537 | 41 | 15 | 2,617 |
| Consumables | 22 | 2 | 391 | 23 | 3 | 1,491 |
| Services | 14 | 3 | 1,034 | 18 | 10 | 77,189 |
| Others | 22 | 2 | 386 | 25 | 3 | 837 |
| T2DM+Heart Failure+Coronary Artery Disease**,223** | | | | | | |
| Medication | 67 | 12 | 6,438 | 67 | 16 | 13,258 |
| Procedure | 64 | 7 | 6,093 | 66 | 9 | 16,288 |
| Consultation | 67 | 11 | 928 | 67 | 14 | 1,824 |
| Consumables | 29 | 2 | 619 | 35 | 3 | 3,953 |
| Services | 25 | 2 | 2,215 | 28 | 2 | 9,425 |
| Others | 33 | 2 | 351 | 36 | 3 | 1,475 |
| T2DM+Myocardial infarction+Coronary Artery Disease**7,592** | | | | | | |
| Medication | 158 | 9 | 3,206 | 159 | 13 | 7,730 |
| Procedure | 154 | 6 | 3,006 | 158 | 7 | 14,222 |
| Consultation | 158 | 10 | 557 | 159 | 12 | 1,105 |
| Consumables | 54 | 2 | 1,100 | 78 | 2 | 7,086 |
| Services | 38 | 2 | 669 | 67 | 2 | 5,220 |
| Others | 59 | 2 | 410 | 86 | 3 | 2,229 |
| T2DM+Myocardial infarction+Coronary Artery Disease+Angina**,216** | | | | | | |
| Medication | 52 | 12 | 5,376 | 52 | 15 | 8,380 |
| Procedure | 51 | 7 | 3,970 | 52 | 9 | 19,232 |
| Consultation | 52 | 11 | 740 | 51 | 14 | 1,399 |
| Consumables | 21 | 2 | 554 | 40 | 2 | 5,859 |
| Services | 10 | 2 | 1,854 | 29 | 2 | 4,711 |
| Others | 17 | 3 | 576 | 23 | 4 | 2,634 |
| T2DM+Stroke or TIA+Coronary Artery Disease**,492** | | | | | | |
| Medication | 141 | 12 | 5,080 | 141 | 14 | 8,514 |
| Procedure | 136 | 7 | 6,593 | 140 | 10 | 10,969 |
| Consultation | 140 | 12 | 1,280 | 141 | 15 | 2,064 |
| Consumables | 73 | 2 | 1,072 | 86 | 2 | 1,457 |
| Services | 51 | 3 | 1,535 | 66 | 4 | 6,059 |
| Others | 69 | 3 | 811 | 86 | 3 | 1,429 |
| Abbreviations: CVD=Cardiovascular disease, HCRU=Healthcare cost utilization, N=Number of patients, T2DM=Type 2 diabetes mellitus, TIA=Transient ischemic attack | | | | | | |
