# Supplementary material for: Cost of cardiovascular diseases and renal complications in people with type 2 diabetes mellitus in the Kingdom of Saudi Arabia: A retrospective analysis of claims database
Source: PLoS One. 2022 Oct 20;17(10):e0273836. doi: 10.1371/journal.pone.0273836 (PMC9584438; doi:10.1371/journal.pone.0273836)
Supplement: S7 Table — (DOCX) [file pone.0273836.s007.docx]

### S7 Table: Comparison of pre-index and post-index all-cause cost for various activities (Payer 1, Cohort 2)

| **All-Cause** | **Pre-Index 1 Yr** | | | **Post-Index 1 Yr** | | | **Post-Index 2 Yr** | | |
| --- | --- | --- | --- | --- | --- | --- | --- | --- | --- |
| **Payer 1** | **All-Cause** | | | **All-Cause** | | | **All-Cause** | | |
| **Cohort 2** | **N** | **HCRU** | **Cost** | **N** | **HCRU** | **Cost** | **N** | **HCRU** | **Cost** |
| **T2DM With One CVD98** | | | | | | | | | |
| T2DM+Angina | | | | | | | | | |
| Medication | 75 | 13 | 3,055 | 75 | 12 | 3,612 | 75 | 13 | 3,380 |
| Procedure | 75 | 7 | 2,692 | 74 | 7 | 2,777 | 74 | 8 | 2,714 |
| Consultation | 75 | 13 | 731 | 75 | 12 | 710 | 75 | 12 | 525 |
| Consumables | 23 | 2 | 751 | 27 | 3 | 606 | 25 | 2 | 446 |
| Services | 26 | 2 | 139 | 21 | 1 | 108 | 21 | 2 | 505 |
| Others | 26 | 2 | 268 | 30 | 2 | 578 | 21 | 2 | 472 |
| T2DM+Atrial fibrillation | | | | | | | | | |
| Medication | 17 | 8 | 3,126 | 17 | 10 | 3,439 | 17 | 12 | 3,693 |
| Procedure | 17 | 4 | 3,044 | 15 | 5 | 3,548 | 17 | 6 | 3,925 |
| Consultation | 17 | 9 | 821 | 17 | 12 | 1,017 | 17 | 12 | 999 |
| Consumables | 5 | 1 | 288 | 10 | 2 | 538 | 9 | 2 | 360 |
| Services | 6 | 1 | 608 | 3 | 2 | 590 | 3 | 2 | 833 |
| Others | 7 | 2 | 1,603 | 7 | 2 | 471 | 7 | 3 | 427 |
| T2DM+Chronic renal failure | | | | | | | | | |
| Medication | 44 | 14 | 8,583 | 44 | 13 | 8,380 | 44 | 16 | 9,247 |
| Procedure | 44 | 10 | 8,647 | 44 | 11 | 16,155 | 44 | 13 | 23,167 |
| Consultation | 44 | 13 | 1,577 | 44 | 11 | 1,572 | 44 | 12 | 1,525 |
| Consumables | 25 | 2 | 776 | 26 | 4 | 3,451 | 23 | 4 | 5,979 |
| Services | 16 | 2 | 3,587 | 23 | 3 | 3,127 | 21 | 3 | 3,392 |
| Others | 13 | 2 | 401 | 21 | 3 | 572 | 21 | 2 | 1,175 |
| T2DM+Coronary Artery Disease**0** | | | | | | | | | |
| Medication | 775 | 11 | 5,449 | 774 | 12 | 5,706 | 775 | 12 | 6,497 |
| Procedure | 766 | 7 | 5,061 | 764 | 7 | 6,036 | 761 | 8 | 6,222 |
| Consultation | 770 | 11 | 1,039 | 774 | 11 | 1,078 | 774 | 11 | 1,074 |
| Consumables | 307 | 2 | 747 | 333 | 2 | 1,068 | 368 | 2 | 892 |
| Services | 226 | 2 | 798 | 213 | 2 | 1,367 | 241 | 2 | 859 |
| Others | 374 | 2 | 586 | 401 | 3 | 822 | 347 | 3 | 935 |
| T2DM+Dysrhythmia**21,969** | | | | | | | | | |
| Medication | 5 | 19 | 6,893 | 5 | 21 | 4,359 | 5 | 12 | 3,878 |
| Procedure | 5 | 10 | 7,921 | 5 | 9 | 3,984 | 5 | 8 | 15,917 |
| Consultation | 5 | 19 | 906 | 5 | 19 | 913 | 5 | 12 | 921 |
| Consumables | 2 | 5 | 645 | 4 | 2 | 303 | 5 | 2 | 639 |
| Services | 4 | 1 | 945 | 3 | 1 | 64 | 1 | 1 | 614 |
| Others | 3 | 1 | 87 |  |  |  |  |  |  |
| Medication | 21 | 14 | 4,972 | 21 | 13 | 6,013 | 21 | 13 | 6,063 |
| Procedure | 21 | 9 | 7,540 | 21 | 8 | 7,511 | 21 | 8 | 6,468 |
| Consultation | 21 | 15 | 1,841 | 21 | 13 | 1,853 | 21 | 13 | 1,603 |
| Consumables | 7 | 3 | 967 | 10 | 2 | 687 | 10 | 2 | 565 |
| Services | 8 | 2 | 838 | 6 | 2 | 2,097 | 7 | 2 | 817 |
| Others | 14 | 2 | 630 | 12 | 4 | 1,295 | 9 | 5 | 1,691 |
| T2DM+Myocardial infarction**,713** | | | | | | | | | |
| Medication | 6 | 8 | 2,612 | 6 | 9 | 2,000 | 6 | 9 | 3,538 |
| Procedure | 6 | 6 | 2,697 | 6 | 8 | 6,942 | 6 | 7 | 5,290 |
| Consultation | 6 | 8 | 521 | 6 | 10 | 841 | 6 | 10 | 792 |
| Consumables | 4 | 2 | 535 | 1 | 2 | 131 | 1 | 7 | 1,215 |
| Services | 3 | 1 | 406 | 2 | 4 | 375 | 5 | 1 | 290 |
| Others | 2 | 1 | 99 | 3 | 4 | 881 | 3 | 3 | 588 |
| T2DM+Other Cardiovascular Disease**,757** | | | | | | | | | |
| Medication | 6 | 11 | 3,416 | 6 | 12 | 3,311 | 6 | 11 | 3,266 |
| Procedure | 6 | 8 | 2,781 | 6 | 6 | 3,131 | 6 | 8 | 3,150 |
| Consultation | 6 | 10 | 885 | 5 | 11 | 710 | 6 | 10 | 627 |
| Consumables | 1 | 2 | 370 | 1 | 1 | 700 | 1 | 2 | 714 |
| Services | 1 | 1 | 45 | 2 | 1 | 62 |  |  |  |
| Others | 1 | 1 | 800 |  |  |  |  |  |  |
| T2DM+Periphery vascular disease**,680** | | | | | | | | | |
| Medication | 1 | 10 | 9,173 | 1 | 4 | 6,414 | 1 | 4 | 7,913 |
| Procedure | 1 | 12 | 13,098 | 1 | 4 | 2,030 | 1 | 5 | 2,837 |
| Consultation | 1 | 13 | 770 | 1 | 3 | 90 | 1 | 3 | 90 |
| Consumables | 1 | 3 | 1,061 | 1 | 4 | 893 | 1 | 3 | 840 |
| Services |  |  |  |  |  |  |  |  |  |
| Others |  |  |  | 1 | 1 | 150 |  |  |  |
| T2DM+Stroke or TIA**629** | | | | | | | | | |
| Medication | 105 | 11 | 4,005 | 105 | 11 | 4,320 | 105 | 12 | 7,091 |
| Procedure | 102 | 7 | 5,444 | 99 | 8 | 6,889 | 103 | 8 | 10,328 |
| Consultation | 103 | 11 | 1,026 | 103 | 11 | 1,156 | 104 | 12 | 1,311 |
| Consumables | 46 | 2 | 1,513 | 46 | 2 | 1,112 | 53 | 3 | 3,760 |
| Services | 35 | 2 | 2,452 | 31 | 3 | 6,894 | 27 | 3 | 7,924 |
| Others | 49 | 2 | 526 | 55 | 3 | 698 | 46 | 3 | 1,214 |
| **T2DM With Multiple CVD** | | | | | | | | | |
| T2DM+Coronary Arterial Revascularization+Coronary Artery Disease | | | | | | | | | |
| Medication | 10 | 11 | 4,943 | 10 | 11 | 9,867 | 10 | 13 | 6,979 |
| Procedure | 9 | 7 | 4,267 | 10 | 8 | 7,584 | 9 | 9 | 7,804 |
| Consultation | 10 | 11 | 879 | 10 | 12 | 1,185 | 10 | 14 | 1,230 |
| Consumables | 2 | 6 | 993 | 3 | 3 | 443 | 2 | 3 | 1,082 |
| Services | 3 | 1 | 273 | 3 | 11 | 4,699 | 3 | 4 | 1,618 |
| Others | 3 | 1 | 131 | 5 | 2 | 402 | 2 | 2 | 729 |
| T2DM+Coronary Artery Disease+Angina**,409** | | | | | | | | | |
| Medication | 67 | 11 | 4,136 | 67 | 13 | 6,617 | 67 | 13 | 6,663 |
| Procedure | 67 | 7 | 5,231 | 66 | 8 | 11,937 | 67 | 8 | 7,504 |
| Consultation | 67 | 11 | 846 | 67 | 12 | 1,033 | 67 | 12 | 943 |
| Consumables | 29 | 2 | 2,886 | 33 | 2 | 3,761 | 37 | 2 | 1,800 |
| Services | 17 | 2 | 563 | 33 | 2 | 1,683 | 24 | 2 | 1,055 |
| Others | 32 | 2 | 390 | 35 | 3 | 768 | 30 | 3 | 1,445 |
| T2DM+Coronary Artery Disease+Atrial fibrillation | | | | | | | | | |
| Medication | 19 | 12 | 4,818 | 19 | 13 | 5,959 | 19 | 14 | 7,218 |
| Procedure | 19 | 7 | 3,758 | 19 | 9 | 7,893 | 19 | 10 | 7,395 |
| Consultation | 19 | 13 | 984 | 19 | 15 | 1,447 | 18 | 15 | 1,481 |
| Consumables | 9 | 2 | 235 | 11 | 2 | 1,800 | 14 | 2 | 769 |
| Services | 6 | 3 | 464 | 5 | 3 | 1,855 | 6 | 2 | 2,493 |
| Others | 9 | 2 | 219 | 9 | 3 | 465 | 12 | 2 | 1,020 |
| T2DM+Coronary Artery Disease+Chronic renal failure**,803** | | | | | | | | | |
| Medication | 19 | 14 | 11,799 | 19 | 17 | 31,214 | 19 | 20 | 18,025 |
| Procedure | 19 | 11 | 8,735 | 19 | 13 | 20,924 | 19 | 14 | 26,083 |
| Consultation | 19 | 15 | 1,550 | 19 | 16 | 2,925 | 19 | 19 | 3,266 |
| Consumables | 11 | 2 | 416 | 11 | 3 | 1,665 | 14 | 3 | 921 |
| Services | 9 | 3 | 1,325 | 9 | 18 | 144,181 | 11 | 3 | 11,423 |
| Others | 12 | 2 | 316 | 14 | 3 | 880 | 8 | 4 | 1,085 |
| T2DM+Heart Failure+Coronary Artery Disease | | | | | | | | | |
| Medication | 33 | 12 | 5,459 | 33 | 15 | 8,037 | 33 | 13 | 7,976 |
| Procedure | 32 | 7 | 5,560 | 33 | 9 | 10,927 | 31 | 7 | 9,113 |
| Consultation | 33 | 11 | 882 | 33 | 14 | 1,281 | 33 | 11 | 1,112 |
| Consumables | 15 | 2 | 735 | 15 | 3 | 991 | 22 | 3 | 1,535 |
| Services | 12 | 2 | 3,034 | 12 | 2 | 3,904 | 16 | 2 | 3,916 |
| Others | 17 | 2 | 389 | 16 | 4 | 1,159 | 14 | 3 | 772 |
| T2DM+Myocardial infarction+Coronary Artery Disease**25** | | | | | | | | | |
| Medication | 56 | 9 | 3,576 | 57 | 13 | 7,542 | 56 | 12 | 6,946 |
| Procedure | 55 | 5 | 3,482 | 57 | 8 | 10,809 | 54 | 7 | 5,379 |
| Consultation | 56 | 9 | 600 | 57 | 13 | 1,134 | 57 | 12 | 860 |
| Consumables | 19 | 2 | 1,198 | 27 | 2 | 7,040 | 26 | 2 | 2,189 |
| Services | 9 | 2 | 1,019 | 23 | 1 | 6,328 | 15 | 2 | 969 |
| Others | 19 | 2 | 453 | 30 | 3 | 1,040 | 24 | 3 | 682 |
| T2DM+Myocardial infarction+Coronary Artery Disease+Angina**6** | | | | | | | | | |
| Medication | 22 | 13 | 6,371 | 22 | 16 | 9,405 | 22 | 16 | 8,789 |
| Procedure | 22 | 8 | 3,453 | 22 | 10 | 12,452 | 21 | 10 | 11,657 |
| Consultation | 22 | 12 | 790 | 21 | 15 | 1,220 | 22 | 15 | 1,305 |
| Consumables | 11 | 2 | 473 | 14 | 2 | 2,653 | 13 | 3 | 7,770 |
| Services | 4 | 3 | 3,915 | 11 | 3 | 2,548 | 11 | 3 | 4,401 |
| Others | 8 | 3 | 637 | 8 | 4 | 4,935 | 8 | 5 | 7,675 |
| T2DM+Stroke or TIA+Coronary Artery Disease | | | | | | | | | |
| Medication | 71 | 11 | 5,555 | 71 | 13 | 7,945 | 71 | 14 | 8,762 |
| Procedure | 69 | 7 | 6,566 | 70 | 9 | 10,172 | 68 | 10 | 11,236 |
| Consultation | 71 | 11 | 1,206 | 71 | 14 | 1,950 | 71 | 14 | 1,888 |
| Consumables | 35 | 2 | 1,447 | 42 | 2 | 874 | 41 | 2 | 2,513 |
| Services | 28 | 3 | 1,200 | 32 | 2 | 5,623 | 21 | 3 | 8,266 |
| Others | 36 | 2 | 591 | 43 | 3 | 789 | 40 | 3 | 3,881 |
| Abbreviations: CVD=Cardiovascular disease, HCRU=Healthcare cost utilization, N=Number of patients, T2DM=Type 2 diabetes mellitus, TIA=Transient ischemic attack | | | | | | | | | |
