# Supplementary material for: Cost of cardiovascular diseases and renal complications in people with type 2 diabetes mellitus in the Kingdom of Saudi Arabia: A retrospective analysis of claims database
Source: PLoS One. 2022 Oct 20;17(10):e0273836. doi: 10.1371/journal.pone.0273836 (PMC9584438; doi:10.1371/journal.pone.0273836)
Supplement: S8 Table — (DOCX) [file pone.0273836.s008.docx]

**S8 Table: Comparison of pre-index and post-index all-cause cost for various activities (Payer 1, Cohort 3)**

| **All-Cause** | **Pre-Index 1 Yr** | | | **Post-Index 1 Yr** | | | **Post-Index 2 Yr** | | | **Post-Index 3 Yr** | | | |
| --- | --- | --- | --- | --- | --- | --- | --- | --- | --- | --- | --- | --- | --- |
| **Payer 1** | **All- Cause** |  |  | **All-Cause** |  |  | **All -Cause** |  |  | **All -Cause** |  | |  |
| **Cohort 3** | **N** | **HCRU** | **Cost** | **N** | **HCRU** | **Cost** | **N** | **HCRU** | **Cost** | **N** | **HCRU** | | **Cost** |
| **T2DM WITH ONE CVD** | | | | | | | | | | | | | |
| **T2DM+Angina** | **33** | **43** | **5,722** | **31** | **35** | **6,188** | **29** | **38** | **6,435** | **28** | **31** | | **6,152** |
| Medication | 8 | 13 | 3,046 | 8 | 12 | 3,261 | 8 | 14 | 4,137 | 8 | 11 | | 3,610 |
| Procedure | 8 | 8 | 1,555 | 8 | 7 | 1,727 | 8 | 9 | 1,670 | 8 | 7 | | 1,583 |
| Consultation | 8 | 12 | 242 | 8 | 10 | 236 | 8 | 13 | 274 | 8 | 9 | | 221 |
| Consumables | 3 | 1 | 533 | 3 | 2 | 687 | 2 | 1 | 327 | 2 | 3 | | 716 |
| Services | 4 | 7 | 197 | 3 | 2 | 97 | 3 | 1 | 28 | 2 | 1 | | 23 |
| Others | 2 | 3 | 150 | 1 | 2 | 180 |  |  |  |  |  | |  |
| **T2DM+Atrial fibrillation** | **10** | **20** | **14,758** | **10** | **26** | **10,165** | **10** | **33** | **17,577** | **8** | **35** | | **11,713** |
| Medication | 2 | 8 | 5,118 | 2 | 9 | 3,586 | 2 | 8 | 4,931 | 2 | 9 | | 5,207 |
| Procedure | 2 | 3 | 3,315 | 2 | 6 | 3,674 | 2 | 8 | 7,181 | 1 | 13 | | 4,570 |
| Consultation | 2 | 7 | 1,612 | 2 | 6 | 1,130 | 2 | 13 | 2,073 | 2 | 8 | | 1,289 |
| Consumables |  |  |  | 1 | 1 | 500 | 1 | 1 | 1,000 | 1 | 1 | | 3 |
| Services | 2 | 2 | 758 | 1 | 2 | 200 | 2 | 2 | 849 | 1 | 1 | | 100 |
| Others | 2 | 1 | 3,955 | 2 | 3 | 1,076 | 1 | 2 | 1,543 | 1 | 3 | | 545 |
| **T2DM+Chronic renal failure** | **38** | **52** | **27,946** | **37** | **58** | **29,695** | **38** | **63** | **37,779** | **32** | **48** | | **19,985** |
| Medication | 8 | 18 | 5,721 | 8 | 17 | 5,267 | 8 | 22 | 10,674 | 8 | 16 | | 4,976 |
| Procedure | 8 | 12 | 13,350 | 8 | 13 | 19,772 | 8 | 18 | 22,254 | 8 | 13 | | 13,363 |
| Consultation | 8 | 16 | 3,140 | 8 | 13 | 2,132 | 8 | 11 | 1,588 | 8 | 8 | | 818 |
| Consumables | 6 | 2 | 1,863 | 4 | 7 | 453 | 4 | 8 | 592 | 4 | 7 | | 396 |
| Services | 5 | 2 | 3,519 | 5 | 2 | 1,237 | 5 | 2 | 2,303 | 1 | 1 | | 53 |
| Others | 3 | 2 | 353 | 4 | 5 | 834 | 5 | 2 | 369 | 3 | 3 | | 379 |
| **T2DM+Coronary Artery Disease** | **743** | **35** | **14,544** | **756** | **37** | **17,092** | **785** | **41** | **19,297** | **707** | **35** | | **19,576** |
| Medication | 178 | 11 | 6,458 | 178 | 12 | 6,020 | 178 | 13 | 7,568 | 178 | 11 | | 6,367 |
| Procedure | 175 | 7 | 5,106 | 175 | 7 | 6,483 | 177 | 8 | 7,639 | 172 | 7 | | 6,706 |
| Consultation | 178 | 11 | 1,043 | 178 | 11 | 1,165 | 178 | 13 | 1,193 | 177 | 10 | | 1,085 |
| Consumables | 66 | 2 | 608 | 77 | 2 | 886 | 95 | 2 | 1,238 | 76 | 3 | | 1,760 |
| Services | 59 | 2 | 983 | 58 | 2 | 1,773 | 65 | 2 | 831 | 50 | 2 | | 2,472 |
| Others | 87 | 2 | 346 | 90 | 2 | 764 | 92 | 3 | 827 | 54 | 3 | | 1,187 |
| **T2DM+Dysrhythmia** | **6** | **26** | **6,305** | **5** | **33** | **8,578** | **4** | **29** | **10,570** | **5** | **27** | | **6,995** |
| Medication | 1 | 10 | 3,025 | 1 | 12 | 3,093 | 1 | 10 | 4,663 | 1 | 8 | | 2,867 |
| Procedure | 1 | 4 | 2,235 | 1 | 10 | 4,971 | 1 | 9 | 5,083 | 1 | 7 | | 3,546 |
| Consultation | 1 | 7 | 450 | 1 | 8 | 395 | 1 | 7 | 380 | 1 | 6 | | 302 |
| Consumables | 1 | 3 | 485 | 1 | 2 | 104 | 1 | 3 | 445 | 1 | 2 | | 160 |
| Services | 1 | 1 | 60 | 1 | 1 | 15 |  |  |  | 1 | 4 | | 120 |
| Others | 1 | 1 | 50 |  |  |  |  |  |  |  |  | |  |
| **T2DM+Heart Failure** | **19** | **32** | **11,437** | **17** | **34** | **12,144** | **17** | **47** | **21,007** | **15** | **31** | | **13,363** |
| Medication | 4 | 12 | 5,593 | 4 | 11 | 6,360 | 4 | 14 | 9,698 | 4 | 11 | | 7,079 |
| Procedure | 4 | 5 | 3,369 | 4 | 7 | 3,278 | 4 | 7 | 5,629 | 4 | 6 | | 4,330 |
| Consultation | 4 | 10 | 1,374 | 4 | 9 | 1,151 | 4 | 14 | 2,515 | 4 | 11 | | 1,000 |
| Consumables | 2 | 2 | 487 | 2 | 2 | 863 | 3 | 2 | 451 | 2 | 2 | | 597 |
| Services | 2 | 2 | 128 | 1 | 1 | 100 | 1 | 1 | 58 |  |  | |  |
| Others | 3 | 2 | 487 | 2 | 5 | 393 | 1 | 9 | 2,657 | 1 | 1 | | 359 |
| **T2DM+Stroke or TIA** | **72** | **35** | **9,077** | **69** | **28** | **10,363** | **70** | **45** | **31,805** | **68** | **33** | | **12,090** |
| Medication | 17 | 10 | 2,492 | 17 | 8 | 2,537 | 17 | 12 | 3,741 | 17 | 9 | | 2,930 |
| Procedure | 17 | 7 | 4,652 | 17 | 7 | 5,732 | 16 | 11 | 12,267 | 16 | 10 | | 6,232 |
| Consultation | 17 | 10 | 783 | 16 | 8 | 738 | 16 | 13 | 1,927 | 17 | 9 | | 1,026 |
| Consumables | 7 | 1 | 294 | 7 | 1 | 448 | 7 | 3 | 1,860 | 4 | 3 | | 424 |
| Services | 7 | 2 | 285 | 3 | 1 | 175 | 5 | 4 | 9,352 | 8 | 2 | | 1,244 |
| Others | 7 | 4 | 572 | 9 | 2 | 732 | 9 | 3 | 2,657 | 6 | 1 | | 234 |
| **T2DM WITH MULTIPLE CVD** | | | | | | | | | | | | | |
| **T2DM+Coronary Arterial Revascularization+Coronary Artery Disease** | **10** | **28** | **9,184** | **10** | **25** | **6,429** | **11** | **31** | **9,003** | **10** | **19** | | **25,882** |
| Medication | 3 | 9 | 3,046 | 3 | 9 | 3,823 | 3 | 12 | 3,308 | 3 | 8 | | 2,786 |
| Procedure | 2 | 8 | 5,187 | 3 | 4 | 1,862 | 3 | 6 | 4,405 | 3 | 3 | | 1,075 |
| Consultation | 3 | 10 | 557 | 3 | 10 | 643 | 3 | 12 | 751 | 3 | 7 | | 321 |
| Consumables |  |  |  |  |  |  |  |  |  |  |  | |  |
| Services | 2 | 1 | 395 |  |  |  | 1 | 1 | 420 |  |  | |  |
| Others |  |  |  | 1 | 1 | 100 | 1 | 1 | 119 | 1 | 1 | | 21,700 |
| **T2DM+Coronary Artery Disease+Angina** |  |  |  |  |  |  |  |  |  |  |  | |  |
| Medication | 16 | 13 | 3,733 | 16 | 15 | 5,073 | 16 | 16 | 5,847 | 16 | 11 | | 4,431 |
| Procedure | 16 | 8 | 3,744 | 16 | 9 | 8,053 | 16 | 10 | 10,918 | 16 | 8 | | 3,989 |
| Consultation | 16 | 13 | 1,064 | 16 | 15 | 1,112 | 16 | 16 | 1,161 | 16 | 11 | | 844 |
| Consumables | 4 | 4 | 1,827 | 7 | 2 | 4,711 | 10 | 2 | 399 | 6 | 2 | | 604 |
| Services | 2 | 2 | 140 | 11 | 2 | 197 | 6 | 1 | 477 | 5 | 2 | | 354 |
| Others | 6 | 1 | 157 | 9 | 3 | 666 | 9 | 3 | 1,530 | 3 | 3 | | 2,196 |
| **T2DM+Coronary Artery Disease+Atrial fibrillation** |  |  |  |  |  |  |  |  |  |  |  | |  |
| Medication | 6 | 12 | 4,534 | 6 | 13 | 5,713 | 6 | 15 | 7,716 | 6 | 14 | | 8,939 |
| Procedure | 6 | 6 | 1,717 | 6 | 9 | 7,761 | 6 | 9 | 8,455 | 6 | 8 | | 9,633 |
| Consultation | 6 | 12 | 745 | 6 | 15 | 1,032 | 5 | 17 | 1,606 | 6 | 13 | | 1,306 |
| Consumables | 2 | 2 | 83 | 4 | 1 | 762 | 5 | 2 | 1,224 | 3 | 3 | | 35,710 |
| Services | 2 | 1 | 103 | 1 | 1 | 155 | 1 | 3 | 3,401 | 3 | 3 | | 2,394 |
| Others | 3 | 1 | 90 | 2 | 3 | 483 | 3 | 3 | 2,843 | 2 | 1 | | 78 |
| **T2DM+Coronary Artery Disease+Chronic renal failure** |  |  |  |  |  |  |  |  |  |  |  | |  |
| Medication | 3 | 15 | 4,620 | 3 | 18 | 7,257 | 3 | 20 | 10,271 | 3 | 10 | | 5,849 |
| Procedure | 3 | 10 | 12,354 | 3 | 13 | 10,779 | 3 | 11 | 10,362 | 3 | 5 | | 2,373 |
| Consultation | 3 | 18 | 2,389 | 3 | 24 | 4,238 | 3 | 25 | 3,519 | 3 | 14 | | 2,440 |
| Consumables | 1 | 2 | 750 | 2 | 2 | 540 | 2 | 3 | 183 | 1 | 2 | | 130 |
| Services | 2 | 4 | 398 | 1 | 2 | 1,800 | 2 | 3 | 163 |  |  | |  |
| Others | 2 | 2 | 55 | 3 | 2 | 577 | 2 | 4 | 891 | 1 | 6 | | 8,739 |
| **T2DM+Heart Failure+Chronic renal failure** |  |  |  |  |  |  |  |  |  |  |  | |  |
| Medication | 3 | 10 | 6,117 | 3 | 8 | 6,436 | 3 | 16 | 9,621 | 3 | 6 | | 3,857 |
| Procedure | 3 | 10 | 22,832 | 3 | 13 | 20,661 | 3 | 8 | 15,008 | 3 | 4 | | 4,163 |
| Consultation | 3 | 8 | 2,044 | 3 | 6 | 1,702 | 3 | 11 | 2,675 | 3 | 6 | | 874 |
| Consumables | 2 | 1 | 230 |  |  |  | 1 | 1 | 3,557 | 1 | 2 | | 3,995 |
| Services | 3 | 1 | 1,497 | 2 | 1 | 4,090 | 2 | 1 | 5,482 |  |  | |  |
| Others | 2 | 1 | 1,015 | 2 | 10 | 997 | 2 | 5 | 1,202 | 1 | 7 | | 3,499 |
| **T2DM+Heart Failure+Coronary Artery Disease** | **29** | **27** | **13,543** | **25** | **40** | **21,402** | **26** | **33** | **17,976** | **23** | **29** | | **18,350** |
| Medication | 6 | 9 | 5,736 | 6 | 14 | 10,174 | 6 | 12 | 10,219 | 6 | 9 | | 6,796 |
| Procedure | 6 | 5 | 5,680 | 6 | 6 | 7,602 | 6 | 5 | 4,462 | 5 | 6 | | 7,984 |
| Consultation | 6 | 7 | 882 | 6 | 13 | 1,746 | 6 | 11 | 1,487 | 6 | 8 | | 982 |
| Consumables | 4 | 2 | 510 | 2 | 3 | 605 | 4 | 2 | 321 | 3 | 2 | | 632 |
| Services | 5 | 3 | 584 | 2 | 2 | 1,077 | 3 | 2 | 1,437 | 2 | 2 | | 1,094 |
| Others | 2 | 3 | 149 | 3 | 2 | 197 | 1 | 1 | 50 | 1 | 2 | | 861 |
| **T2DM+Myocardial infarction+Coronary Artery Disease** | **39** | **35** | **9,659** | **47** | **46** | **40,924** | **44** | **57** | **23,222** | **39** | **42** | | **20,335** |
| Medication | 10 | 11 | 3,118 | 10 | 16 | 8,007 | 10 | 19 | 8,382 | 10 | 15 | | 8,159 |
| Procedure | 9 | 8 | 4,430 | 10 | 9 | 18,495 | 10 | 11 | 10,014 | 10 | 9 | | 8,326 |
| Consultation | 10 | 12 | 739 | 10 | 15 | 1,054 | 10 | 19 | 1,371 | 10 | 13 | | 1,257 |
| Consumables | 4 | 2 | 426 | 5 | 2 | 10,059 | 7 | 2 | 2,111 | 6 | 3 | | 907 |
| Services | 1 | 1 | 800 | 5 | 1 | 2,830 | 3 | 1 | 800 | 2 | 1 | | 1,640 |
| Others | 5 | 1 | 146 | 7 | 3 | 478 | 4 | 5 | 544 | 1 | 1 | | 46 |
| **T2DM+Myocardial infarction+Coronary Artery Disease+Angina** | **13** | **49** | **18,915** | **15** | **36** | **31,672** | **15** | **58** | **24,225** | **15** | **52** | | **25,700** |
| Medication | 3 | 17 | 10,233 | 3 | 12 | 8,808 | 3 | 19 | 12,966 | 3 | 18 | | 15,567 |
| Procedure | 3 | 12 | 7,284 | 3 | 10 | 14,926 | 3 | 15 | 7,378 | 3 | 13 | | 7,260 |
| Consultation | 3 | 17 | 1,031 | 3 | 10 | 692 | 3 | 17 | 1,054 | 3 | 13 | | 764 |
| Consumables | 3 | 2 | 167 | 3 | 2 | 6,808 | 3 | 4 | 564 | 3 | 5 | | 792 |
| Services |  |  |  | 2 | 1 | 88 | 1 | 1 | 100 | 2 | 2 | | 1,303 |
| Others | 1 | 1 | 200 | 1 | 1 | 350 | 2 | 3 | 2,163 | 1 | 1 | | 15 |
| **T2DM+Stroke or TIA+Coronary Artery Disease** | **102** | **44** | **16,391** | **107** | **50** | **28,144** | **98** | **54** | **32,404** | **94** | **42** | | **20,734** |
| Medication | 23 | 13 | 5,532 | 23 | 16 | 10,185 | 23 | 17 | 9,374 | 23 | 13 | | 9,658 |
| Procedure | 23 | 9 | 6,227 | 23 | 11 | 11,150 | 22 | 13 | 9,507 | 22 | 11 | | 7,677 |
| Consultation | 23 | 13 | 1,400 | 23 | 15 | 1,949 | 23 | 16 | 2,284 | 23 | 12 | | 1,590 |
| Consumables | 11 | 3 | 495 | 14 | 2 | 895 | 14 | 3 | 640 | 11 | 2 | | 302 |
| Services | 8 | 3 | 2,284 | 9 | 2 | 3,519 | 4 | 2 | 9,511 | 6 | 2 | | 729 |
| Others | 14 | 3 | 453 | 15 | 4 | 445 | 12 | 3 | 1,088 | 9 | 3 | | 779 |
| **T2DM+Stroke or TIA+Heart Failure** | **12** | **32** | **7,098** | **14** | **45** | **9,580** | **13** | **43** | **15,465** | **15** | **47** | | **24,054** |
| Medication | 3 | 10 | 2,273 | 3 | 11 | 3,370 | 3 | 14 | 3,557 | 3 | 14 | | 7,951 |
| Procedure | 3 | 9 | 2,824 | 3 | 10 | 3,610 | 3 | 10 | 6,829 | 3 | 12 | | 10,044 |
| Consultation | 3 | 10 | 788 | 3 | 12 | 784 | 3 | 14 | 1,018 | 3 | 15 | | 1,478 |
| Consumables | 1 | 1 | 1,000 | 2 | 1 | 162 | 2 | 1 | 523 | 3 | 2 | | 680 |
| Services |  |  |  | 1 | 7 | 633 | 1 | 2 | 3,283 | 2 | 3 | | 3,331 |
| Others | 2 | 2 | 213 | 2 | 4 | 1,021 | 1 | 1 | 255 | 1 | 1 | | 570 |
| Abbreviations: CVD=Cardiovascular disease, HCRU=Healthcare cost utilization, N=Number of patients, T2DM=Type 2 diabetes mellitus, TIA=Transient ischemic attack | | | | | | | | | | | |  |  |
