# Supplementary material for: Cost of cardiovascular diseases and renal complications in people with type 2 diabetes mellitus in the Kingdom of Saudi Arabia: A retrospective analysis of claims database
Source: PLoS One. 2022 Oct 20;17(10):e0273836. doi: 10.1371/journal.pone.0273836 (PMC9584438; doi:10.1371/journal.pone.0273836)
Supplement: S9 Table — (DOCX) [file pone.0273836.s009.docx]

### S9 Table: Comparison of pre-index and post-index all-cause cost for various activities (Payer 2, Cohort 1)

|  |  | | | | | |
| --- | --- | --- | --- | --- | --- | --- |
|  | **Pre-Index 1 Yr** | | | **Post-Index 1 Yr** | | |
| **Payer 2** | **All-Cause** | | | **All-Cause** | | |
| **Cohort 1** | **N** | **HCRU** | **Cost** | **N** | **HCRU** | **Cost** |
| **T2DM With One CVD** | | | | | | |
| T2DM+Angina | | | | | | |
| Medication | 133 | 12 | 3,441 | 133 | 13 | 3,885 |
| Procedure | 129 | 7 | 3,853 | 131 | 7 | 5,471 |
| Consultation | 132 | 13 | 1,168 | 130 | 13 | 1,117 |
| Consumables | 42 | 1 | 986 | 36 | 1 | 847 |
| Services | 102 | 4 | 844 | 91 | 4 | 1,992 |
| Others | 2 | 2 | 380 |  |  |  |
| T2DM+Atrial fibrillation | | | | | | |
| Medication | 27 | 14 | 7,199 | 26 | 15 | 8,570 |
| Procedure | 26 | 9 | 17,982 | 27 | 9 | 12,647 |
| Consultation | 26 | 15 | 2,142 | 27 | 15 | 2,120 |
| Consumables | 12 | 2 | 814 | 11 | 1 | 384 |
| Services | 22 | 3 | 4,123 | 20 | 5 | 4,932 |
| Others |  |  |  |  |  |  |
| T2DM+Chronic renal failure | | | | | | |
| Medication | 107 | 14 | 7,785 | 106 | 16 | 17,312 |
| Procedure | 106 | 9 | 11,887 | 107 | 11 | 19,840 |
| Consultation | 107 | 15 | 1,832 | 106 | 15 | 2,177 |
| Consumables | 34 | 1 | 410 | 40 | 2 | 1,600 |
| Services | 72 | 4 | 4,984 | 82 | 5 | 9,252 |
| Others | 2 | 3 | 728 | 1 | 1 | 177 |
| T2DM+Coronary Arterial Revascularization | | | | | | |
| Medication | 2 | 13 | 4,265 | 2 | 7 | 2,400 |
| Procedure | 2 | 9 | 28,753 | 2 | 3 | 440 |
| Consultation | 2 | 13 | 387 | 2 | 7 | 200 |
| Consumables |  |  |  | 1 | 2 | 980 |
| Services | 2 | 2 | 1,435 |  |  |  |
| Others |  |  |  |  |  |  |
| T2DM+Coronary Artery Disease | | | | | | |
| Medication | 780 | 14 | 5,967 | 778 | 14 | 5,877 |
| Procedure | 749 | 7 | 8,344 | 742 | 7 | 9,954 |
| Consultation | 774 | 15 | 1,529 | 771 | 14 | 1,442 |
| Consumables | 244 | 1 | 754 | 257 | 1 | 920 |
| Services | 536 | 3 | 2,387 | 522 | 3 | 3,025 |
| Others | 34 | 2 | 390 | 12 | 2 | 272 |
| T2DM+Dysrhythmia | | | | | | |
| Medication | 14 | 10 | 2,324 | 14 | 10 | 2,078 |
| Procedure | 14 | 6 | 2,975 | 13 | 8 | 8,294 |
| Consultation | 13 | 12 | 942 | 13 | 12 | 928 |
| Consumables | 7 | 2 | 509 | 3 | 1 | 356 |
| Services | 9 | 3 | 658 | 10 | 2 | 382 |
| Others | 1 | 1 | 15 | 1 | 1 | 30 |
| T2DM+Heart Failure | | | | | | |
| Medication | 42 | 13 | 10,050 | 40 | 13 | 13,162 |
| Procedure | 40 | 7 | 8,215 | 40 | 7 | 9,584 |
| Consultation | 42 | 14 | 1,543 | 40 | 13 | 1,961 |
| Consumables | 9 | 1 | 538 | 16 | 1 | 794 |
| Services | 25 | 4 | 1,324 | 29 | 4 | 9,920 |
| Others | 1 | 2 | 200 | 3 | 2 | 1,168 |
| T2DM+Myocardial infarction1 | | | | | | |
| Medication | 24 | 13 | 3,933 | 24 | 11 | 3,501 |
| Procedure | 23 | 6 | 7,730 | 23 | 6 | 10,342 |
| Consultation | 24 | 14 | 1,146 | 24 | 11 | 1,037 |
| Consumables | 8 | 1 | 466 | 6 | 2 | 194 |
| Services | 20 | 5 | 1,422 | 19 | 3 | 2,442 |
| Others | 1 | 1 | 16 | 2 | 1 | 94 |
| T2DM+Other Cardiovascular Disease | | | | | | |
| Medication | 13 | 12 | 3,151 | 13 | 12 | 3,010 |
| Procedure | 13 | 7 | 3,588 | 11 | 8 | 10,206 |
| Consultation | 13 | 13 | 1,745 | 12 | 15 | 1,509 |
| Consumables | 6 | 1 | 492 | 7 | 2 | 418 |
| Services | 8 | 3 | 862 | 9 | 3 | 1,087 |
| Others | 1 | 1 | 225 |  |  |  |
| T2DM+Periphery vascular disease | | | | | | |
| Medication | 11 | 12 | 4,112 | 10 | 10 | 7,381 |
| Procedure | 10 | 6 | 8,331 | 11 | 4 | 37,919 |
| Consultation | 11 | 13 | 1,237 | 10 | 10 | 1,736 |
| Consumables | 4 | 1 | 297 | 2 | 2 | 57,585 |
| Services | 10 | 4 | 883 | 9 | 4 | 12,328 |
| Others |  |  |  |  |  |  |
| T2DM+Stroke or TIA | 764 | 40 | 22,734 | 766 | 41 | 30,245 |
| Medication | 188 | 13 | 4,469 | 188 | 13 | 5,447 |
| Procedure | 182 | 7 | 10,709 | 183 | 7 | 9,231 |
| Consultation | 186 | 14 | 1,710 | 187 | 14 | 2,890 |
| Consumables | 58 | 1 | 2,030 | 62 | 2 | 1,597 |
| Services | 142 | 3 | 3,662 | 144 | 3 | 10,920 |
| Others | 8 | 1 | 155 | 2 | 2 | 159 |
| **T2DM With Multiple CVD** | | | | | | |
| T2DM+Coronary Artery Disease+Angina | | | | | | |
| Medication | 81 | 13 | 4,543 | 82 | 16 | 6,263 |
| Procedure | 79 | 6 | 5,494 | 82 | 8 | 19,092 |
| Consultation | 80 | 14 | 1,055 | 82 | 16 | 1,647 |
| Consumables | 16 | 1 | 408 | 30 | 1 | 1,146 |
| Services | 57 | 3 | 1,776 | 60 | 4 | 5,317 |
| Others | 3 | 2 | 460 | 3 | 2 | 241 |
| T2DM+Coronary Artery Disease+Atrial fibrillation | | | | | | |
| Medication | 20 | 13 | 6,341 | 20 | 16 | 7,923 |
| Procedure | 19 | 7 | 4,997 | 20 | 9 | 12,592 |
| Consultation | 20 | 16 | 1,487 | 20 | 16 | 1,772 |
| Consumables | 6 | 1 | 1,548 | 9 | 1 | 304 |
| Services | 13 | 3 | 2,347 | 18 | 2 | 5,918 |
| Others |  |  |  |  |  |  |
| T2DM+Coronary Artery Disease+Chronic renal failure | | | | | | |
| Medication | 18 | 13 | 11,296 | 18 | 20 | 25,583 |
| Procedure | 18 | 8 | 14,797 | 17 | 15 | 40,571 |
| Consultation | 18 | 15 | 1,802 | 18 | 18 | 2,865 |
| Consumables | 6 | 2 | 560 | 9 | 1 | 921 |
| Services | 13 | 3 | 1,328 | 16 | 5 | 10,011 |
| Others |  |  |  | 1 | 1 | 8 |
| T2DM+Heart Failure+Coronary Artery Disease | | | | | | |
| Medication | 34 | 14 | 6,026 | 34 | 16 | 11,948 |
| Procedure | 33 | 9 | 11,972 | 33 | 11 | 38,940 |
| Consultation | 34 | 15 | 2,202 | 33 | 18 | 4,169 |
| Consumables | 10 | 2 | 684 | 12 | 2 | 6,851 |
| Services | 25 | 4 | 2,918 | 29 | 6 | 20,244 |
| Others | 2 | 2 | 947 | 2 | 1 | 80 |
| T2DM+Myocardial infarction+Coronary Artery Disease | | | | | | |
| Medication | 36 | 12 | 4,683 | 37 | 15 | 7,659 |
| Procedure | 35 | 6 | 5,406 | 37 | 7 | 35,692 |
| Consultation | 36 | 13 | 1,153 | 37 | 15 | 1,593 |
| Consumables | 4 | 2 | 734 | 18 | 2 | 1,463 |
| Services | 24 | 4 | 2,877 | 32 | 4 | 8,659 |
| Others | 3 | 2 | 408 | 1 | 1 | 20 |
| T2DM+Myocardial infarction+Coronary Artery Disease+Angina | | | | | | |
| Medication | 10 | 15 | 5,796 | 11 | 18 | 8,721 |
| Procedure | 9 | 12 | 10,546 | 11 | 11 | 43,933 |
| Consultation | 11 | 17 | 1,361 | 11 | 22 | 2,198 |
| Consumables | 4 | 2 | 687 | 5 | 1 | 542 |
| Services | 9 | 5 | 3,513 | 11 | 4 | 9,556 |
| Others | 1 | 1 | 250 | 1 | 1 | 5 |
| T2DM+Stroke or TIA+Coronary Artery Disease | | | | | | |
| Medication | 41 | 14 | 6,307 | 41 | 17 | 8,264 |
| Procedure | 40 | 8 | 11,773 | 40 | 9 | 21,745 |
| Consultation | 41 | 16 | 3,161 | 40 | 19 | 3,669 |
| Consumables | 15 | 2 | 1,949 | 16 | 2 | 2,463 |
| Services | 33 | 5 | 6,503 | 38 | 4 | 6,781 |
| Others | 2 | 4 | 387 | 3 | 1 | 240 |

Abbreviations: CVD=Cardiovascular disease, HCRU=Healthcare cost utilization, N=Number of patients, T2DM=Type 2 diabetes mellitus, CVD=Cardiovascular disease, T2DM=Type 2 diabetes mellitus, TIA=Transient ischemic attack
