# Supplementary material for: Cost of cardiovascular diseases and renal complications in people with type 2 diabetes mellitus in the Kingdom of Saudi Arabia: A retrospective analysis of claims database
Source: PLoS One. 2022 Oct 20;17(10):e0273836. doi: 10.1371/journal.pone.0273836 (PMC9584438; doi:10.1371/journal.pone.0273836)
Supplement: S10 Table — (DOCX) [file pone.0273836.s010.docx]

### S10 Table: : Comparison of pre-index and post-index all-cause cost for various activities (Payer 2, Cohort 2)

|  | **Pre-Index 1 Yr** | | | **Post-Index 1 Yr** | | | **Post-Index 2 Yr** | | |
| --- | --- | --- | --- | --- | --- | --- | --- | --- | --- |
| **Payer 2** | **All-Cause** | | | **All-Cause** | | | **All-Cause** | | |
| **Cohort 2** | **N** | **HCRU** | **Cost** | **N** | **HCRU** | **Cost** | **N** | **HCRU** | **Cost** |
| **T2DM With One CVD7** | | | | | | | | | |
| T2DM+Angina | | | | | | | | | |
| Medication | 41 | 11 | 2,748 | 41 | 15 | 4,591 | 40 | 12 | 3,164 |
| Procedure | 41 | 7 | 4,261 | 41 | 8 | 3,939 | 39 | 7 | 2,416 |
| Consultation | 41 | 12 | 976 | 41 | 15 | 972 | 40 | 11 | 823 |
| Consumables | 13 | 1 | 557 | 14 | 1 | 544 | 11 | 1 | 533 |
| Services | 30 | 2 | 1,333 | 32 | 3 | 814 | 27 | 3 | 871 |
| Others | 1 | 2 | 740 |  |  |  |  |  |  |
| T2DM+Atrial fibrillation | | | | | | | | | |
| Medication | 6 | 12 | 8,735 | 6 | 21 | 15,981 | 6 | 19 | 10,335 |
| Procedure | 5 | 10 | 32,839 | 6 | 13 | 8,634 | 5 | 11 | 18,619 |
| Consultation | 5 | 15 | 1,728 | 6 | 22 | 2,383 | 6 | 16 | 1,174 |
| Consumables | 1 | 2 | 100 | 2 | 2 | 340 | 1 | 1 | 400 |
| Services | 5 | 3 | 4,785 | 5 | 6 | 1,888 | 5 | 6 | 2,645 |
| Others |  |  |  |  |  |  |  |  |  |
| T2DM+Chronic renal failure | | | | | | | | | |
| Medication | 29 | 15 | 6,288 | 29 | 21 | 36,574 | 29 | 13 | 13,814 |
| Procedure | 29 | 11 | 15,294 | 29 | 15 | 28,157 | 29 | 10 | 26,033 |
| Consultation | 29 | 16 | 2,158 | 29 | 18 | 2,283 | 28 | 13 | 1,976 |
| Consumables | 11 | 2 | 408 | 12 | 1 | 705 | 7 | 2 | 343 |
| Services | 22 | 4 | 3,561 | 24 | 6 | 8,104 | 18 | 7 | 14,403 |
| Others |  |  |  |  |  |  |  |  |  |
| T2DM+Coronary Artery Disease | | | | | | | | | |
| Medication | 246 | 14 | 5,994 | 246 | 16 | 6,788 | 243 | 13 | 4,574 |
| Procedure | 234 | 7 | 6,110 | 237 | 8 | 10,190 | 232 | 6 | 4,909 |
| Consultation | 243 | 15 | 1,267 | 243 | 17 | 1,591 | 238 | 13 | 1,059 |
| Consumables | 77 | 1 | 503 | 91 | 2 | 690 | 77 | 2 | 511 |
| Services | 177 | 3 | 1,554 | 174 | 4 | 2,748 | 150 | 3 | 1,265 |
| Others | 14 | 2 | 264 | 5 | 2 | 379 | 3 | 1 | 450 |
| T2DM+Dysrhythmia | | | | | | | | | |
| Medication | 3 | 7 | 1,539 | 3 | 8 | 1,705 | 3 | 8 | 2,172 |
| Procedure | 3 | 4 | 2,714 | 3 | 7 | 6,487 | 3 | 6 | 3,680 |
| Consultation | 3 | 9 | 1,213 | 3 | 11 | 1,700 | 3 | 13 | 1,282 |
| Consumables | 2 | 2 | 363 | 1 | 1 | 585 | 2 | 1 | 493 |
| Services | 1 | 1 | 35 | 1 | 1 | 556 | 2 | 1 | 1,433 |
| Others |  |  |  | 1 | 1 | 30 |  |  |  |
| T2DM+Heart Failure | | | | | | | | | |
| Medication | 16 | 14 | 18,180 | 16 | 15 | 19,646 | 16 | 12 | 16,026 |
| Procedure | 15 | 8 | 5,762 | 15 | 8 | 6,154 | 15 | 5 | 8,653 |
| Consultation | 16 | 15 | 1,092 | 16 | 16 | 1,732 | 16 | 12 | 1,331 |
| Consumables | 4 | 1 | 301 | 7 | 1 | 375 | 8 | 1 | 364 |
| Services | 11 | 5 | 824 | 10 | 4 | 1,201 | 7 | 3 | 5,257 |
| Others |  |  |  | 1 | 2 | 220 |  |  |  |
| T2DM+Myocardial infarction | | | | | | | | | |
| Medication | 5 | 11 | 2,534 | 5 | 7 | 2,693 | 5 | 8 | 2,118 |
| Procedure | 4 | 4 | 14,486 | 5 | 6 | 7,387 | 4 | 5 | 503 |
| Consultation | 5 | 11 | 572 | 5 | 7 | 585 | 5 | 8 | 320 |
| Consumables | 1 | 1 | 472 |  |  |  |  |  |  |
| Services | 3 | 2 | 2,794 | 4 | 2 | 4,026 | 2 | 2 | 835 |
| Others | 1 | 1 | 16 |  |  |  |  |  |  |
| T2DM+Other Cardiovascular Disease | | | | | | | | | |
| Medication | 5 | 14 | 3,602 | 5 | 18 | 5,167 | 5 | 8 | 1,940 |
| Procedure | 5 | 8 | 3,395 | 5 | 9 | 4,793 | 4 | 3 | 691 |
| Consultation | 5 | 13 | 655 | 5 | 21 | 1,247 | 4 | 16 | 1,028 |
| Consumables | 2 | 1 | 110 | 1 | 3 | 405 | 2 | 2 | 495 |
| Services | 3 | 3 | 198 | 5 | 4 | 1,814 | 5 | 2 | 108 |
| Others |  |  |  |  |  |  |  |  |  |
| T2DM+Periphery vascular disease | | | | | | | | | |
| Medication | 1 | 10 | 4,200 | 1 | 6 | 3,711 | 1 | 10 | 6,731 |
| Procedure | 1 | 10 | 3,059 | 1 | 9 | 2,117 | 1 | 10 | 3,360 |
| Consultation | 1 | 12 | 1,460 | 1 | 11 | 930 | 1 | 13 | 1,850 |
| Consumables | 1 | 2 | 72 |  |  |  | 1 | 3 | 479 |
| Services | 1 | 2 | 22 |  |  |  | 1 | 1 | 713 |
| Others |  |  |  |  |  |  |  |  |  |
| T2DM+Stroke or TIA | | | | | | | | | |
| Medication | 58 | 12 | 3,922 | 58 | 14 | 5,317 | 58 | 12 | 4,341 |
| Procedure | 55 | 7 | 7,393 | 57 | 7 | 6,373 | 57 | 6 | 4,089 |
| Consultation | 57 | 14 | 1,439 | 57 | 16 | 2,294 | 57 | 12 | 1,517 |
| Consumables | 19 | 1 | 441 | 23 | 2 | 441 | 16 | 1 | 508 |
| Services | 44 | 3 | 2,161 | 46 | 3 | 2,484 | 37 | 3 | 1,021 |
| Others | 4 | 1 | 218 |  |  |  | 1 | 1 | 115 |
| **T2DM With Multiple CVD** | | | | | | | | | |
| T2DM+Coronary Artery Disease+Angina | 122 | 41 | 14,197 | 128 | 51 | 34,281 | 124 | 42 | 24,997 |
| Medication | 32 | 14 | 4,942 | 32 | 18 | 7,189 | 32 | 16 | 6,733 |
| Procedure | 31 | 6 | 5,909 | 32 | 8 | 17,732 | 31 | 7 | 14,144 |
| Consultation | 31 | 15 | 982 | 32 | 18 | 1,616 | 32 | 15 | 1,275 |
| Consumables | 4 | 2 | 244 | 10 | 1 | 553 | 8 | 2 | 567 |
| Services | 23 | 3 | 1,014 | 20 | 4 | 6,832 | 21 | 2 | 2,278 |
| Others | 1 | 1 | 1,107 | 2 | 2 | 359 |  |  |  |
| T2DM+Coronary Artery Disease+Atrial fibrillation | | | | | | | | | |
| Medication | 7 | 12 | 6,481 | 7 | 16 | 9,307 | 7 | 13 | 10,442 |
| Procedure | 6 | 5 | 3,655 | 7 | 9 | 16,724 | 7 | 5 | 5,683 |
| Consultation | 7 | 15 | 1,331 | 7 | 18 | 2,148 | 7 | 13 | 1,487 |
| Consumables | 2 | 2 | 545 | 4 | 2 | 227 | 3 | 1 | 460 |
| Services | 4 | 2 | 571 | 7 | 3 | 11,231 | 5 | 3 | 2,027 |
| Others |  |  |  |  |  |  |  |  |  |
| T2DM+Coronary Artery Disease+Chronic renal failure | 19 | 48 | 51,440 | 25 | 91 | 122,391 | 24 | 86 | 157,005 |
| Medication | 5 | 15 | 24,228 | 5 | 30 | 66,272 | 5 | 29 | 75,298 |
| Procedure | 5 | 11 | 23,691 | 5 | 22 | 48,016 | 5 | 20 | 75,326 |
| Consultation | 5 | 15 | 1,748 | 5 | 30 | 3,818 | 5 | 30 | 3,511 |
| Consumables | 2 | 3 | 813 | 4 | 2 | 330 | 4 | 2 | 1,157 |
| Services | 2 | 5 | 961 | 5 | 7 | 3,947 | 5 | 5 | 1,712 |
| Others |  |  |  | 1 | 1 | 8 |  |  |  |
| T2DM+Heart Failure+Coronary Artery Disease78 | | | | | | | | | |
| Medication | 13 | 16 | 5,224 | 13 | 18 | 8,867 | 12 | 12 | 6,287 |
| Procedure | 13 | 9 | 6,997 | 13 | 11 | 19,324 | 12 | 8 | 9,571 |
| Consultation | 13 | 17 | 1,376 | 13 | 20 | 2,457 | 12 | 11 | 1,628 |
| Consumables | 5 | 1 | 472 | 4 | 2 | 5,205 | 3 | 1 | 488 |
| Services | 10 | 4 | 1,113 | 11 | 6 | 11,929 | 10 | 4 | 5,504 |
| Others |  |  |  |  |  |  |  |  |  |
| T2DM+Myocardial infarction+Coronary Artery Disease | | | | | | | | | |
| Medication | 18 | 13 | 4,656 | 18 | 16 | 6,873 | 18 | 12 | 4,319 |
| Procedure | 17 | 6 | 4,397 | 18 | 8 | 28,209 | 17 | 5 | 6,806 |
| Consultation | 18 | 14 | 1,215 | 18 | 16 | 1,479 | 18 | 11 | 799 |
| Consumables | 2 | 2 | 255 | 6 | 2 | 354 | 5 | 1 | 376 |
| Services | 13 | 3 | 3,606 | 15 | 4 | 6,567 | 10 | 4 | 1,749 |
| Others | 3 | 2 | 408 |  |  |  |  |  |  |
| T2DM+Stroke or TIA+Angina | | | | | | | | | |
| Medication | 3 | 8 | 1,074 | 3 | 10 | 1,608 | 3 | 4 | 332 |
| Procedure | 3 | 5 | 1,245 | 3 | 10 | 8,080 | 3 | 4 | 9,467 |
| Consultation | 3 | 9 | 629 | 3 | 16 | 3,341 | 3 | 4 | 441 |
| Consumables |  |  |  | 2 | 1 | 143 | 1 | 1 | 400 |
| Services |  |  |  | 3 | 5 | 576 | 1 | 2 | 174 |
| Others |  |  |  |  |  |  | 1 | 2 | 613 |
| T2DM+Stroke or TIA+Coronary Artery Disease | | | | | | | | | |
| Medication | 21 | 16 | 6,430 | 21 | 18 | 9,821 | 21 | 16 | 9,434 |
| Procedure | 20 | 10 | 10,526 | 20 | 10 | 12,419 | 19 | 9 | 12,356 |
| Consultation | 21 | 20 | 3,402 | 20 | 22 | 3,728 | 21 | 16 | 2,230 |
| Consumables | 10 | 1 | 844 | 10 | 2 | 1,224 | 14 | 1 | 544 |
| Services | 17 | 5 | 2,978 | 19 | 4 | 6,236 | 18 | 3 | 1,891 |
| Others | 1 | 2 | 96 | 2 | 2 | 359 | 1 | 1 | 0 |

Abbreviations: CVD=Cardiovascular disease, HCRU=Healthcare cost utilization, N=Number of patients, T2DM=Type 2 diabetes mellitus, TIA=Transient ischemic attack
