# Supplementary material for: Cost of cardiovascular diseases and renal complications in people with type 2 diabetes mellitus in the Kingdom of Saudi Arabia: A retrospective analysis of claims database
Source: PLoS One. 2022 Oct 20;17(10):e0273836. doi: 10.1371/journal.pone.0273836 (PMC9584438; doi:10.1371/journal.pone.0273836)
Supplement: S11 Table — (DOCX) [file pone.0273836.s011.docx]

### S11 Table: Comparison of pre-index and post-index all-cause cost for various activities (Payer 2, Cohort 3)

|  | **Pre-Index 1 Yr** | | | **Post-Index 1 Yr** | | | **Post-Index 2 Yr** | | | **Post-Index 3 Yr** | | |
| --- | --- | --- | --- | --- | --- | --- | --- | --- | --- | --- | --- | --- |
|  | **All-Cause** |  |  | **All-Cause** |  |  | **All-Cause** |  |  | **All-Cause** |  |  |
|  | **N** | **HCRU** | **Cost** | **N** | **HCRU** | **Cost** | **N** | **HCRU** | **Cost** | **N** | **HCRU** | **Cost** |
| **T2DM WITH ONE CVD** | | | | | | | | | | | | |
| **T2DM+Angina** | **23** | **37** | **12,364** | **22** | **31** | **8,604** | **21** | **34** | **11,560** | **23** | **28** | **7,751** |
| Medication | 5 | 12 | 3,377 | 5 | 11 | 3,822 | 5 | 10 | 2,558 | 5 | 9 | 2,652 |
| Procedure | 5 | 9 | 5,557 | 5 | 6 | 2,846 | 4 | 9 | 5,341 | 5 | 5 | 2,470 |
| Consultation | 5 | 12 | 1,302 | 5 | 10 | 1,066 | 5 | 10 | 1,363 | 5 | 9 | 1,128 |
| Consumables | 3 | 1 | 262 | 3 | 1 | 533 | 3 | 2 | 631 | 3 | 2 | 668 |
| Services | 5 | 3 | 1,866 | 4 | 2 | 336 | 4 | 3 | 1,667 | 4 | 2 | 762 |
| Others |  |  |  |  |  |  |  |  |  | 1 | 1 | 70 |
| **T2DM+Atrial fibrillation** | **1** | **8** | **1,056** | **3** | **12** | **1,385** | **4** | **31** | **7,280** | **3** | **7** | **2,311** |
| Medication | 1 | 8 | 1,056 | 1 | 5 | 905 | 1 | 17 | 4,664 | 1 | 4 | 2,191 |
| Procedure |  |  |  | 1 | 3 | 260 | 1 | 3 | 800 | 1 | 1 | 0 |
| Consultation |  |  |  | 1 | 4 | 220 | 1 | 10 | 570 | 1 | 2 | 120 |
| Consumables |  |  |  |  |  |  |  |  |  |  |  |  |
| Services |  |  |  |  |  |  | 1 | 1 | 1,246 |  |  |  |
| Others |  |  |  |  |  |  |  |  |  |  |  |  |
| **T2DM+Chronic renal failure** | **15** | **41** | **23,411** | **18** | **54** | **128,472** | **14** | **36** | **128,357** | **15** | **40** | **114,193** |
| Medication | 4 | 12 | 10,783 | 4 | 20 | 85,458 | 4 | 12 | 45,747 | 4 | 14 | 35,851 |
| Procedure | 4 | 10 | 9,071 | 4 | 14 | 33,016 | 4 | 8 | 61,471 | 4 | 13 | 65,917 |
| Consultation | 4 | 13 | 1,798 | 4 | 14 | 2,010 | 4 | 10 | 2,020 | 4 | 9 | 2,647 |
| Consumables | 1 | 3 | 760 | 2 | 1 | 352 |  |  |  |  |  |  |
| Services | 2 | 3 | 999 | 4 | 6 | 7,635 | 2 | 6 | 19,119 | 3 | 4 | 9,779 |
| Others |  |  |  |  |  |  |  |  |  |  |  |  |
| **T2DM+Coronary Artery Disease** | **94** | **38** | **12,525** | **91** | **51** | **13,645** | **92** | **51** | **18,373** | **86** | **32** | **7,990** |
| Medication | 23 | 13 | 3,195 | 23 | 17 | 5,200 | 22 | 16 | 4,885 | 22 | 11 | 3,439 |
| Procedure | 22 | 5 | 6,884 | 22 | 8 | 4,499 | 21 | 7 | 7,720 | 20 | 6 | 2,575 |
| Consultation | 23 | 15 | 1,139 | 23 | 19 | 1,469 | 22 | 20 | 2,283 | 23 | 12 | 1,176 |
| Consumables | 6 | 1 | 490 | 7 | 1 | 492 | 9 | 1 | 488 | 5 | 1 | 461 |
| Services | 17 | 3 | 746 | 16 | 5 | 1,985 | 17 | 5 | 1,736 | 16 | 2 | 337 |
| Others | 3 | 1 | 70 |  |  |  | 1 | 2 | 1,260 |  |  |  |
| **T2DM+Other Cardiovascular Disease** | **4** | **17** | **6,472** | **4** | **16** | **5,166** | **5** | **24** | **6,331** | **3** | **9** | **5,330** |
| Medication | 1 | 4 | 1,447 | 1 | 7 | 3,241 | 1 | 7 | 3,921 | 1 | 3 | 4,165 |
| Procedure | 1 | 4 | 4,430 | 1 | 2 | 1,080 | 1 | 5 | 500 | 1 | 3 | 805 |
| Consultation | 1 | 6 | 520 | 1 | 6 | 660 | 1 | 8 | 810 | 1 | 3 | 360 |
| Consumables |  |  |  |  |  |  | 1 | 2 | 930 |  |  |  |
| Services | 1 | 3 | 75 | 1 | 1 | 185 | 1 | 2 | 170 |  |  |  |
| Others |  |  |  |  |  |  |  |  |  |  |  |  |
| **T2DM+Stroke or TIA** | **30** | **47** | **24,699** | **30** | **43** | **7,553** | **29** | **45** | **9,814** | **27** | **28** | **9,647** |
| Medication | 7 | 15 | 4,353 | 7 | 17 | 4,474 | 7 | 16 | 4,879 | 7 | 11 | 4,267 |
| Procedure | 6 | 9 | 17,806 | 6 | 7 | 1,793 | 6 | 8 | 3,510 | 6 | 4 | 4,198 |
| Consultation | 7 | 17 | 1,362 | 7 | 16 | 811 | 7 | 17 | 744 | 7 | 10 | 424 |
| Consumables | 3 | 1 | 383 | 5 | 1 | 317 | 3 | 1 | 433 | 3 | 1 | 569 |
| Services | 7 | 5 | 795 | 5 | 2 | 159 | 6 | 3 | 248 | 4 | 2 | 190 |
| Others |  |  |  |  |  |  |  |  |  |  |  |  |
| **T2DM WITH MULTIPLE CVD** | | | | | | | | | | | | |
| **T2DM+Coronary Arterial Revascularization+Coronary Artery Disease+Atrial fibrillation+Angina** | **4** | **25** | **6,315** | **4** | **88** | **61,170** | **5** | **63** | **17,909** | **5** | **49** | **21,446** |
| Medication | 1 | 9 | 4,757 | 1 | 29 | 11,371 | 1 | 20 | 11,448 | 1 | 18 | 8,124 |
| Procedure | 1 | 2 | 578 | 1 | 24 | 37,977 | 1 | 15 | 4,234 | 1 | 12 | 10,236 |
| Consultation | 1 | 11 | 795 | 1 | 27 | 2,415 | 1 | 21 | 1,798 | 1 | 14 | 1,315 |
| Consumables |  |  |  |  |  |  | 1 | 1 | 84 | 1 | 1 | 175 |
| Services | 1 | 3 | 185 | 1 | 8 | 9,406 | 1 | 6 | 345 | 1 | 4 | 1,596 |
| Others |  |  |  |  |  |  |  |  |  |  |  |  |
| **T2DM+Coronary Artery Disease+Angina** | **16** | **51** | **13,311** | **16** | **64** | **53,240** | **18** | **47** | **33,174** | **18** | **37** | **6,921** |
| Medication | 4 | 17 | 4,015 | 4 | 23 | 8,751 | 4 | 17 | 6,433 | 4 | 11 | 1,089 |
| Procedure | 4 | 11 | 7,514 | 4 | 11 | 32,099 | 4 | 9 | 17,138 | 4 | 9 | 5,001 |
| Consultation | 4 | 19 | 1,438 | 4 | 24 | 3,253 | 4 | 16 | 2,439 | 4 | 11 | 468 |
| Consumables |  |  |  |  |  |  | 2 | 1 | 450 | 2 | 2 | 182 |
| Services | 4 | 3 | 344 | 4 | 7 | 9,137 | 4 | 4 | 6,715 | 4 | 5 | 182 |
| Others |  |  |  |  |  |  |  |  |  |  |  |  |
| **T2DM+Coronary Artery Disease+Chronic renal failure** | **9** | **70** | **57,966** | **11** | **137** | **102,270** | **10** | **133** | **84,260** | **9** | **66** | **42,636** |
| Medication | 2 | 19 | 8,767 | 2 | 43 | 26,102 | 2 | 43 | 27,485 | 2 | 18 | 25,250 |
| Procedure | 2 | 15 | 44,125 | 2 | 31 | 65,186 | 2 | 26 | 48,740 | 2 | 12 | 13,095 |
| Consultation | 2 | 25 | 2,484 | 2 | 49 | 5,563 | 2 | 54 | 5,428 | 2 | 24 | 2,771 |
| Consumables | 2 | 3 | 813 | 2 | 2 | 545 | 2 | 3 | 965 | 1 | 4 | 368 |
| Services | 1 | 8 | 1,778 | 2 | 12 | 4,865 | 2 | 8 | 1,642 | 2 | 8 | 1,152 |
| Others |  |  |  | 1 | 1 | 8 |  |  |  |  |  |  |
| **T2DM+Heart Failure+Coronary Artery Disease** | **4** | **44** | **13,150** | **5** | **94** | **44,604** | **5** | **115** | **170,125** | **5** | **105** | **51,908** |
| Medication | 1 | 15 | 4,545 | 1 | 34 | 11,633 | 1 | 32 | 33,880 | 1 | 34 | 18,626 |
| Procedure | 1 | 8 | 5,209 | 1 | 14 | 11,353 | 1 | 28 | 79,268 | 1 | 24 | 20,273 |
| Consultation | 1 | 14 | 1,498 | 1 | 27 | 3,844 | 1 | 38 | 8,349 | 1 | 28 | 5,863 |
| Consumables |  |  |  | 1 | 1 | 1,068 | 1 | 1 | 313 | 1 | 3 | 946 |
| Services | 1 | 7 | 1,898 | 1 | 18 | 16,705 | 1 | 16 | 48,315 | 1 | 16 | 6,201 |
| Others |  |  |  |  |  |  |  |  |  |  |  |  |
| **T2DM+Myocardial infarction+Coronary Artery Disease** | **4** | **9** | **18,550** | **3** | **22** | **28,630** | **3** | **37** | **2,160** | **3** | **12** | **829** |
| Medication | 1 | 3 | 446 | 1 | 7 | 1,504 | 1 | 19 | 1,455 | 1 | 5 | 494 |
| Procedure | 1 | 2 | 145 | 1 | 6 | 26,534 | 1 | 6 | 465 | 1 | 2 | 235 |
| Consultation | 1 | 3 | 80 | 1 | 9 | 592 | 1 | 12 | 240 | 1 | 5 | 100 |
| Consumables |  |  |  |  |  |  |  |  |  |  |  |  |
| Services | 1 | 1 | 17,878 |  |  |  |  |  |  |  |  |  |
| Others |  |  |  |  |  |  |  |  |  |  |  |  |
| **T2DM+Other Cardiovascular Disease+Coronary Artery Disease** | **5** | **32** | **7,312** | **4** | **56** | **10,803** | **5** | **57** | **44,954** | **4** | **31** | **10,116** |
| Medication | 1 | 10 | 1,841 | 1 | 13 | 3,082 | 1 | 17 | 4,947 | 1 | 9 | 3,374 |
| Procedure | 1 | 7 | 2,637 | 1 | 10 | 4,513 | 1 | 16 | 34,697 | 1 | 7 | 4,088 |
| Consultation | 1 | 12 | 625 | 1 | 26 | 1,859 | 1 | 18 | 669 | 1 | 10 | 965 |
| Consumables | 1 | 1 | 479 |  |  |  | 1 | 2 | 979 |  |  |  |
| Services | 1 | 2 | 1,730 | 1 | 7 | 1,350 | 1 | 4 | 3,663 | 1 | 5 | 1,689 |
| Others |  |  |  |  |  |  |  |  |  |  |  |  |
| **T2DM+Stroke or TIA+Coronary Artery Disease** | **9** | **65** | **21,583** | **9** | **78** | **25,455** | **10** | **60** | **20,738** | **8** | **27** | **10,881** |
| Medication | 2 | 24 | 7,991 | 2 | 30 | 10,164 | 2 | 23 | 10,274 | 2 | 11 | 4,874 |
| Procedure | 2 | 9 | 8,685 | 2 | 9 | 8,169 | 2 | 11 | 6,561 | 2 | 5 | 3,674 |
| Consultation | 2 | 25 | 3,036 | 2 | 34 | 6,296 | 2 | 23 | 3,285 | 2 | 10 | 2,169 |
| Consumables | 1 | 1 | 484 | 1 | 2 | 400 | 2 | 1 | 433 |  |  |  |
| Services | 2 | 7 | 1,386 | 2 | 3 | 426 | 2 | 2 | 186 | 2 | 2 | 165 |
| Others |  |  |  |  |  |  |  |  |  |  |  |  |

Abbreviations: CVD=Cardiovascular disease, HCRU=Healthcare cost utilization, N=Number of patients, T2DM=Type 2 diabetes mellitus, TIA=Transient ischemic attack
