# Supplementary material for: Cost of cardiovascular diseases and renal complications in people with type 2 diabetes mellitus in the Kingdom of Saudi Arabia: A retrospective analysis of claims database
Source: PLoS One. 2022 Oct 20;17(10):e0273836. doi: 10.1371/journal.pone.0273836 (PMC9584438; doi:10.1371/journal.pone.0273836)
Supplement: S12 Table — (DOCX) [file pone.0273836.s012.docx]

### S12 Table: Comparison of pre-index and post-index all-cause cost for various activities (Payer 3, Cohort 1)

|  |  | | | | | |
| --- | --- | --- | --- | --- | --- | --- |
|  | **Pre-Index 1 Yr** | | | **Post-Index 1 Yr** | | |
| **Payer 3** | **All-Cause** | | | **All-Cause** | | |
| **Cohort 1** | **N** | **HCRU** | **Cost** | **N** | **HCRU** | **Cost** |
| **T2DM With One CVD** | | | | | | |
| Coronary Arterial Revascularization+T2DM | | | | | | |
| Medication | 34 | 10 | 10,200 | 34 | 11 | 14,638 |
| Procedure | 33 | 5 | 14,156 | 33 | 6 | 15,793 |
| Consultation | 34 | 11 | 2,329 | 34 | 12 | 2,774 |
| Consumables | 16 | 2 | 1,458 | 16 | 3 | 2,230 |
| Services | 10 | 2 | 5,456 | 9 | 2 | 7,021 |
| Others | 9 | 2 | 280 | 11 | 1 | 485 |
| T2DM+Angina | | | | | | |
| Medication | 455 | 13 | 5,658 | 458 | 13 | 6,868 |
| Procedure | 449 | 7 | 6,951 | 451 | 8 | 8,600 |
| Consultation | 457 | 13 | 2,210 | 458 | 14 | 2,363 |
| Consumables | 196 | 2 | 709 | 218 | 2 | 831 |
| Services | 178 | 3 | 3,046 | 200 | 3 | 4,154 |
| Others | 130 | 2 | 627 | 127 | 2 | 340 |
| T2DM+Atrial fibrillation | | | | | | |
| Medication | 62 | 13 | 9,217 | 62 | 17 | 19,837 |
| Procedure | 61 | 8 | 13,917 | 61 | 10 | 16,100 |
| Consultation | 62 | 14 | 2,947 | 62 | 17 | 3,690 |
| Consumables | 33 | 2 | 998 | 36 | 3 | 1,521 |
| Services | 34 | 3 | 4,545 | 32 | 3 | 8,018 |
| Others | 16 | 2 | 323 | 16 | 2 | 422 |
| T2DM+cardiac ischemia | | | | | | |
| Medication | 1 | 21 | 8,234 | 1 | 7 | 3,425 |
| Procedure | 1 | 12 | 26,990 | 1 | 3 | 3,621 |
| Consultation | 1 | 21 | 4,590 | 1 | 7 | 1,868 |
| Consumables | 1 | 1 | 1,229 |  |  |  |
| Services | 1 | 1 | 2,100 |  |  |  |
| Others | 1 | 1 | 5 |  |  |  |
| T2DM+Chronic renal failure | | | | | | |
| Medication | 233 | 13 | 10,089 | 233 | 15 | 12,756 |
| Procedure | 231 | 8 | 12,888 | 232 | 11 | 18,108 |
| Consultation | 233 | 14 | 2,962 | 233 | 16 | 3,553 |
| Consumables | 100 | 2 | 1,050 | 129 | 3 | 893 |
| Services | 121 | 3 | 8,157 | 137 | 4 | 11,855 |
| Others | 64 | 1 | 383 | 61 | 2 | 784 |
| T2DM+Coronary Artery Disease | | | | | | |
| Medication | 1,048 | 13 | 7,431 | 1,052 | 14 | 9,535 |
| Procedure | 1,006 | 7 | 8,016 | 1,009 | 7 | 9,263 |
| Consultation | 1,048 | 13 | 2,080 | 1,049 | 14 | 2,167 |
| Consumables | 426 | 2 | 1,004 | 419 | 2 | 940 |
| Services | 471 | 3 | 5,244 | 515 | 3 | 5,299 |
| Others | 275 | 2 | 479 | 249 | 2 | 542 |
| T2DM+Dysrhythmia | | | | | | |
| Medication | 58 | 15 | 7,048 | 58 | 17 | 8,875 |
| Procedure | 57 | 9 | 9,443 | 58 | 12 | 16,454 |
| Consultation | 58 | 17 | 2,446 | 58 | 18 | 3,506 |
| Consumables | 24 | 2 | 592 | 28 | 2 | 2,716 |
| Services | 28 | 4 | 5,095 | 25 | 7 | 23,598 |
| Others | 16 | 1 | 257 | 21 | 2 | 302 |
| T2DM+Heart Failure | | | | | | |
| Medication | 152 | 12 | 11,461 | 152 | 13 | 13,046 |
| Procedure | 147 | 7 | 10,889 | 149 | 8 | 13,289 |
| Consultation | 152 | 13 | 2,855 | 151 | 14 | 3,611 |
| Consumables | 74 | 2 | 1,512 | 68 | 3 | 1,809 |
| Services | 70 | 2 | 7,340 | 72 | 3 | 11,854 |
| Others | 57 | 2 | 283 | 43 | 2 | 426 |
| T2DM+Myocardial infarction | | | | | | |
| Medication | 53 | 14 | 5,651 | 53 | 11 | 5,414 |
| Procedure | 52 | 7 | 6,660 | 51 | 6 | 9,239 |
| Consultation | 53 | 14 | 1,800 | 52 | 12 | 1,872 |
| Consumables | 20 | 1 | 2,420 | 15 | 1 | 425 |
| Services | 27 | 2 | 6,537 | 27 | 3 | 4,424 |
| Others | 10 | 2 | 263 | 12 | 1 | 1,605 |
| T2DM+Other Cardiovascular Disease | | | | | | |
| Medication | 26 | 11 | 6,925 | 26 | 14 | 7,429 |
| Procedure | 25 | 8 | 7,486 | 26 | 9 | 15,264 |
| Consultation | 26 | 13 | 2,607 | 26 | 16 | 3,036 |
| Consumables | 14 | 2 | 748 | 16 | 2 | 6,008 |
| Services | 11 | 3 | 1,544 | 14 | 3 | 4,146 |
| Others | 12 | 1 | 468 | 7 | 1 | 629 |
| T2DM+Periphery vascular disease | | | | | | |
| Medication | 15 | 11 | 9,955 | 15 | 14 | 9,543 |
| Procedure | 13 | 8 | 6,546 | 14 | 9 | 20,531 |
| Consultation | 15 | 13 | 1,602 | 15 | 18 | 2,380 |
| Consumables | 5 | 2 | 766 | 5 | 2 | 691 |
| Services | 9 | 11 | 1,548 | 9 | 12 | 15,116 |
| Others |  |  |  | 5 | 1 | 176 |
| T2DM+Stroke or TIA | | | | | | |
| Medication | 522 | 13 | 8,046 | 524 | 15 | 10,171 |
| Procedure | 515 | 8 | 9,621 | 510 | 9 | 13,248 |
| Consultation | 523 | 14 | 3,021 | 524 | 16 | 4,089 |
| Consumables | 232 | 2 | 800 | 282 | 2 | 991 |
| Services | 229 | 3 | 6,365 | 267 | 3 | 11,294 |
| Others | 154 | 2 | 400 | 153 | 1 | 398 |
| **T2DM With Multiple CVD**065  2,439  526  568,731 | | | | | | |
| Coronary Arterial Revascularization+T2DM+Coronary Artery Disease | | | | | | |
| Medication | 53 | 12 | 8,606 | 53 | 16 | 10,711 |
| Procedure | 49 | 7 | 12,213 | 50 | 9 | 15,435 |
| Consultation | 53 | 13 | 1,820 | 53 | 18 | 2,972 |
| Consumables | 24 | 2 | 3,684 | 20 | 2 | 2,026 |
| Services | 27 | 2 | 1,860 | 29 | 3 | 4,195 |
| Others | 19 | 1 | 732 | 14 | 1 | 2,738 |
| Coronary Arterial Revascularization+T2DM+Coronary Artery Disease+Angina | | | | | | |
| Medication | 19 | 16 | 10,565 | 19 | 18 | 17,499 |
| Procedure | 19 | 10 | 14,928 | 19 | 13 | 36,526 |
| Consultation | 19 | 18 | 3,201 | 19 | 20 | 4,653 |
| Consumables | 13 | 2 | 1,469 | 13 | 2 | 4,284 |
| Services | 12 | 2 | 3,502 | 14 | 3 | 9,973 |
| Others | 7 | 1 | 178 | 9 | 4 | 1,528 |
| T2DM+Coronary Artery Disease+Angina | | | | | | |
| Medication | 190 | 14 | 8,049 | 190 | 17 | 11,368 |
| Procedure | 185 | 8 | 9,206 | 183 | 9 | 28,226 |
| Consultation | 190 | 14 | 2,178 | 190 | 17 | 2,961 |
| Consumables | 65 | 2 | 1,518 | 85 | 2 | 1,671 |
| Services | 98 | 3 | 5,556 | 135 | 4 | 7,520 |
| Others | 38 | 2 | 841 | 45 | 1 | 710 |
| T2DM+Coronary Artery Disease+Atrial fibrillation | | | | | | |
| Medication | 19 | 15 | 8,978 | 19 | 18 | 14,468 |
| Procedure | 19 | 10 | 9,358 | 18 | 10 | 13,491 |
| Consultation | 19 | 17 | 2,542 | 19 | 18 | 2,926 |
| Consumables | 9 | 2 | 2,271 | 9 | 2 | 762 |
| Services | 13 | 4 | 9,379 | 13 | 4 | 32,110 |
| Others | 6 | 3 | 443 | 6 | 3 | 848 |
| T2DM+Coronary Artery Disease+Chronic renal failure | | | | | | |
| Medication | 35 | 16 | 10,626 | 35 | 18 | 16,002 |
| Procedure | 34 | 8 | 24,274 | 34 | 10 | 18,575 |
| Consultation | 34 | 16 | 3,179 | 35 | 18 | 3,985 |
| Consumables | 12 | 2 | 2,669 | 19 | 2 | 813 |
| Services | 23 | 4 | 8,518 | 24 | 5 | 8,426 |
| Others | 9 | 2 | 1,239 | 11 | 1 | 484 |
| T2DM+Heart Failure+Coronary Artery Disease | | | | | | |
| Medication | 68 | 14 | 10,260 | 68 | 17 | 13,272 |
| Procedure | 66 | 8 | 11,435 | 68 | 9 | 22,994 |
| Consultation | 68 | 14 | 2,654 | 68 | 17 | 3,363 |
| Consumables | 37 | 2 | 1,289 | 38 | 2 | 4,143 |
| Services | 39 | 3 | 11,921 | 45 | 4 | 15,402 |
| Others | 17 | 1 | 313 | 23 | 2 | 420 |

Abbreviations: CVD=Cardiovascular disease, HCRU=Healthcare cost utilization, N=Number of patients, T2DM=Type 2 diabetes mellitus, TIA=Transient ischemic attack
