# Supplementary material for: Cost of cardiovascular diseases and renal complications in people with type 2 diabetes mellitus in the Kingdom of Saudi Arabia: A retrospective analysis of claims database
Source: PLoS One. 2022 Oct 20;17(10):e0273836. doi: 10.1371/journal.pone.0273836 (PMC9584438; doi:10.1371/journal.pone.0273836)
Supplement: S13 Table — (DOCX) [file pone.0273836.s013.docx]

### S13 Table: Comparison of pre-index and post-index all-cause cost for various activities (Payer 3, Cohort 2)

|  | **Pre-Index 1 Yr** | | | **Post-Index 1 Yr** | | | **Post-Index 2 Yr** | | |
| --- | --- | --- | --- | --- | --- | --- | --- | --- | --- |
| **Payer 3** | **All-Cause** | | | **All-Cause** | | | **All-Cause** | | |
| **Cohort 2** | **N** | **HCRU** | **Cost** | **N** | **HCRU** | **Cost** | **N** | **HCRU** | **Cost** |
| **T2DM With One CVD** | | | | | | | | | |
| Coronary Arterial Revascularization+T2DM | | | | | | | | | |
| Medication | 11 | 12 | 8,077 | 11 | 13 | 15,282 | 11 | 10 | 9,061 |
| Procedure | 11 | 4 | 17,268 | 11 | 6 | 16,181 | 11 | 5 | 3,623 |
| Consultation | 11 | 12 | 1,562 | 11 | 13 | 2,680 | 11 | 12 | 2,336 |
| Consumables | 3 | 1 | 1,053 | 4 | 1 | 1,142 | 5 | 2 | 435 |
| Services | 4 | 2 | 4,804 | 5 | 2 | 3,139 | 3 | 1 | 256 |
| Others | 3 | 1 | 63 | 5 | 1 | 530 | 1 | 1 | 250 |
| T2DM+Angina | | | | | | | | | |
| Medication | 156 | 12 | 5,699 | 156 | 14 | 8,297 | 156 | 11 | 5,302 |
| Procedure | 154 | 8 | 7,117 | 156 | 9 | 10,290 | 156 | 7 | 5,766 |
| Consultation | 156 | 13 | 2,241 | 156 | 15 | 2,818 | 156 | 12 | 1,678 |
| Consumables | 78 | 2 | 761 | 92 | 2 | 753 | 77 | 2 | 686 |
| Services | 52 | 2 | 3,467 | 63 | 2 | 2,300 | 54 | 2 | 1,921 |
| Others | 44 | 2 | 389 | 53 | 1 | 338 | 30 | 2 | 408 |
| T2DM+Atrial fibrillation | | | | | | | | | |
| Medication | 23 | 16 | 10,520 | 23 | 20 | 14,809 | 23 | 15 | 10,419 |
| Procedure | 22 | 11 | 16,271 | 23 | 13 | 14,716 | 22 | 8 | 8,208 |
| Consultation | 23 | 16 | 3,567 | 23 | 22 | 4,968 | 23 | 16 | 2,800 |
| Consumables | 16 | 3 | 1,393 | 18 | 3 | 1,300 | 14 | 2 | 844 |
| Services | 12 | 2 | 4,779 | 12 | 3 | 5,690 | 13 | 2 | 759 |
| Others | 7 | 2 | 410 | 8 | 2 | 536 | 3 | 2 | 335 |
| T2DM+cardiac ischemia | | | | | | | | | |
| Medication | 1 | 21 | 8,234 | 1 | 7 | 3,425 | 1 | 4 | 925 |
| Procedure | 1 | 12 | 26,990 | 1 | 3 | 3,621 | 1 | 5 | 3,354 |
| Consultation | 1 | 21 | 4,590 | 1 | 7 | 1,868 | 1 | 6 | 843 |
| Consumables | 1 | 1 | 1,229 |  |  |  | 1 | 1 | 16 |
| Services | 1 | 1 | 2,100 |  |  |  |  |  |  |
| Others | 1 | 1 | 5 |  |  |  |  |  |  |
| T2DM+Chronic renal failure | | | | | | | | | |
| Medication | 74 | 13 | 11,774 | 74 | 17 | 15,031 | 73 | 16 | 12,415 |
| Procedure | 74 | 9 | 14,188 | 74 | 13 | 22,616 | 73 | 12 | 23,887 |
| Consultation | 74 | 15 | 3,160 | 74 | 17 | 4,102 | 74 | 14 | 3,569 |
| Consumables | 35 | 3 | 1,315 | 42 | 2 | 826 | 47 | 2 | 960 |
| Services | 36 | 3 | 11,012 | 42 | 3 | 14,825 | 39 | 4 | 15,021 |
| Others | 22 | 2 | 462 | 21 | 2 | 634 | 24 | 2 | 386 |
| T2DM+Coronary Artery Disease | | | | | | | | | |
| Medication | 368 | 13 | 8,212 | 369 | 16 | 11,073 | 369 | 12 | 7,556 |
| Procedure | 355 | 7 | 8,659 | 358 | 9 | 10,972 | 355 | 7 | 7,014 |
| Consultation | 366 | 14 | 2,018 | 369 | 16 | 2,541 | 367 | 12 | 1,596 |
| Consumables | 156 | 2 | 1,343 | 158 | 2 | 735 | 158 | 2 | 760 |
| Services | 155 | 3 | 5,233 | 191 | 3 | 3,300 | 160 | 3 | 3,922 |
| Others | 106 | 2 | 370 | 97 | 2 | 423 | 81 | 2 | 331 |
| T2DM+Dysrhythmia | | | | | | | | | |
| Medication | 21 | 12 | 5,713 | 21 | 21 | 16,763 | 21 | 17 | 11,098 |
| Procedure | 20 | 8 | 8,560 | 21 | 16 | 22,124 | 21 | 9 | 10,032 |
| Consultation | 21 | 14 | 2,377 | 21 | 20 | 4,615 | 21 | 14 | 2,328 |
| Consumables | 5 | 1 | 840 | 8 | 2 | 3,243 | 11 | 2 | 468 |
| Services | 10 | 2 | 2,138 | 9 | 14 | 56,851 | 10 | 8 | 29,497 |
| Others | 4 | 2 | 155 | 7 | 1 | 226 | 6 | 1 | 244 |
| T2DM+Heart Failure | | | | | | | | | |
| Medication | 54 | 13 | 11,002 | 54 | 15 | 15,375 | 54 | 12 | 10,927 |
| Procedure | 54 | 8 | 13,508 | 54 | 10 | 18,798 | 52 | 8 | 9,681 |
| Consultation | 54 | 15 | 3,795 | 54 | 17 | 5,537 | 53 | 13 | 2,907 |
| Consumables | 31 | 2 | 878 | 28 | 3 | 1,911 | 33 | 3 | 1,008 |
| Services | 20 | 2 | 6,894 | 24 | 3 | 21,090 | 24 | 3 | 4,735 |
| Others | 21 | 2 | 339 | 17 | 2 | 387 | 8 | 2 | 281 |
| T2DM+Myocardial infarction | | | | | | | | | |
| Medication | 11 | 13 | 4,646 | 11 | 10 | 3,948 | 11 | 8 | 3,518 |
| Procedure | 11 | 7 | 4,794 | 11 | 5 | 15,828 | 11 | 5 | 3,401 |
| Consultation | 11 | 13 | 1,779 | 11 | 11 | 1,771 | 11 | 9 | 830 |
| Consumables | 4 | 2 | 342 | 1 | 4 | 1,646 | 6 | 2 | 258 |
| Services | 6 | 4 | 8,443 | 6 | 3 | 9,759 | 2 | 2 | 168 |
| Others | 1 | 1 | 240 | 2 | 2 | 328 | 1 | 1 | 67 |
| T2DM+Other Cardiovascular Disease | | | | | | | | | |
| Medication | 16 | 12 | 6,607 | 16 | 15 | 7,757 | 16 | 10 | 6,119 |
| Procedure | 15 | 9 | 8,962 | 16 | 9 | 13,592 | 15 | 7 | 11,731 |
| Consultation | 16 | 15 | 3,207 | 16 | 17 | 3,302 | 16 | 11 | 1,688 |
| Consumables | 11 | 2 | 757 | 11 | 2 | 908 | 9 | 2 | 1,097 |
| Services | 7 | 4 | 2,315 | 10 | 3 | 2,335 | 5 | 1 | 4,177 |
| Others | 8 | 1 | 623 | 3 | 1 | 783 | 2 | 1 | 175 |
| T2DM+Periphery vascular disease | | | | | | | | | |
| Medication | 4 | 6 | 5,871 | 4 | 11 | 10,776 | 4 | 9 | 8,805 |
| Procedure | 4 | 7 | 3,384 | 4 | 11 | 16,085 | 4 | 8 | 7,509 |
| Consultation | 4 | 8 | 1,190 | 4 | 14 | 3,293 | 4 | 9 | 1,411 |
| Consumables | 1 | 4 | 1,200 | 1 | 5 | 1,736 | 2 | 5 | 816 |
| Services | 2 | 37 | 1,359 | 2 | 34 | 20,810 |  |  |  |
| Others |  |  |  | 1 | 2 | 404 | 2 | 1 | 553 |
| T2DM+Stroke or TIA | | | | | | | | | |
| Medication | 170 | 13 | 8,807 | 171 | 16 | 11,547 | 171 | 13 | 8,754 |
| Procedure | 170 | 8 | 10,784 | 168 | 11 | 15,155 | 170 | 8 | 9,431 |
| Consultation | 170 | 15 | 2,924 | 171 | 18 | 4,998 | 170 | 13 | 2,658 |
| Consumables | 86 | 2 | 733 | 104 | 3 | 794 | 93 | 3 | 759 |
| Services | 73 | 3 | 5,707 | 89 | 3 | 6,311 | 61 | 3 | 7,204 |
| Others | 56 | 2 | 417 | 60 | 2 | 437 | 36 | 2 | 595 |
| **T2DM With Multiple CVD6,704** | | | | | | | | | |
| Coronary Arterial Revascularization+T2DM+Coronary Artery Disease | | | | | | | | | |
| Medication | 23 | 11 | 8,949 | 23 | 17 | 12,859 | 23 | 16 | 9,394 |
| Procedure | 21 | 8 | 16,611 | 23 | 10 | 15,560 | 23 | 8 | 20,937 |
| Consultation | 23 | 13 | 2,262 | 23 | 20 | 4,092 | 23 | 17 | 3,335 |
| Consumables | 15 | 2 | 4,676 | 7 | 2 | 2,211 | 13 | 1 | 2,326 |
| Services | 12 | 2 | 2,110 | 12 | 4 | 3,779 | 14 | 4 | 7,529 |
| Others | 8 | 1 | 620 | 5 | 1 | 3,356 | 7 | 2 | 399 |
| Coronary Arterial Revascularization+T2DM+Coronary Artery Disease+Angina | | | | | | | | | |
| Medication | 8 | 17 | 12,563 | 8 | 19 | 18,955 | 8 | 23 | 15,064 |
| Procedure | 8 | 11 | 12,267 | 8 | 11 | 21,590 | 8 | 10 | 15,001 |
| Consultation | 8 | 19 | 3,553 | 8 | 20 | 4,727 | 8 | 19 | 3,268 |
| Consumables | 6 | 1 | 667 | 6 | 2 | 727 | 4 | 3 | 448 |
| Services | 5 | 2 | 2,347 | 5 | 3 | 1,990 | 4 | 3 | 3,697 |
| Others | 2 | 1 | 29 | 4 | 3 | 2,963 | 3 | 1 | 142 |
| T2DM+Coronary Artery Disease+Angina | | | | | | | | | |
| Medication | 76 | 15 | 8,539 | 76 | 19 | 14,188 | 76 | 14 | 7,951 |
| Procedure | 73 | 9 | 9,463 | 73 | 10 | 23,808 | 74 | 8 | 12,922 |
| Consultation | 76 | 15 | 2,193 | 76 | 19 | 3,364 | 76 | 14 | 2,180 |
| Consumables | 27 | 2 | 1,098 | 35 | 2 | 2,083 | 37 | 2 | 897 |
| Services | 36 | 3 | 7,321 | 50 | 3 | 7,343 | 44 | 3 | 4,496 |
| Others | 16 | 1 | 391 | 18 | 2 | 1,298 | 18 | 2 | 334 |
| T2DM+Coronary Artery Disease+Atrial fibrillation | | | | | | | | | |
| Medication | 8 | 17 | 8,276 | 8 | 16 | 10,712 | 8 | 17 | 9,690 |
| Procedure | 8 | 12 | 6,853 | 7 | 11 | 12,350 | 8 | 13 | 15,223 |
| Consultation | 8 | 18 | 2,420 | 8 | 17 | 2,483 | 8 | 19 | 2,659 |
| Consumables | 4 | 3 | 1,635 | 3 | 2 | 1,287 | 6 | 2 | 634 |
| Services | 4 | 6 | 14,293 | 5 | 2 | 8,145 | 4 | 2 | 7,127 |
| Others | 3 | 4 | 547 | 2 | 5 | 1,345 | 4 | 2 | 675 |
| T2DM+Coronary Artery Disease+Chronic renal failure | | | | | | | | | |
| Medication | 14 | 13 | 10,567 | 14 | 17 | 20,609 | 14 | 14 | 16,688 |
| Procedure | 13 | 8 | 27,268 | 13 | 13 | 17,425 | 14 | 10 | 16,582 |
| Consultation | 13 | 15 | 3,239 | 14 | 16 | 4,478 | 14 | 13 | 2,742 |
| Consumables | 6 | 1 | 624 | 9 | 2 | 765 | 7 | 3 | 969 |
| Services | 6 | 4 | 6,623 | 8 | 4 | 9,426 | 11 | 3 | 7,412 |
| Others | 4 | 2 | 520 | 5 | 1 | 779 | 4 | 1 | 396 |
| T2DM+Heart Failure+Angina | | | | | | | | | |
| Medication | 8 | 12 | 7,365 | 8 | 19 | 9,637 | 8 | 17 | 10,657 |
| Procedure | 8 | 8 | 8,659 | 8 | 11 | 17,402 | 8 | 13 | 21,656 |
| Consultation | 8 | 15 | 2,778 | 8 | 20 | 3,892 | 8 | 21 | 3,858 |
| Consumables | 5 | 2 | 562 | 5 | 3 | 1,453 | 4 | 7 | 1,350 |
| Services | 5 | 3 | 2,091 | 4 | 3 | 5,504 | 3 | 3 | 4,258 |
| Others |  |  |  | 3 | 1 | 237 | 1 | 1 | 0 |
| Abbreviations: CVD=Cardiovascular disease, HCRU=Healthcare cost utilization, N=Number of patients, T2DM=Type 2 diabetes mellitus, TIA=Transient ischemic attack | | | | | | | | | |
