# Supplementary material for: Cost of cardiovascular diseases and renal complications in people with type 2 diabetes mellitus in the Kingdom of Saudi Arabia: A retrospective analysis of claims database
Source: PLoS One. 2022 Oct 20;17(10):e0273836. doi: 10.1371/journal.pone.0273836 (PMC9584438; doi:10.1371/journal.pone.0273836)
Supplement: S14 Table — (DOCX) [file pone.0273836.s014.docx]

### S14 Table: Comparison of pre-index and post-index all-cause cost for various activities (Payer 3, Cohort 3)

| **All-Cause** | **Pre-Index 1 Yr** | | | **Post-Index 1 Yr** | | | **Post-Index 2 Yr** | | | **Post-Index 3 Yr** | | | |
| --- | --- | --- | --- | --- | --- | --- | --- | --- | --- | --- | --- | --- | --- |
| **Payer 3** | **All-Cause** |  |  | **All -Cause** |  |  | **All- Cause** |  |  | **All- Cause** |  |  | |
| **Cohort 3** | **N** | **HCRU** | **Cost** | **N** | **HCRU** | **Cost** | **N** | **HCRU** | **Cost** | **N** | **HCRU** | **Cost** | |
| **T2DM WITH ONE CVD** | | | | | | | | | | | | | |
| **Coronary Arterial Revascularization+T2DM** | **21** | **36** | **42,639** | **20** | **41** | **61,389** | **20** | **33** | **20,918** | **21** | **23** | **16,360** | |
| Medication | 5 | 13 | 12,616 | 5 | 15 | 22,669 | 5 | 11 | 13,089 | 5 | 8 | 7,518 | |
| Procedure | 5 | 5 | 23,488 | 5 | 7 | 26,144 | 5 | 5 | 4,734 | 5 | 4 | 5,302 | |
| Consultation | 5 | 14 | 2,106 | 5 | 16 | 3,099 | 5 | 13 | 2,152 | 5 | 8 | 1,758 | |
| Consumables | 2 | 1 | 1,170 | 2 | 1 | 1,225 | 3 | 2 | 602 | 4 | 2 | 728 | |
| Services | 2 | 1 | 3,244 | 2 | 2 | 6,872 | 1 | 1 | 90 | 2 | 1 | 1,055 | |
| Others | 2 | 2 | 15 | 1 | 1 | 1,380 | 1 | 1 | 250 |  |  |  | |
| **T2DM+Angina** | **144** | **35** | **12,192** | **165** | **47** | **29,374** | **157** | **42** | **22,068** | **151** | **33** | **16,934** | |
| Medication | 37 | 11 | 4,682 | 37 | 15 | 11,007 | 37 | 13 | 7,851 | 37 | 10 | 4,613 | |
| Procedure | 37 | 7 | 4,221 | 37 | 10 | 12,277 | 37 | 9 | 7,407 | 37 | 7 | 6,845 | |
| Consultation | 37 | 12 | 1,742 | 37 | 16 | 3,245 | 37 | 14 | 2,271 | 37 | 10 | 1,729 | |
| Consumables | 17 | 2 | 934 | 21 | 2 | 712 | 20 | 2 | 654 | 21 | 2 | 542 | |
| Services | 8 | 1 | 246 | 17 | 3 | 1,814 | 17 | 2 | 3,202 | 12 | 2 | 3,054 | |
| Others | 8 | 1 | 368 | 16 | 1 | 319 | 9 | 2 | 683 | 7 | 2 | 152 | |
| **T2DM+Atrial fibrillation** | **13** | **31** | **21,499** | **13** | **58** | **36,439** | **14** | **46** | **21,689** | **16** | **44** | **19,105** | |
| Medication | 3 | 11 | 7,636 | 3 | 19 | 12,713 | 3 | 18 | 12,257 | 3 | 16 | 8,235 | |
| Procedure | 3 | 5 | 5,478 | 3 | 10 | 10,898 | 3 | 6 | 4,057 | 3 | 6 | 7,301 | |
| Consultation | 3 | 12 | 1,673 | 3 | 25 | 5,989 | 3 | 18 | 3,400 | 3 | 18 | 2,098 | |
| Consumables | 2 | 1 | 715 | 2 | 2 | 641 | 2 | 1 | 550 | 3 | 1 | 263 | |
| Services | 1 | 1 | 5,797 | 1 | 1 | 5,797 | 1 | 1 | 975 | 2 | 1 | 202 | |
| Others | 1 | 1 | 200 | 1 | 2 | 400 | 2 | 2 | 450 | 2 | 2 | 1,005 | |
| **T2DM+Chronic renal failure** | **55** | **43** | **29,807** | **60** | **54** | **56,003** | **61** | **47** | **49,460** | **56** | **43** | **21,508** | |
| Medication | 13 | 13 | 8,929 | 13 | 16 | 14,066 | 13 | 14 | 12,977 | 13 | 13 | 8,011 | |
| Procedure | 13 | 9 | 6,478 | 13 | 14 | 19,576 | 13 | 10 | 21,528 | 13 | 8 | 7,715 | |
| Consultation | 13 | 14 | 2,569 | 13 | 18 | 5,264 | 13 | 15 | 3,890 | 13 | 14 | 2,549 | |
| Consumables | 9 | 2 | 1,710 | 11 | 2 | 628 | 11 | 3 | 1,099 | 9 | 3 | 710 | |
| Services | 3 | 4 | 9,743 | 5 | 3 | 15,898 | 6 | 3 | 9,727 | 5 | 3 | 2,391 | |
| Others | 4 | 1 | 378 | 5 | 1 | 572 | 5 | 1 | 239 | 3 | 1 | 133 | |
| **T2DM+Coronary Artery Disease** | **233** | **38** | **15,049** | **253** | **48** | **26,430** | **242** | **45** | **19,440** | **224** | **31** | **11,830** | |
| Medication | 59 | 13 | 5,060 | 59 | 16 | 12,449 | 59 | 15 | 7,563 | 59 | 11 | 5,154 | |
| Procedure | 58 | 7 | 5,365 | 58 | 9 | 8,683 | 59 | 8 | 5,832 | 58 | 6 | 4,378 | |
| Consultation | 59 | 14 | 1,126 | 59 | 17 | 2,230 | 59 | 15 | 1,344 | 59 | 10 | 817 | |
| Consumables | 22 | 1 | 1,388 | 27 | 2 | 654 | 26 | 2 | 491 | 24 | 1 | 433 | |
| Services | 20 | 2 | 1,747 | 31 | 2 | 2,022 | 22 | 4 | 3,862 | 13 | 2 | 960 | |
| Others | 15 | 2 | 363 | 19 | 2 | 392 | 17 | 2 | 348 | 11 | 1 | 89 | |
| **T2DM+Dysrhythmia** | **33** | **37** | **16,904** | **36** | **51** | **33,879** | **35** | **48** | **29,144** | **31** | **32** | **12,570** | |
| Medication | 8 | 12 | 5,898 | 8 | 18 | 21,511 | 8 | 16 | 13,880 | 8 | 11 | 5,157 | |
| Procedure | 8 | 7 | 4,236 | 8 | 10 | 8,219 | 8 | 10 | 7,485 | 8 | 6 | 4,103 | |
| Consultation | 8 | 13 | 1,473 | 8 | 19 | 3,375 | 8 | 16 | 1,695 | 8 | 11 | 1,141 | |
| Consumables | 2 | 2 | 400 | 5 | 1 | 424 | 5 | 2 | 563 | 4 | 2 | 369 | |
| Services | 3 | 2 | 4,742 | 2 | 1 | 71 | 4 | 2 | 5,047 | 2 | 1 | 931 | |
| Others | 4 | 2 | 155 | 5 | 1 | 280 | 2 | 2 | 474 | 1 | 1 | 870 | |
| **T2DM+Heart Failure** | **41** | **48** | **23,932** | **44** | **62** | **94,604** | **42** | **51** | **51,413** | **45** | **49** | **30,766** | |
| Medication | 10 | 16 | 12,857 | 10 | 18 | 28,303 | 10 | 17 | 20,964 | 10 | 17 | 13,101 | |
| Procedure | 10 | 7 | 5,655 | 10 | 13 | 32,352 | 10 | 9 | 11,905 | 10 | 9 | 8,956 | |
| Consultation | 10 | 17 | 2,809 | 10 | 20 | 9,305 | 10 | 18 | 4,581 | 10 | 16 | 3,499 | |
| Consumables | 7 | 3 | 955 | 7 | 5 | 3,434 | 7 | 4 | 1,755 | 8 | 4 | 998 | |
| Services | 2 | 4 | 1,516 | 4 | 4 | 21,211 | 3 | 2 | 11,958 | 5 | 2 | 4,212 | |
| Others | 2 | 1 | 140 | 3 | 2 | 0 | 2 | 2 | 250 | 2 | 2 | 0 | |
| **T2DM+Myocardial infarction** | **4** | **62** | **9,125** | **3** | **59** | **6,706** | **3** | **57** | **4,409** | **4** | **26** | **2,056** | |
| Medication | 1 | 29 | 5,230 | 1 | 31 | 4,816 | 1 | 27 | 2,979 | 1 | 14 | 1,631 | |
| Procedure | 1 | 11 | 2,415 | 1 | 6 | 790 | 1 | 7 | 560 | 1 | 2 | 110 | |
| Consultation | 1 | 21 | 1,450 | 1 | 22 | 1,100 | 1 | 23 | 870 | 1 | 8 | 270 | |
| Consumables |  |  |  |  |  |  |  |  |  |  |  |  | |
| Services | 1 | 1 | 30 |  |  |  |  |  |  | 1 | 2 | 45 | |
| Others |  |  |  |  |  |  |  |  |  |  |  |  | |
| **T2DM+Other Cardiovascular Disease** | **19** | **50** | **23,055** | **20** | **63** | **51,813** | **15** | **38** | **26,609** | **16** | **25** | **17,646** | |
| Medication | 4 | 12 | 5,454 | 4 | 17 | 13,520 | 4 | 13 | 13,005 | 4 | 8 | 11,243 | |
| Procedure | 4 | 13 | 10,780 | 4 | 16 | 26,995 | 4 | 10 | 10,589 | 4 | 7 | 4,658 | |
| Consultation | 4 | 15 | 2,949 | 4 | 20 | 5,969 | 4 | 13 | 2,290 | 4 | 8 | 1,493 | |
| Consumables | 2 | 3 | 423 | 4 | 3 | 903 | 3 | 3 | 725 | 3 | 1 | 253 | |
| Services | 3 | 6 | 2,460 | 3 | 6 | 4,075 |  |  |  |  |  |  | |
| Others | 2 | 1 | 990 | 1 | 1 | 350 |  |  |  | 1 | 1 | 0 | |
| **T2DM+Periphery vascular disease** | **9** | **69** | **19,630** | **9** | **130** | **98,822** | **9** | **49** | **32,738** | **9** | **69** | **117,303** | |
| Medication | 2 | 8 | 10,356 | 2 | 16 | 19,203 | 2 | 13 | 14,662 | 2 | 19 | 18,721 | |
| Procedure | 2 | 11 | 4,930 | 2 | 19 | 29,826 | 2 | 14 | 13,485 | 2 | 13 | 35,413 | |
| Consultation | 2 | 10 | 1,785 | 2 | 21 | 6,061 | 2 | 14 | 2,474 | 2 | 20 | 3,865 | |
| Consumables | 1 | 4 | 1,200 | 1 | 5 | 1,736 | 1 | 8 | 1,565 | 2 | 6 | 1,126 | |
| Services | 2 | 37 | 1,359 | 1 | 67 | 41,591 |  |  |  | 1 | 12 | 58,178 | |
| Others |  |  |  | 1 | 2 | 404 | 2 | 1 | 553 |  |  |  | |
| **T2DM+Stroke or TIA** | **132** | **37** | **26,154** | **142** | **51** | **36,316** | **143** | **46** | **24,981** | **131** | **35** | **20,998** | |
| Medication | 32 | 12 | 6,297 | 32 | 16 | 12,591 | 32 | 15 | 10,062 | 31 | 11 | 5,959 | |
| Procedure | 31 | 8 | 11,057 | 32 | 10 | 15,300 | 32 | 10 | 10,167 | 32 | 8 | 9,941 | |
| Consultation | 32 | 13 | 2,941 | 32 | 18 | 5,760 | 32 | 15 | 3,004 | 32 | 11 | 2,061 | |
| Consumables | 19 | 2 | 529 | 24 | 2 | 909 | 24 | 2 | 753 | 20 | 2 | 1,625 | |
| Services | 9 | 2 | 4,774 | 13 | 2 | 990 | 10 | 2 | 575 | 9 | 2 | 1,209 | |
| Others | 9 | 2 | 557 | 9 | 2 | 767 | 13 | 2 | 421 | 7 | 1 | 203 | |
| **T2DM WITH MULTIPLE CVD** | | | | | | | | | | | | | |
| **Coronary Arterial Revascularization+T2DM+Coronary Artery Disease** | **17** | **37** | **18,905** | **16** | **43** | **47,819** | **17** | **51** | **25,424** | **17** | **34** | **69,291** | |
| Medication | 4 | 10 | 6,061 | 4 | 15 | 16,327 | 4 | 17 | 12,044 | 4 | 11 | 7,375 | |
| Procedure | 3 | 9 | 7,288 | 4 | 6 | 8,827 | 4 | 9 | 7,800 | 3 | 6 | 56,056 | |
| Consultation | 4 | 12 | 1,701 | 4 | 15 | 2,793 | 4 | 18 | 3,424 | 4 | 12 | 1,748 | |
| Consumables | 2 | 5 | 1,408 | 1 | 5 | 1,500 | 2 | 2 | 634 | 3 | 2 | 2,351 | |
| Services | 2 | 1 | 258 | 2 | 2 | 2,372 | 2 | 3 | 260 | 2 | 2 | 1,311 | |
| Others | 2 | 2 | 2,190 | 1 | 1 | 16,000 | 1 | 4 | 1,262 | 1 | 1 | 450 | |
| **Coronary Arterial Revascularization+T2DM+Coronary Artery Disease+Angina** | **14** | **64** | **35,527** | **13** | **68** | **52,633** | **12** | **77** | **36,167** | **15** | **82** | **49,214** | |
| Medication | 3 | 23 | 16,010 | 3 | 23 | 28,476 | 3 | 35 | 25,059 | 3 | 31 | 18,553 | |
| Procedure | 3 | 13 | 12,121 | 3 | 13 | 17,592 | 3 | 10 | 7,067 | 3 | 15 | 23,009 | |
| Consultation | 3 | 24 | 2,194 | 3 | 24 | 3,029 | 3 | 25 | 2,559 | 3 | 31 | 4,888 | |
| Consumables | 2 | 1 | 735 | 2 | 2 | 913 | 1 | 1 | 50 | 2 | 2 | 1,303 | |
| Services | 2 | 3 | 4,448 | 1 | 5 | 2,304 | 1 | 5 | 1,082 | 2 | 2 | 1,034 | |
| Others | 1 | 1 | 19 | 1 | 1 | 320 | 1 | 1 | 350 | 2 | 2 | 428 | |
| **Coronary Arterial Revascularization+T2DM+Heart Failure+Coronary Artery Disease** | **8** | **28** | **112,189** | **8** | **33** | **21,976** | **8** | **34** | **11,680** | **7** | **31** | **16,204** | |
| Medication | 2 | 9 | 3,691 | 2 | 10 | 14,509 | 2 | 11 | 7,560 | 2 | 10 | 5,559 | |
| Procedure | 2 | 6 | 6,067 | 2 | 5 | 2,861 | 2 | 5 | 1,233 | 1 | 5 | 3,942 | |
| Consultation | 2 | 11 | 1,068 | 2 | 12 | 2,694 | 2 | 13 | 1,315 | 2 | 10 | 952 | |
| Consumables | 1 | 1 | 51 | 1 | 5 | 1,448 | 1 | 4 | 1,473 | 1 | 4 | 2,600 | |
| Services | 1 | 1 | 101,312 | 1 | 1 | 465 | 1 | 1 | 100 | 1 | 2 | 3,150 | |
| Others |  |  |  |  |  |  |  |  |  |  |  |  | |
| **T2DM+Chronic renal failure+Angina** | **7** | **37** | **16,418** | **7** | **62** | **54,244** | **8** | **45** | **40,991** | **9** | **45** | **29,681** | |
| Medication | 2 | 11 | 7,070 | 2 | 18 | 18,563 | 2 | 15 | 18,925 | 2 | 13 | 11,899 | |
| Procedure | 2 | 10 | 6,225 | 2 | 19 | 23,613 | 2 | 12 | 15,909 | 2 | 12 | 12,393 | |
| Consultation | 2 | 15 | 2,953 | 2 | 23 | 9,088 | 2 | 17 | 6,065 | 2 | 17 | 4,439 | |
| Consumables | 1 | 1 | 170 | 1 | 2 | 2,979 | 2 | 1 | 92 | 2 | 2 | 850 | |
| Services |  |  |  |  |  |  |  |  |  |  |  |  | |
| Others |  |  |  |  |  |  |  |  |  | 1 | 1 | 100 | |
| **T2DM+Coronary Artery Disease+Angina** | **50** | **48** | **23,682** | **47** | **52** | **60,669** | **48** | **46** | **25,410** | **46** | **39** | **39,710** | |
| Medication | 11 | 14 | 7,629 | 11 | 16 | 18,833 | 11 | 13 | 8,973 | 11 | 12 | 19,619 | |
| Procedure | 11 | 12 | 9,884 | 11 | 11 | 26,941 | 11 | 10 | 9,695 | 11 | 9 | 11,607 | |
| Consultation | 11 | 17 | 2,354 | 11 | 18 | 5,067 | 11 | 16 | 2,788 | 11 | 11 | 2,538 | |
| Consumables | 7 | 1 | 370 | 6 | 2 | 5,027 | 6 | 3 | 863 | 5 | 3 | 895 | |
| Services | 5 | 2 | 2,965 | 6 | 3 | 3,677 | 7 | 3 | 2,526 | 4 | 2 | 4,924 | |
| Others | 5 | 2 | 480 | 2 | 2 | 1,125 | 2 | 2 | 565 | 4 | 1 | 127 | |
| **T2DM+Coronary Artery Disease+Atrial fibrillation** | **9** | **47** | **13,381** | **10** | **63** | **33,167** | **9** | **60** | **29,465** | **7** | **41** | **20,688** | |
| Medication | 2 | 14 | 8,720 | 2 | 18 | 18,342 | 2 | 21 | 15,863 | 2 | 13 | 8,873 | |
| Procedure | 2 | 11 | 3,186 | 2 | 16 | 7,919 | 2 | 16 | 9,839 | 2 | 10 | 9,541 | |
| Consultation | 2 | 14 | 1,205 | 2 | 21 | 2,355 | 2 | 20 | 2,663 | 2 | 13 | 1,315 | |
| Consumables | 2 | 4 | 271 | 1 | 2 | 1,200 | 2 | 1 | 300 | 1 | 5 | 958 | |
| Services |  |  |  | 2 | 1 | 2,550 |  |  |  |  |  |  | |
| Others | 1 | 5 | 0 | 1 | 6 | 800 | 1 | 3 | 800 |  |  |  | |
| **T2DM+Coronary Artery Disease+Chronic renal failure** | **22** | **26** | **29,285** | **20** | **30** | **41,492** | **22** | **31** | **25,820** | **19** | **20** | **15,997** | |
| Medication | 5 | 8 | 11,583 | 5 | 9 | 17,846 | 5 | 10 | 15,070 | 5 | 6 | 11,346 | |
| Procedure | 4 | 6 | 7,321 | 4 | 7 | 9,486 | 5 | 7 | 6,585 | 5 | 4 | 3,198 | |
| Consultation | 5 | 9 | 2,054 | 5 | 9 | 2,962 | 5 | 10 | 2,275 | 5 | 7 | 1,188 | |
| Consumables | 3 | 1 | 597 | 4 | 2 | 959 | 3 | 2 | 998 | 2 | 2 | 170 | |
| Services | 2 | 1 | 7,370 | 1 | 1 | 9,740 | 3 | 1 | 742 |  |  |  | |
| Others | 3 | 1 | 360 | 1 | 2 | 500 | 1 | 1 | 150 | 2 | 1 | 95 | |
| **T2DM+Heart Failure+Angina** | **9** | **29** | **13,804** | **8** | **27** | **25,792** | **7** | **62** | **55,951** | **9** | **41** | **32,885** | |
| Medication | 2 | 8 | 4,634 | 2 | 8 | 5,379 | 2 | 17 | 12,899 | 2 | 10 | 7,775 | |
| Procedure | 2 | 6 | 5,314 | 2 | 4 | 13,239 | 2 | 14 | 36,509 | 2 | 13 | 18,466 | |
| Consultation | 2 | 11 | 1,984 | 2 | 13 | 2,564 | 2 | 21 | 5,155 | 2 | 11 | 2,604 | |
| Consumables | 2 | 2 | 333 | 1 | 1 | 4,600 | 1 | 11 | 1,387 | 1 | 4 | 336 | |
| Services | 1 | 3 | 1,540 |  |  |  |  |  |  | 1 | 2 | 3,705 | |
| Others |  |  |  | 1 | 1 | 11 |  |  |  | 1 | 2 | 0 | |
| **T2DM+Heart Failure+Coronary Artery Disease** | **14** | **40** | **23,452** | **14** | **62** | **39,686** | **15** | **47** | **17,270** | **13** | **40** | **17,386** | |
| Medication | 3 | 12 | 4,678 | 3 | 22 | 15,675 | 3 | 17 | 9,934 | 3 | 14 | 7,293 | |
| Procedure | 3 | 7 | 5,901 | 3 | 8 | 7,964 | 3 | 9 | 4,861 | 3 | 7 | 8,157 | |
| Consultation | 3 | 13 | 2,966 | 3 | 22 | 3,213 | 3 | 17 | 1,843 | 3 | 15 | 1,398 | |
| Consumables | 1 | 1 | 20 | 1 | 3 | 1,970 | 3 | 1 | 82 | 1 | 2 | 174 | |
| Services | 3 | 6 | 9,852 | 3 | 4 | 10,181 | 1 | 2 | 375 | 3 | 2 | 363 | |
| Others | 1 | 1 | 35 | 1 | 3 | 683 | 2 | 1 | 175 |  |  |  | |
| **T2DM+Heart Failure+Coronary Artery Disease+Atrial fibrillation** | **10** | **33** | **13,762** | **9** | **43** | **54,564** | **10** | **35** | **33,987** | **7** | **13** | **8,076** | |
| Medication | 2 | 9 | 3,271 | 2 | 13 | 14,122 | 2 | 13 | 9,340 | 2 | 5 | 4,145 | |
| Procedure | 2 | 9 | 6,809 | 2 | 8 | 17,406 | 2 | 7 | 15,454 | 2 | 2 | 1,281 | |
| Consultation | 2 | 13 | 3,087 | 2 | 17 | 5,517 | 2 | 10 | 3,321 | 2 | 5 | 1,140 | |
| Consumables | 1 | 1 | 50 | 2 | 2 | 1,086 | 2 | 3 | 1,058 |  |  |  | |
| Services | 1 | 1 | 220 | 1 | 3 | 16,433 | 1 | 2 | 4,814 | 1 | 1 | 1,509 | |
| Others | 2 | 1 | 325 |  |  |  | 1 | 1 | 0 |  |  |  | |
| **T2DM+Myocardial infarction+Coronary Artery Disease** | **24** | **40** | **29,696** | **23** | **57** | **74,818** | **22** | **42** | **17,604** | **21** | **34** | **14,895** | |
| Medication | 5 | 11 | 11,108 | 5 | 17 | 20,531 | 5 | 15 | 8,501 | 5 | 11 | 5,513 | |
| Procedure | 5 | 7 | 10,609 | 5 | 10 | 35,780 | 5 | 6 | 4,899 | 5 | 6 | 5,258 | |
| Consultation | 5 | 13 | 1,833 | 5 | 22 | 6,017 | 5 | 16 | 2,128 | 5 | 12 | 1,706 | |
| Consumables | 3 | 4 | 583 | 2 | 2 | 1,194 | 3 | 1 | 743 | 3 | 2 | 539 | |
| Services | 3 | 3 | 5,281 | 4 | 5 | 10,726 | 2 | 3 | 1,060 | 2 | 1 | 1,294 | |
| Others | 3 | 1 | 281 | 2 | 2 | 570 | 2 | 1 | 273 | 1 | 2 | 585 | |
| **T2DM+Stroke or TIA+Angina** | **10** | **29** | **8,769** | **11** | **36** | **15,784** | **14** | **46** | **18,610** | **9** | **28** | **10,201** | |
| Medication | 3 | 8 | 2,549 | 3 | 13 | 6,774 | 3 | 17 | 6,136 | 3 | 9 | 3,780 | |
| Procedure | 2 | 8 | 5,240 | 3 | 5 | 5,769 | 3 | 9 | 7,403 | 2 | 8 | 3,970 | |
| Consultation | 3 | 10 | 934 | 3 | 14 | 2,977 | 3 | 17 | 2,475 | 3 | 10 | 1,551 | |
| Consumables | 1 | 1 | 47 | 2 | 3 | 264 | 2 | 2 | 633 | 1 | 2 | 900 | |
| Services |  |  |  |  |  |  | 3 | 1 | 1,963 |  |  |  | |
| Others | 1 | 2 | 0 |  |  |  |  |  |  |  |  |  | |
| **T2DM+Stroke or TIA+Chronic renal failure** | **23** | **38** | **20,858** | **23** | **60** | **58,889** | **22** | **62** | **46,889** | **22** | **36** | **27,918** | |
| Medication | 5 | 10 | 5,868 | 5 | 17 | 15,341 | 5 | 19 | 12,802 | 5 | 10 | 6,677 | |
| Procedure | 5 | 10 | 8,692 | 5 | 13 | 21,240 | 5 | 11 | 20,756 | 5 | 9 | 14,557 | |
| Consultation | 5 | 12 | 2,244 | 5 | 19 | 5,895 | 5 | 23 | 5,092 | 5 | 12 | 4,457 | |
| Consumables | 4 | 3 | 431 | 3 | 4 | 892 | 3 | 2 | 334 | 2 | 2 | 225 | |
| Services | 2 | 3 | 3,057 | 3 | 6 | 15,052 | 2 | 5 | 7,751 | 3 | 2 | 1,652 | |
| Others | 2 | 2 | 565 | 2 | 2 | 471 | 2 | 2 | 155 | 2 | 2 | 350 | |
| **T2DM+Stroke or TIA+Coronary Artery Disease** | **20** | **50** | **22,689** | **17** | **54** | **44,037** | **20** | **53** | **21,540** | **12** | **19** | **9,491** | |
| Medication | 4 | 18 | 6,889 | 4 | 18 | 13,101 | 4 | 20 | 9,803 | 4 | 7 | 3,757 | |
| Procedure | 3 | 9 | 10,135 | 4 | 9 | 16,987 | 4 | 8 | 5,271 | 3 | 4 | 3,273 | |
| Consultation | 4 | 18 | 2,469 | 4 | 21 | 8,546 | 4 | 19 | 2,508 | 4 | 7 | 1,015 | |
| Consumables | 3 | 2 | 124 |  |  |  | 2 | 1 | 295 |  |  |  | |
| Services | 3 | 2 | 2,876 | 4 | 5 | 5,372 | 3 | 4 | 3,541 | 1 | 1 | 1,446 | |
| Others | 3 | 1 | 197 | 1 | 1 | 30 | 3 | 2 | 123 |  |  |  | |
| **T2DM+Stroke or TIA+Coronary Artery Disease+Atrial fibrillation** | **8** | **36** | **17,442** | **11** | **50** | **32,110** | **10** | **72** | **50,614** | **11** | **61** | **24,739** | |
| Medication | 2 | 9 | 5,350 | 2 | 15 | 9,405 | 2 | 23 | 14,377 | 2 | 21 | 10,278 | |
| Procedure | 2 | 11 | 9,710 | 2 | 12 | 16,770 | 2 | 18 | 25,986 | 2 | 16 | 9,893 | |
| Consultation | 2 | 12 | 1,983 | 2 | 15 | 3,786 | 2 | 21 | 3,664 | 2 | 20 | 3,158 | |
| Consumables | 1 | 3 | 199 | 1 | 4 | 286 | 1 | 6 | 574 | 2 | 3 | 497 | |
| Services | 1 | 1 | 200 | 2 | 3 | 1,375 | 2 | 3 | 5,898 | 2 | 1 | 263 | |
| Others |  |  |  | 2 | 2 | 487 | 1 | 2 | 116 | 1 | 1 | 650 | |
| Abbreviations: CVD=Cardiovascular disease, HCRU=Healthcare cost utilization, N=Number of patients, T2DM=Type 2 diabetes mellitus, TIA=Transient ischemic attack | | | | | | | | | | | | |  |
|  | | | | | | | | | | | | |  |
