# Supplementary material for: Cost of cardiovascular diseases and renal complications in people with type 2 diabetes mellitus in the Kingdom of Saudi Arabia: A retrospective analysis of claims database
Source: PLoS One. 2022 Oct 20;17(10):e0273836. doi: 10.1371/journal.pone.0273836 (PMC9584438; doi:10.1371/journal.pone.0273836)
Supplement: S15 Table — (DOCX) [file pone.0273836.s015.docx]

### S15 Table: Comparison of pre-index and post-index disease-specific cause cost for various activities (Payer 1, Cohort 1)

| **Disease-specific Cause** | **Pre-Index 1 Yr** | | | **Post-Index 1 Yr** | | |
| --- | --- | --- | --- | --- | --- | --- |
| **Payer 1** | **Disease-specific Cause** | | | **Disease-specific Cause** | | |
| **Cohort 1** | **N** | **HCRU** | **Cost** | **N** | **HCRU** | **Cost** |
| **T2DM With One CVD4** | | | | | | |
| T2DM+Angina | | | | | | |
| Medication | 160 | 4 | 1,611 | 137 | 5 | 2,116 |
| Procedure | 159 | 3 | 948 | 140 | 3 | 1,172 |
| Consultation | 162 | 4 | 167 | 145 | 4 | 163 |
| Consumables | 18 | 2 | 221 | 20 | 2 | 266 |
| Services | 10 | 1 | 85 | 12 | 1 | 98 |
| Others | 23 | 1 | 279 | 20 | 2 | 456 |
| T2DM+Atrial fibrillation | | | | | | |
| Medication | 46 | 4 | 1,774 | 45 | 5 | 3,133 |
| Procedure | 40 | 3 | 1,513 | 40 | 3 | 2,797 |
| Consultation | 46 | 3 | 252 | 45 | 5 | 551 |
| Consumables | 9 | 1 | 298 | 11 | 1 | 755 |
| Services | 5 | 1 | 655 | 12 | 1 | 2,214 |
| Others | 5 | 1 | 1,000 | 9 | 1 | 2,173 |
| T2DM+cardiac ischemia | | | | | | |
| Medication |  |  |  | 1 | 4 | 924 |
| Procedure |  |  |  |  |  |  |
| Consultation |  |  |  | 1 | 3 | 45 |
| Consumables |  |  |  |  |  |  |
| Services |  |  |  |  |  |  |
| Others |  |  |  |  |  |  |
| T2DM+Chronic renal failure | | | | | | |
| Medication | 107 | 5 | 2,940 | 111 | 7 | 4,493 |
| Procedure | 106 | 4 | 2,002 | 102 | 5 | 7,935 |
| Consultation | 107 | 4 | 422 | 109 | 5 | 655 |
| Consumables | 17 | 2 | 263 | 32 | 3 | 2,125 |
| Services | 8 | 1 | 278 | 18 | 2 | 1,987 |
| Others | 27 | 2 | 577 | 30 | 3 | 490 |
| T2DM+Coronary Arterial Revascularization | | | | | | |
| Medication | 1 | 2 | 443 | 1 | 4 | 832 |
| Procedure | 1 | 1 | 630 | 1 | 1 | 270 |
| Consultation | 1 | 2 | 325 | 1 | 4 | 220 |
| Consumables |  |  |  |  |  |  |
| Services |  |  |  |  |  |  |
| Others |  |  |  |  |  |  |
| T2DM+Coronary Artery Disease | | | | | | |
| Medication | 1,520 | 5 | 2,386 | 1,560 | 5 | 3,121 |
| Procedure | 1,354 | 3 | 1,427 | 1,387 | 3 | 2,374 |
| Consultation | 1,398 | 4 | 296 | 1,482 | 4 | 360 |
| Consumables | 186 | 2 | 283 | 274 | 2 | 1,236 |
| Services | 123 | 2 | 836 | 146 | 2 | 778 |
| Others | 303 | 2 | 374 | 364 | 2 | 615 |
| T2DM+Dysrhythmia | | | | | | |
| Medication | 22 | 4 | 1,530 | 22 | 4 | 1,828 |
| Procedure | 20 | 3 | 999 | 17 | 2 | 1,219 |
| Consultation | 20 | 4 | 241 | 22 | 3 | 215 |
| Consumables | 3 | 3 | 1,132 | 3 | 2 | 282 |
| Services | 1 | 1 | 2,368 | 2 | 1 | 83 |
| Others | 5 | 1 | 266 | 2 | 2 | 448 |
| T2DM+Heart Failure | | | | | | |
| Medication | 55 | 4 | 1,571 | 60 | 5 | 2,024 |
| Procedure | 49 | 3 | 1,354 | 50 | 3 | 1,940 |
| Consultation | 52 | 4 | 295 | 50 | 4 | 400 |
| Consumables | 4 | 2 | 228 | 8 | 1 | 269 |
| Services | 5 | 1 | 282 | 7 | 1 | 1,475 |
| Others | 14 | 1 | 273 | 7 | 1 | 156 |
| T2DM+Myocardial infarction | | | | | | |
| Medication | 29 | 3 | 1,099 | 29 | 4 | 1,359 |
| Procedure | 28 | 3 | 1,116 | 29 | 3 | 5,722 |
| Consultation | 29 | 3 | 156 | 27 | 3 | 168 |
| Consumables | 3 | 1 | 575 | 1 | 3 | 143 |
| Services | 4 | 1 | 332 | 3 | 2 | 1,913 |
| Others | 5 | 1 | 174 | 5 | 1 | 318 |
| T2DM+Other Cardiovascular Disease | | | | | | |
| Medication | 9 | 4 | 1,765 | 12 | 3 | 1,060 |
| Procedure | 10 | 3 | 1,342 | 9 | 3 | 1,475 |
| Consultation | 9 | 4 | 140 | 10 | 3 | 120 |
| Consumables | 1 | 1 | 84 |  |  |  |
| Services |  |  |  |  |  |  |
| Others | 1 | 1 | 364 |  |  |  |
| T2DM+Periphery vascular disease | | | | | | |
| Medication | 6 | 5 | 3,644 | 6 | 6 | 5,441 |
| Procedure | 7 | 4 | 2,632 | 6 | 5 | 3,600 |
| Consultation | 7 | 3 | 269 | 5 | 5 | 460 |
| Consumables | 1 | 2 | 561 | 2 | 2 | 352 |
| Services |  |  |  | 1 | 2 | 200 |
| Others | 1 | 1 | 355 |  |  |  |
| Medication | 235 | 4 | 1,780 | 228 | 4 | 2,500 |
| Procedure | 213 | 3 | 1,242 | 213 | 4 | 3,461 |
| Consultation | 221 | 4 | 247 | 221 | 4 | 479 |
| Consumables | 28 | 2 | 270 | 63 | 2 | 981 |
| Services | 20 | 2 | 880 | 37 | 3 | 8,558 |
| Others | 43 | 2 | 507 | 66 | 2 | 843 |
| **T2DM With Multiple CVD** | | | | | | |
| T2DM+Coronary Arterial Revascularization+Coronary Artery Disease | | | | | | |
| Medication | 23 | 5 | 1,669 | 21 | 6 | 3,680 |
| Procedure | 17 | 4 | 1,091 | 19 | 4 | 11,086 |
| Consultation | 18 | 6 | 309 | 22 | 5 | 448 |
| Consumables | 1 | 7 | 525 | 5 | 2 | 1,404 |
| Services | 1 | 1 | 370 | 4 | 6 | 6,110 |
| Others | 6 | 3 | 302 | 2 | 2 | 777 |
| T2DM+Coronary Artery Disease+Angina | | | | | | |
| Medication | 133 | 4 | 2,065 | 148 | 6 | 3,516 |
| Procedure | 131 | 3 | 1,213 | 142 | 4 | 9,952 |
| Consultation | 129 | 4 | 250 | 147 | 5 | 436 |
| Consumables | 18 | 2 | 460 | 46 | 2 | 8,083 |
| Services | 10 | 1 | 219 | 45 | 1 | 3,026 |
| Others | 24 | 2 | 548 | 42 | 2 | 758 |
| T2DM+Coronary Artery Disease+Atrial fibrillation | | | | | | |
| Medication | 41 | 5 | 2,440 | 47 | 6 | 3,678 |
| Procedure | 37 | 3 | 1,181 | 43 | 3 | 4,314 |
| Consultation | 38 | 4 | 317 | 46 | 5 | 507 |
| Consumables | 8 | 2 | 307 | 12 | 1 | 701 |
| Services | 2 | 1 | 55 | 11 | 1 | 1,527 |
| Others | 10 | 2 | 292 | 14 | 2 | 1,279 |
| T2DM+Coronary Artery Disease+Chronic renal failure | | | | | | |
| Medication | 33 | 5 | 3,056 | 40 | 6 | 4,706 |
| Procedure | 30 | 4 | 1,609 | 38 | 4 | 5,253 |
| Consultation | 30 | 5 | 429 | 38 | 5 | 782 |
| Consumables | 10 | 2 | 207 | 14 | 2 | 513 |
| Services | 3 | 1 | 162 | 10 | 1 | 4,817 |
| Others | 4 | 2 | 242 | 10 | 2 | 589 |
| T2DM+Heart Failure+Coronary Artery Disease**7** | | | | | | |
| Medication | 56 | 4 | 2,506 | 65 | 6 | 4,830 |
| Procedure | 42 | 3 | 1,913 | 61 | 4 | 6,251 |
| Consultation | 50 | 4 | 327 | 63 | 6 | 698 |
| Consumables | 11 | 2 | 253 | 18 | 2 | 6,424 |
| Services | 4 | 1 | 200 | 12 | 2 | 12,164 |
| Others | 12 | 2 | 327 | 19 | 2 | 1,531 |
| T2DM+Myocardial infarction+Coronary Artery Disease | | | | | | |
| Medication | 126 | 4 | 1,749 | 156 | 7 | 4,641 |
| Procedure | 119 | 3 | 1,048 | 147 | 4 | 11,105 |
| Consultation | 123 | 4 | 190 | 153 | 6 | 574 |
| Consumables | 15 | 3 | 211 | 58 | 2 | 8,420 |
| Services | 8 | 2 | 148 | 41 | 1 | 5,448 |
| Others | 17 | 2 | 475 | 50 | 2 | 3,100 |
| T2DM+Myocardial infarction+Coronary Artery Disease+Angina | | | | | | |
| Medication | 43 | 4 | 2,567 | 50 | 7 | 5,373 |
| Procedure | 37 | 4 | 1,544 | 50 | 4 | 15,698 |
| Consultation | 35 | 4 | 218 | 50 | 6 | 730 |
| Consumables | 8 | 2 | 470 | 27 | 2 | 7,727 |
| Services | 3 | 1 | 63 | 20 | 2 | 5,852 |
| Others | 5 | 3 | 528 | 13 | 3 | 3,761 |
| T2DM+Stroke or TIA+Coronary Artery Disease**683** | | | | | | |
| Medication | 110 | 4 | 2,398 | 132 | 6 | 4,110 |
| Procedure | 101 | 3 | 1,154 | 122 | 4 | 5,199 |
| Consultation | 111 | 4 | 312 | 129 | 5 | 781 |
| Consumables | 22 | 2 | 839 | 42 | 2 | 664 |
| Services | 12 | 1 | 666 | 35 | 4 | 7,374 |
| Others | 25 | 2 | 676 | 45 | 2 | 556 |
| Abbreviations: CVD=Cardiovascular disease, HCRU=Healthcare cost utilization, N=Number of patients, T2DM=Type 2 diabetes mellitus, TIA=Transient ischemic attack | | | | | | |
