# Supplementary material for: Cost of cardiovascular diseases and renal complications in people with type 2 diabetes mellitus in the Kingdom of Saudi Arabia: A retrospective analysis of claims database
Source: PLoS One. 2022 Oct 20;17(10):e0273836. doi: 10.1371/journal.pone.0273836 (PMC9584438; doi:10.1371/journal.pone.0273836)
Supplement: S16 Table — (DOCX) [file pone.0273836.s016.docx]

### S16 Table: Comparison of pre-index and post-index disease-specific cause cost for various activities (Payer 1, Cohort 2)

|  |  | | | | | | | | |
| --- | --- | --- | --- | --- | --- | --- | --- | --- | --- |
|  | **Pre-Index 1 Yr** |  |  | **Post-Index 1 Yr** |  |  | **Post-Index 2 Yr** |  |  |
| **Payer 1** | **Disease-specfic Cause** | | | **Disease-specific Cause** | | | **Disease-specific Cause** | | |
| **Cohort 2** | **N** | **HCRU** | **Cost** | **N** | **HCRU** | **Cost** | **N** | **HCRU** | **Cost** |
| **T2DM With One CVD** | | | | | | | | | |
| T2DM+Angina | | | | | | | | | |
| Medication | 57 | 4 | 1,640 | 47 | 5 | 2,475 | 54 | 4 | 2,182 |
| Procedure | 57 | 3 | 931 | 47 | 3 | 1,323 | 46 | 4 | 1,272 |
| Consultation | 59 | 4 | 145 | 48 | 4 | 186 | 48 | 4 | 160 |
| Consumables | 8 | 3 | 197 | 9 | 3 | 292 | 10 | 1 | 293 |
| Services | 6 | 1 | 84 | 4 | 2 | 168 | 2 | 1 | 311 |
| Others | 6 | 1 | 213 | 7 | 2 | 388 | 4 | 2 | 509 |
| T2DM+Atrial fibrillation | | | | | | | | | |
| Medication | 13 | 3 | 1,365 | 12 | 5 | 2,285 | 13 | 4 | 2,196 |
| Procedure | 9 | 2 | 992 | 11 | 2 | 1,105 | 13 | 2 | 1,368 |
| Consultation | 14 | 2 | 177 | 12 | 4 | 268 | 12 | 5 | 320 |
| Consumables |  |  |  | 1 | 1 | 60 | 1 | 1 | 10 |
| Services | 2 | 2 | 150 | 1 | 1 | 100 |  |  |  |
| Others | 1 | 1 | 154 | 1 | 1 | 121 | 4 | 1 | 101 |
| T2DM+Chronic renal failure | | | | | | | | | |
| Medication | 41 | 5 | 3,300 | 40 | 6 | 3,902 | 37 | 9 | 6,791 |
| Procedure | 40 | 4 | 1,579 | 35 | 5 | 8,076 | 36 | 8 | 13,122 |
| Consultation | 38 | 4 | 391 | 40 | 4 | 611 | 36 | 6 | 857 |
| Consumables | 5 | 2 | 269 | 10 | 4 | 835 | 8 | 5 | 4,718 |
| Services | 3 | 2 | 167 | 7 | 2 | 2,590 | 11 | 2 | 1,965 |
| Others | 5 | 2 | 465 | 9 | 3 | 467 | 12 | 2 | 1,358 |
| T2DM+Coronary Artery Disease | | | | | | | | | |
| Medication | 667 | 5 | 2,397 | 678 | 5 | 3,306 | 662 | 5 | 3,676 |
| Procedure | 589 | 3 | 1,486 | 615 | 3 | 2,462 | 605 | 4 | 2,254 |
| Consultation | 601 | 4 | 314 | 640 | 4 | 366 | 627 | 4 | 359 |
| Consumables | 68 | 2 | 252 | 112 | 2 | 792 | 125 | 2 | 667 |
| Services | 64 | 2 | 347 | 77 | 1 | 656 | 69 | 2 | 374 |
| Others | 129 | 2 | 353 | 174 | 2 | 530 | 128 | 2 | 580 |
| T2DM+Dysrhythmia | | | | | | | | | |
| Medication | 5 | 5 | 1,389 | 4 | 9 | 2,425 | 4 | 3 | 1,469 |
| Procedure | 4 | 4 | 1,067 | 4 | 4 | 1,209 | 3 | 4 | 1,467 |
| Consultation | 4 | 5 | 260 | 4 | 6 | 206 | 4 | 3 | 215 |
| Consumables | 1 | 5 | 725 | 1 | 3 | 247 | 1 | 1 | 90 |
| Services | 1 | 1 | 2,368 | 1 | 1 | 16 |  |  |  |
| Others | 1 | 1 | 44 |  |  |  |  |  |  |
| T2DM+Heart Failure | | | | | | | | | |
| Medication | 17 | 6 | 2,212 | 19 | 5 | 2,300 | 18 | 5 | 3,022 |
| Procedure | 17 | 4 | 2,189 | 15 | 3 | 2,413 | 16 | 4 | 2,202 |
| Consultation | 17 | 5 | 459 | 15 | 5 | 608 | 17 | 5 | 436 |
| Consumables | 3 | 1 | 179 | 2 | 2 | 213 | 5 | 1 | 333 |
| Services | 4 | 2 | 339 | 2 | 1 | 100 | 2 | 2 | 200 |
| Others | 8 | 1 | 265 | 3 | 2 | 153 | 4 | 2 | 1,069 |
| T2DM+Myocardial infarction**6** | | | | | | | | | |
| Medication | 5 | 3 | 1,989 | 5 | 2 | 418 | 3 | 5 | 2,785 |
| Procedure | 5 | 3 | 2,429 | 5 | 3 | 4,560 | 2 | 9 | 6,752 |
| Consultation | 4 | 3 | 338 | 5 | 2 | 106 | 4 | 4 | 381 |
| Consumables |  |  |  |  |  |  | 1 | 2 | 253 |
| Services | 3 | 1 | 402 | 1 | 3 | 300 |  |  |  |
| Others | 1 | 1 | 72 | 1 | 1 | 551 | 3 | 1 | 165 |
| T2DM+Other Cardiovascular Disease | | | | | | | | | |
| Medication | 5 | 4 | 1,612 | 6 | 5 | 1,439 | 6 | 4 | 1,661 |
| Procedure | 5 | 3 | 1,069 | 5 | 3 | 1,236 | 6 | 4 | 1,181 |
| Consultation | 4 | 5 | 119 | 5 | 4 | 174 | 5 | 3 | 69 |
| Consumables |  |  |  |  |  |  |  |  |  |
| Services |  |  |  |  |  |  |  |  |  |
| Others |  |  |  |  |  |  |  |  |  |
| T2DM+Periphery vascular disease | | | | | | | | | |
| Medication | 1 | 7 | 7,498 | 1 | 3 | 5,792 | 1 | 3 | 7,853 |
| Procedure | 1 | 6 | 5,050 | 1 | 3 | 1,365 | 1 | 3 | 1,647 |
| Consultation | 1 | 3 | 280 |  |  |  |  |  |  |
| Consumables | 1 | 2 | 561 | 1 | 3 | 693 | 1 | 3 | 840 |
| Services |  |  |  |  |  |  |  |  |  |
| Others |  |  |  |  |  |  |  |  |  |
| T2DM+Stroke or TIA**09** | | | | | | | | | |
| Medication | 79 | 4 | 1,711 | 79 | 4 | 2,614 | 73 | 4 | 2,222 |
| Procedure | 73 | 3 | 1,400 | 70 | 3 | 3,923 | 72 | 4 | 1,881 |
| Consultation | 72 | 4 | 295 | 71 | 4 | 478 | 71 | 4 | 418 |
| Consumables | 10 | 2 | 244 | 17 | 2 | 737 | 14 | 3 | 261 |
| Services | 7 | 1 | 405 | 12 | 1 | 10,221 | 4 | 2 | 189 |
| Others | 15 | 1 | 221 | 23 | 2 | 730 | 16 | 2 | 738 |
| **T2DM With Multiple CVD** | | | | | | | | | |
| T2DM+Coronary Arterial Revascularization+Coronary Artery Disease | | | | | | | | | |
| Medication | 10 | 6 | 2,531 | 9 | 6 | 4,625 | 9 | 5 | 3,529 |
| Procedure | 8 | 4 | 1,078 | 9 | 4 | 3,172 | 8 | 4 | 1,633 |
| Consultation | 8 | 6 | 443 | 9 | 5 | 578 | 9 | 6 | 499 |
| Consumables | 1 | 7 | 525 | 1 | 6 | 450 |  |  |  |
| Services | 1 | 1 | 370 | 2 | 11 | 5,998 | 2 | 2 | 564 |
| Others | 2 | 2 | 173 | 1 | 2 | 555 | 1 | 2 | 1,262 |
| T2DM+Coronary Artery Disease+Angina | | | | | | | | | |
| Medication | 56 | 5 | 2,541 | 61 | 5 | 3,732 | 58 | 5 | 3,630 |
| Procedure | 53 | 3 | 1,318 | 62 | 3 | 9,012 | 51 | 4 | 4,490 |
| Consultation | 53 | 4 | 274 | 63 | 4 | 416 | 57 | 4 | 388 |
| Consumables | 9 | 2 | 578 | 17 | 2 | 5,232 | 12 | 2 | 3,099 |
| Services | 2 | 2 | 150 | 20 | 1 | 1,678 | 7 | 1 | 1,522 |
| Others | 9 | 1 | 658 | 19 | 2 | 613 | 11 | 2 | 2,130 |
| T2DM+Coronary Artery Disease+Atrial fibrillation | | | | | | | | | |
| Medication | 17 | 4 | 1,751 | 19 | 5 | 3,160 | 19 | 4 | 2,737 |
| Procedure | 14 | 2 | 726 | 17 | 3 | 2,942 | 16 | 3 | 2,088 |
| Consultation | 16 | 4 | 198 | 18 | 5 | 387 | 18 | 4 | 362 |
| Consumables | 2 | 2 | 123 | 5 | 1 | 369 | 2 | 1 | 110 |
| Services |  |  |  | 4 | 2 | 1,153 | 2 | 2 | 4,300 |
| Others | 3 | 2 | 35 | 4 | 2 | 384 | 5 | 1 | 1,656 |
| T2DM+Coronary Artery Disease+Chronic renal failure | | | | | | | | | |
| Medication | 14 | 4 | 2,596 | 18 | 5 | 3,325 | 19 | 5 | 4,700 |
| Procedure | 13 | 4 | 1,466 | 17 | 3 | 2,881 | 18 | 4 | 6,474 |
| Consultation | 12 | 3 | 304 | 17 | 4 | 582 | 18 | 5 | 669 |
| Consumables | 4 | 3 | 273 | 6 | 2 | 466 | 7 | 3 | 709 |
| Services | 1 | 2 | 285 | 4 | 1 | 2,945 | 1 | 1 | 2,100 |
| Others | 1 | 1 | 500 | 3 | 3 | 744 | 2 | 1 | 196 |
| T2DM+Heart Failure+Coronary Artery Disease | | | | | | | | | |
| Medication | 28 | 4 | 2,046 | 32 | 7 | 3,938 | 28 | 5 | 4,309 |
| Procedure | 22 | 3 | 1,483 | 29 | 4 | 3,329 | 24 | 3 | 5,198 |
| Consultation | 25 | 3 | 247 | 31 | 6 | 501 | 28 | 4 | 483 |
| Consumables | 5 | 1 | 193 | 9 | 2 | 369 | 10 | 2 | 1,727 |
| Services | 4 | 1 | 200 | 4 | 2 | 1,176 | 5 | 2 | 4,375 |
| Others | 6 | 2 | 337 | 10 | 3 | 743 | 5 | 1 | 324 |
| T2DM+Myocardial infarction+Coronary Artery Disease | | | | | | | | | |
| Medication | 44 | 4 | 1,901 | 55 | 7 | 4,791 | 52 | 6 | 4,325 |
| Procedure | 38 | 3 | 1,235 | 51 | 4 | 8,890 | 43 | 4 | 3,435 |
| Consultation | 40 | 3 | 179 | 53 | 6 | 570 | 47 | 6 | 464 |
| Consumables | 4 | 2 | 219 | 17 | 2 | 9,499 | 10 | 2 | 1,129 |
| Services | 1 | 2 | 200 | 14 | 1 | 9,116 | 8 | 1 | 1,065 |
| Others | 5 | 2 | 493 | 16 | 2 | 1,253 | 11 | 2 | 756 |
| T2DM+Myocardial infarction+Coronary Artery Disease+Angina | | | | | | | | | |
| Medication | 20 | 4 | 3,024 | 22 | 6 | 5,433 | 20 | 8 | 5,824 |
| Procedure | 18 | 4 | 1,622 | 21 | 4 | 8,590 | 20 | 5 | 7,374 |
| Consultation | 15 | 4 | 208 | 21 | 5 | 443 | 20 | 8 | 597 |
| Consumables | 5 | 2 | 571 | 10 | 2 | 3,368 | 9 | 2 | 10,062 |
| Services |  |  |  | 7 | 1 | 1,599 | 8 | 2 | 3,049 |
| Others | 3 | 4 | 340 | 3 | 3 | 11,055 | 4 | 3 | 13,591 |
| T2DM+Stroke or TIA+Coronary Artery Disease | | | | | | | | | |
| Medication | 59 | 4 | 2,610 | 68 | 6 | 3,945 | 67 | 5 | 4,341 |
| Procedure | 53 | 3 | 1,183 | 60 | 4 | 4,951 | 59 | 5 | 6,439 |
| Consultation | 58 | 3 | 298 | 66 | 5 | 723 | 68 | 4 | 656 |
| Consumables | 13 | 3 | 1,228 | 20 | 2 | 658 | 14 | 2 | 4,498 |
| Services | 8 | 2 | 158 | 18 | 2 | 8,543 | 9 | 2 | 12,167 |
| Others | 14 | 2 | 243 | 20 | 2 | 337 | 19 | 2 | 1,761 |
| Abbreviations: CVD=Cardiovascular disease, HCRU=Healthcare cost utilization, N=Number of patients, T2DM=Type 2 diabetes mellitus, TIA=Transient ischemic attack | | | | | | | | | |
