# Supplementary material for: Cost of cardiovascular diseases and renal complications in people with type 2 diabetes mellitus in the Kingdom of Saudi Arabia: A retrospective analysis of claims database
Source: PLoS One. 2022 Oct 20;17(10):e0273836. doi: 10.1371/journal.pone.0273836 (PMC9584438; doi:10.1371/journal.pone.0273836)
Supplement: S17 Table — (DOCX) [file pone.0273836.s017.docx]

### S17 Table: Comparison of pre-index and post-index disease-specific cause cost for various activities (Payer 1, Cohort 3)

| **Disease-specific Cause** | **Pre-Index 1 Yr** | | | **Post-Index 1 Yr** | | | **Post-Index 2 Yr** | | | **Post-Index 3 Yr** | | |  |
| --- | --- | --- | --- | --- | --- | --- | --- | --- | --- | --- | --- | --- | --- |
| **Payer 1** |  |  |  |  |  |  |  |  |  |  |  |  |  |
| **Cohort 3** | **N** | **HCRU** | **Cost** | **N** | **HCRU** | **Cost** | **N** | **HCRU** | **Cost** | **N** | **HCRU** | **Cost** |  |
| **T2DM WITH ONE CVD** | | | | | | | | | | | | |  |
| **T2DM+Angina** | **25** | **15** | **3,219** | **19** | **21** | **4,170** | **16** | **16** | **3,807** | **20** | **13** | **4,063** |  |
| Medication | 7 | 4 | 1,825 | 5 | 6 | 2,529 | 6 | 4 | 2,026 | 7 | 3 | 2,297 |  |
| Procedure | 7 | 4 | 1,028 | 5 | 4 | 1,014 | 4 | 7 | 1,591 | 6 | 4 | 1,052 |  |
| Consultation | 7 | 4 | 56 | 6 | 5 | 97 | 5 | 5 | 105 | 6 | 3 | 70 |  |
| Consumables | 1 | 1 | 75 | 1 | 2 | 150 | 1 | 1 | 84 | 1 | 4 | 643 |  |
| Services | 2 | 1 | 73 | 1 | 2 | 200 |  |  |  |  |  |  |  |
| Others | 1 | 1 | 162 | 1 | 2 | 180 |  |  |  |  |  |  |  |
| **T2DM+Atrial fibrillation** | **6** | **5** | **1,806** | **4** | **9** | **2,874** | **4** | **15** | **5,206** | **4** | **17** | **8,220** |  |
| Medication | 2 | 2 | 904 | 1 | 3 | 394 | 1 | 2 | 2,034 | 1 | 7 | 5,992 |  |
| Procedure | 1 | 1 | 595 | 1 | 2 | 1,921 | 2 | 3 | 1,711 | 1 | 3 | 1,313 |  |
| Consultation | 2 | 1 | 206 | 1 | 3 | 459 | 1 | 10 | 1,460 | 1 | 6 | 815 |  |
| Consumables |  |  |  |  |  |  |  |  |  |  |  |  |  |
| Services | 1 | 1 | 100 | 1 | 1 | 100 |  |  |  | 1 | 1 | 100 |  |
| Others |  |  |  |  |  |  |  |  |  |  |  |  |  |
| **T2DM+Chronic renal failure** | **26** | **18** | **4,996** | **27** | **41** | **17,116** | **27** | **44** | **15,138** | **20** | **41** | **10,523** |  |
| Medication | 8 | 5 | 1,841 | 7 | 9 | 3,414 | 6 | 16 | 7,573 | 6 | 12 | 3,769 |  |
| Procedure | 8 | 3 | 1,245 | 7 | 6 | 7,891 | 7 | 10 | 5,380 | 6 | 10 | 4,939 |  |
| Consultation | 6 | 4 | 834 | 7 | 4 | 1,131 | 7 | 3 | 525 | 5 | 3 | 458 |  |
| Consumables | 1 | 3 | 439 | 2 | 10 | 354 | 2 | 10 | 370 | 2 | 10 | 338 |  |
| Services | 2 | 2 | 150 | 2 | 4 | 2,927 | 3 | 1 | 568 |  |  |  |  |
| Others | 1 | 2 | 487 | 2 | 8 | 1,400 | 2 | 4 | 723 | 1 | 6 | 1,020 |  |
| **T2DM+Coronary Artery Disease** | **503** | **17** | **5,107** | **546** | **18** | **7,798** | **526** | **19** | **7,431** | **481** | **17** | **9,506** |  |
| Medication | 159 | 5 | 2,205 | 159 | 5 | 3,499 | 157 | 5 | 3,809 | 141 | 4 | 3,333 |  |
| Procedure | 142 | 3 | 1,420 | 145 | 4 | 2,599 | 139 | 4 | 2,188 | 135 | 3 | 2,678 |  |
| Consultation | 142 | 4 | 329 | 151 | 4 | 364 | 150 | 5 | 394 | 136 | 4 | 327 |  |
| Consumables | 10 | 2 | 291 | 26 | 2 | 545 | 33 | 3 | 355 | 33 | 2 | 1,499 |  |
| Services | 19 | 2 | 632 | 20 | 1 | 274 | 19 | 1 | 128 | 20 | 1 | 368 |  |
| Others | 31 | 1 | 231 | 45 | 2 | 518 | 28 | 2 | 557 | 16 | 2 | 1,301 |  |
| **T2DM+Dysrhythmia** | **1** | **1** | **912** | **3** | **7** | **4,357** | **3** | **9** | **5,830** | **3** | **5** | **3,906** |  |
| Medication | 1 | 1 | 912 | 1 | 3 | 1,897 | 1 | 2 | 2,406 | 1 | 1 | 1,962 |  |
| Procedure |  |  |  | 1 | 3 | 2,410 | 1 | 5 | 3,319 | 1 | 3 | 1,884 |  |
| Consultation |  |  |  | 1 | 1 | 50 | 1 | 2 | 105 | 1 | 1 | 60 |  |
| Consumables |  |  |  |  |  |  |  |  |  |  |  |  |  |
| Services |  |  |  |  |  |  |  |  |  |  |  |  |  |
| Others |  |  |  |  |  |  |  |  |  |  |  |  |  |
| **T2DM+Heart Failure** | **11** | **18** | **4,692** | **12** | **14** | **5,771** | **15** | **15** | **9,807** | **10** | **20** | **11,163** |  |
| Medication | 3 | 6 | 1,776 | 4 | 5 | 3,387 | 4 | 5 | 6,014 | 3 | 8 | 8,023 |  |
| Procedure | 2 | 5 | 2,260 | 3 | 4 | 1,828 | 4 | 4 | 2,688 | 3 | 4 | 2,292 |  |
| Consultation | 3 | 3 | 358 | 3 | 3 | 305 | 4 | 4 | 526 | 3 | 6 | 680 |  |
| Consumables | 1 | 1 | 8 | 1 | 2 | 150 | 2 | 2 | 119 | 1 | 2 | 168 |  |
| Services | 1 | 2 | 200 | 1 | 1 | 100 |  |  |  |  |  |  |  |
| Others | 1 | 1 | 90 |  |  |  | 1 | 1 | 460 |  |  |  |  |
| **T2DM+Stroke or TIA** | **45** | **16** | **3,639** | **44** | **16** | **6,430** | **38** | **20** | **6,011** | **43** | **17** | **4,962** |  |
| Medication | 12 | 4 | 1,426 | 14 | 4 | 1,954 | 13 | 5 | 1,785 | 13 | 4 | 1,881 |  |
| Procedure | 14 | 4 | 1,468 | 12 | 4 | 2,406 | 11 | 5 | 2,321 | 13 | 4 | 2,349 |  |
| Consultation | 13 | 4 | 215 | 11 | 4 | 250 | 10 | 5 | 454 | 11 | 4 | 344 |  |
| Consumables | 1 | 1 | 75 | 2 | 1 | 75 | 1 | 3 | 225 | 2 | 3 | 79 |  |
| Services | 3 | 1 | 133 | 2 | 2 | 150 | 1 | 1 | 100 | 3 | 2 | 304 |  |
| Others | 2 | 2 | 322 | 3 | 2 | 1,594 | 2 | 1 | 1,126 | 1 | 1 | 5 |  |
| **T2DM WITH MULTIPLE CVD** | | | | | | | | | | | | |  |
| **T2DM+Coronary Arterial Revascularization+Coronary Artery Disease** | **8** | **8** | **2,498** | **6** | **9** | **3,724** | **8** | **10** | **2,342** | **10** | **10** | **24,004** |  |
| Medication | 3 | 2 | 1,425 | 2 | 5 | 2,960 | 3 | 3 | 1,121 | 3 | 2 | 1,036 |  |
| Procedure | 2 | 2 | 638 | 2 | 2 | 694 | 2 | 2 | 1,006 | 3 | 3 | 1,018 |  |
| Consultation | 2 | 3 | 65 | 2 | 2 | 70 | 3 | 4 | 215 | 3 | 4 | 250 |  |
| Consumables |  |  |  |  |  |  |  |  |  |  |  |  |  |
| Services | 1 | 1 | 370 |  |  |  |  |  |  |  |  |  |  |
| Others |  |  |  |  |  |  |  |  |  | 1 | 1 | 21,700 |  |
| **T2DM+Coronary Artery Disease+Angina** | **37** | **16** | **5,187** | **57** | **19** | **24,068** | **48** | **18** | **14,987** | **48** | **17** | **9,075** |  |
| Medication | 13 | 4 | 2,181 | 15 | 5 | 2,881 | 15 | 5 | 3,863 | 15 | 5 | 3,396 |  |
| Procedure | 11 | 3 | 1,065 | 15 | 4 | 5,448 | 14 | 4 | 7,955 | 13 | 4 | 2,217 |  |
| Consultation | 11 | 4 | 335 | 14 | 5 | 401 | 13 | 5 | 443 | 15 | 4 | 330 |  |
| Consumables | 1 | 5 | 1,411 | 2 | 1 | 14,574 | 2 | 1 | 305 | 2 | 1 | 225 |  |
| Services |  |  |  | 5 | 2 | 318 | 2 | 1 | 68 | 2 | 2 | 130 |  |
| Others | 1 | 1 | 195 | 6 | 2 | 445 | 2 | 2 | 2,353 | 1 | 1 | 2,777 |  |
| **T2DM+Coronary Artery Disease+Atrial fibrillation** | **21** | **13** | **2,717** | **22** | **22** | **9,118** | **19** | **11** | **6,719** | **20** | **13** | **23,753** |  |
| Medication | 6 | 5 | 1,839 | 6 | 7 | 4,193 | 6 | 4 | 1,947 | 6 | 3 | 3,902 |  |
| Procedure | 6 | 2 | 459 | 6 | 4 | 2,719 | 5 | 2 | 727 | 5 | 3 | 3,937 |  |
| Consultation | 6 | 4 | 233 | 6 | 6 | 441 | 5 | 3 | 335 | 4 | 4 | 520 |  |
| Consumables | 1 | 2 | 156 | 2 | 1 | 804 | 1 | 1 | 80 | 2 | 1 | 14,505 |  |
| Services |  |  |  | 1 | 1 | 155 |  |  |  | 2 | 1 | 870 |  |
| Others | 2 | 1 | 30 | 1 | 3 | 805 | 2 | 1 | 3,630 | 1 | 1 | 19 |  |
| **T2DM+Coronary Artery Disease+Chronic renal failure** | **6** | **10** | **4,913** | **11** | **19** | **5,756** | **11** | **20** | **8,377** | **9** | **8** | **7,080** |  |
| Medication | 2 | 5 | 2,564 | 3 | 7 | 3,112 | 3 | 5 | 4,846 | 3 | 2 | 1,516 |  |
| Procedure | 2 | 3 | 1,779 | 3 | 4 | 1,507 | 3 | 4 | 2,023 | 2 | 2 | 447 |  |
| Consultation | 2 | 3 | 570 | 3 | 7 | 833 | 3 | 7 | 1,041 | 3 | 3 | 285 |  |
| Consumables |  |  |  | 1 | 1 | 5 | 1 | 3 | 239 |  |  |  |  |
| Services |  |  |  | 1 | 1 | 300 |  |  |  |  |  |  |  |
| Others |  |  |  |  |  |  | 1 | 1 | 228 | 1 | 1 | 4,832 |  |
| **T2DM+Heart Failure+Chronic renal failure** | **7** | **6** | **2,827** | **13** | **16** | **15,652** | **13** | **11** | **20,459** | **7** | **5** | **3,642** |  |
| Medication | 2 | 3 | 1,193 | 3 | 4 | 4,049 | 3 | 3 | 3,186 | 2 | 3 | 2,481 |  |
| Procedure | 2 | 2 | 1,370 | 3 | 4 | 5,978 | 3 | 3 | 7,928 | 3 | 1 | 841 |  |
| Consultation | 3 | 2 | 263 | 3 | 2 | 950 | 3 | 2 | 1,043 | 2 | 2 | 320 |  |
| Consumables |  |  |  |  |  |  | 1 | 1 | 3,557 |  |  |  |  |
| Services |  |  |  | 2 | 1 | 4,090 | 2 | 1 | 4,247 |  |  |  |  |
| Others |  |  |  | 2 | 5 | 585 | 1 | 1 | 497 |  |  |  |  |
| **T2DM+Heart Failure+Coronary Artery Disease** | **16** | **12** | **7,490** | **23** | **27** | **14,448** | **20** | **15** | **7,714** | **19** | **15** | **8,889** |  |
| Medication | 5 | 3 | 3,108 | 6 | 8 | 6,620 | 6 | 6 | 5,252 | 6 | 5 | 4,034 |  |
| Procedure | 3 | 2 | 3,241 | 6 | 4 | 3,978 | 5 | 3 | 1,498 | 5 | 3 | 2,917 |  |
| Consultation | 3 | 2 | 363 | 6 | 8 | 1,039 | 6 | 5 | 609 | 6 | 3 | 408 |  |
| Consumables | 2 | 2 | 342 | 1 | 4 | 888 | 2 | 2 | 206 | 1 | 3 | 730 |  |
| Services | 2 | 2 | 235 | 1 | 2 | 1,774 | 1 | 1 | 150 |  |  |  |  |
| Others | 1 | 1 | 200 | 3 | 1 | 148 |  |  |  | 1 | 1 | 801 |  |
| **T2DM+Myocardial infarction+Coronary Artery Disease** | **24** | **15** | **4,195** | **38** | **23** | **46,714** | **37** | **27** | **13,258** | **33** | **20** | **9,538** |  |
| Medication | 8 | 5 | 1,835 | 10 | 7 | 5,641 | 10 | 8 | 5,336 | 10 | 6 | 5,188 |  |
| Procedure | 7 | 4 | 1,663 | 10 | 4 | 14,724 | 10 | 5 | 3,785 | 9 | 3 | 2,872 |  |
| Consultation | 8 | 4 | 192 | 10 | 6 | 405 | 10 | 8 | 603 | 10 | 6 | 460 |  |
| Consumables | 1 | 3 | 504 | 2 | 3 | 21,112 | 3 | 2 | 3,268 | 2 | 3 | 373 |  |
| Services |  |  |  | 3 | 1 | 4,435 | 1 | 1 | 100 | 1 | 1 | 600 |  |
| Others |  |  |  | 3 | 2 | 397 | 3 | 3 | 165 | 1 | 1 | 46 |  |
| **T2DM+Myocardial infarction+Coronary Artery Disease+Angina** | **10** | **17** | **11,503** | **12** | **14** | **28,611** | **14** | **29** | **17,998** | **14** | **21** | **13,230** |  |
| Medication | 3 | 5 | 7,378 | 3 | 4 | 5,452 | 3 | 7 | 8,382 | 3 | 5 | 7,604 |  |
| Procedure | 3 | 6 | 3,686 | 3 | 4 | 12,784 | 3 | 9 | 4,580 | 3 | 6 | 3,658 |  |
| Consultation | 2 | 5 | 250 | 3 | 3 | 243 | 3 | 9 | 632 | 3 | 6 | 340 |  |
| Consumables | 2 | 2 | 189 | 2 | 2 | 10,033 | 3 | 2 | 339 | 3 | 3 | 328 |  |
| Services |  |  |  | 1 | 1 | 100 | 1 | 1 | 100 | 2 | 2 | 1,300 |  |
| Others |  |  |  |  |  |  | 1 | 1 | 3,965 |  |  |  |  |
| **T2DM+Stroke or TIA+Coronary Artery Disease** | **74** | **17** | **4,382** | **86** | **20** | **16,643** | **79** | **22** | **9,942** | **64** | **21** | **11,908** |  |
| Medication | 22 | 4 | 2,391 | 23 | 5 | 4,140 | 21 | 5 | 4,818 | 18 | 5 | 4,819 |  |
| Procedure | 17 | 3 | 1,177 | 20 | 4 | 6,441 | 20 | 7 | 3,407 | 18 | 5 | 4,157 |  |
| Consultation | 21 | 3 | 220 | 23 | 4 | 502 | 22 | 4 | 587 | 17 | 4 | 586 |  |
| Consumables | 6 | 3 | 234 | 7 | 3 | 641 | 7 | 3 | 245 | 6 | 2 | 174 |  |
| Services | 3 | 2 | 175 | 4 | 2 | 4,668 | 2 | 1 | 366 | 2 | 1 | 763 |  |
| Others | 5 | 2 | 185 | 9 | 3 | 251 | 7 | 2 | 519 | 3 | 3 | 1,407 |  |
| **T2DM+Stroke or TIA+Heart Failure** | **10** | **20** | **3,522** | **11** | **25** | **4,712** | **12** | **27** | **11,709** | **13** | **23** | **13,596** |  |
| Medication | 3 | 7 | 1,081 | 3 | 7 | 1,985 | 3 | 9 | 2,411 | 3 | 7 | 6,211 |  |
| Procedure | 3 | 6 | 1,683 | 3 | 6 | 789 | 3 | 7 | 5,299 | 3 | 7 | 5,423 |  |
| Consultation | 3 | 6 | 470 | 3 | 6 | 488 | 3 | 8 | 656 | 3 | 6 | 508 |  |
| Consumables |  |  |  | 1 | 1 | 84 | 1 | 1 | 45 | 2 | 2 | 89 |  |
| Services |  |  |  |  |  |  | 1 | 1 | 3,043 | 2 | 2 | 1,366 |  |
| Others | 1 | 1 | 288 | 1 | 5 | 1,366 | 1 | 1 | 255 |  |  |  |  |
| Abbreviations: CVD=Cardiovascular disease, HCRU=Healthcare cost utilization, N=Number of patients, T2DM=Type 2 diabetes mellitus, TIA=Transient ischemic attack | | | | | | | | | | | | | |
