# Supplementary material for: Cost of cardiovascular diseases and renal complications in people with type 2 diabetes mellitus in the Kingdom of Saudi Arabia: A retrospective analysis of claims database
Source: PLoS One. 2022 Oct 20;17(10):e0273836. doi: 10.1371/journal.pone.0273836 (PMC9584438; doi:10.1371/journal.pone.0273836)
Supplement: S18 Table — (DOCX) [file pone.0273836.s018.docx]

### S18 Table: Comparison of pre-index and post-index disease-specific cause cost for various activities (Payer 2, Cohort 1)

| **Disease-specific Cause** | **Pre-Index 1 Yr** | | | **Post-Index 1 Yr** | | |
| --- | --- | --- | --- | --- | --- | --- |
| **Payer 2** | **Disease-specific Cause** | | | **Disease-specific Cause** | | |
| **Cohort 1** | **N** | **HCRU** | **Cost** | **N** | **HCRU** | **Cost** |
| **T2DM With One CVD**163,928 | | | | | | |
| T2DM+Angina | | | | | | |
| Medication | 110 | 3 | 1,533 | 90 | 4 | 2,065 |
| Procedure | 98 | 2 | 811 | 88 | 3 | 1,236 |
| Consultation | 116 | 3 | 244 | 81 | 4 | 306 |
| Consumables |  |  |  | 2 | 1 | 16 |
| Services | 18 | 2 | 183 | 18 | 2 | 547 |
| Others | 1 | 1 | 20 |  |  |  |
| T2DM+Atrial fibrillation | | | | | | |
| Medication | 24 | 2 | 1,219 | 23 | 4 | 2,278 |
| Procedure | 17 | 2 | 1,666 | 17 | 3 | 5,712 |
| Consultation | 20 | 3 | 347 | 21 | 4 | 471 |
| Consumables | 1 | 1 | 998 | 1 | 1 | 0 |
| Services | 8 | 1 | 472 | 6 | 2 | 227 |
| Others |  |  |  |  |  |  |
| T2DM+Chronic renal failure | | | | | | |
| Medication | 92 | 5 | 3,032 | 95 | 7 | 5,569 |
| Procedure | 82 | 3 | 1,969 | 86 | 6 | 13,531 |
| Consultation | 92 | 5 | 427 | 94 | 6 | 733 |
| Consumables | 2 | 1 | 94 | 2 | 1 | 75 |
| Services | 24 | 2 | 1,271 | 40 | 3 | 4,876 |
| Others | 2 | 3 | 691 | 1 | 1 | 177 |
| T2DM+Coronary Arterial Revascularization | | | | | | |
| Medication | 2 | 4 | 1,205 | 1 | 5 | 3,450 |
| Procedure | 2 | 3 | 683 | 2 | 2 | 265 |
| Consultation | 2 | 3 | 93 | 1 | 4 | 100 |
| Consumables |  |  |  |  |  |  |
| Services | 1 | 1 | 90 |  |  |  |
| Others |  |  |  |  |  |  |
| T2DM+Coronary Artery Disease18 | | | | | | |
| Medication | 678 | 4 | 2,336 | 658 | 5 | 3,209 |
| Procedure | 538 | 3 | 1,121 | 546 | 3 | 6,238 |
| Consultation | 692 | 4 | 308 | 629 | 5 | 424 |
| Consumables | 3 | 3 | 229 | 17 | 2 | 1,233 |
| Services | 147 | 1 | 403 | 195 | 2 | 2,243 |
| Others | 12 | 2 | 441 | 3 | 2 | 572 |
| T2DM+Dysrhythmia | | | | | | |
| Medication | 9 | 5 | 2,044 | 6 | 4 | 1,788 |
| Procedure | 10 | 3 | 1,687 | 7 | 3 | 10,057 |
| Consultation | 10 | 4 | 430 | 7 | 2 | 89 |
| Consumables |  |  |  |  |  |  |
| Services | 3 | 1 | 879 | 3 | 1 | 162 |
| Others |  |  |  |  |  |  |
| T2DM+Heart Failure | | | | | | |
| Medication | 28 | 4 | 2,378 | 27 | 4 | 2,582 |
| Procedure | 23 | 2 | 1,071 | 18 | 3 | 1,771 |
| Consultation | 29 | 3 | 290 | 24 | 4 | 524 |
| Consumables |  |  |  |  |  |  |
| Services | 6 | 2 | 699 | 10 | 2 | 533 |
| Others | 1 | 1 | 100 |  |  |  |
| T2DM+Myocardial infarction1 | | | | | | |
| Medication | 21 | 4 | 1,091 | 18 | 4 | 1,987 |
| Procedure | 15 | 3 | 483 | 15 | 3 | 5,440 |
| Consultation | 20 | 4 | 243 | 17 | 5 | 371 |
| Consumables |  |  |  |  |  |  |
| Services | 4 | 2 | 72 | 7 | 1 | 3,732 |
| Others |  |  |  |  |  |  |
| T2DM+Other Cardiovascular Disease | | | | | | |
| Medication | 11 | 5 | 1,586 | 10 | 3 | 1,140 |
| Procedure | 9 | 3 | 1,734 | 8 | 2 | 8,565 |
| Consultation | 12 | 4 | 422 | 9 | 3 | 465 |
| Consumables |  |  |  | 1 | 1 | 0 |
| Services |  |  |  | 1 | 1 | 15 |
| Others |  |  |  |  |  |  |
| T2DM+Periphery vascular disease | | | | | | |
| Medication | 11 | 4 | 1,887 | 9 | 3 | 2,953 |
| Procedure | 9 | 3 | 2,385 | 6 | 2 | 42,861 |
| Consultation | 11 | 4 | 513 | 9 | 3 | 272 |
| Consumables |  |  |  |  |  |  |
| Services | 5 | 2 | 252 | 4 | 1 | 613 |
| Others |  |  |  |  |  |  |
| T2DM+Stroke or TIA | | | | | | |
| Medication | 163 | 4 | 1,863 | 153 | 5 | 2,794 |
| Procedure | 126 | 2 | 1,367 | 127 | 3 | 3,352 |
| Consultation | 163 | 4 | 284 | 157 | 4 | 1,050 |
| Consumables | 1 | 1 | 4,286 | 11 | 2 | 3,930 |
| Services | 37 | 1 | 1,591 | 57 | 2 | 11,243 |
| Others | 1 | 1 | 110 | 2 | 1 | 86 |
| **T2DM With Multiple CVD** | | | | | | |
| T2DM+Coronary Artery Disease+Angina | | | | | | |
| Medication | 71 | 4 | 1,549 | 81 | 6 | 3,211 |
| Procedure | 54 | 2 | 1,253 | 71 | 3 | 14,100 |
| Consultation | 74 | 3 | 187 | 77 | 5 | 512 |
| Consumables |  |  |  | 3 | 2 | 6,890 |
| Services | 18 | 1 | 500 | 35 | 2 | 4,703 |
| Others | 1 | 1 | 1,107 | 1 | 1 | 523 |
| T2DM+Coronary Artery Disease+Atrial fibrillation | | | | | | |
| Medication | 19 | 5 | 2,335 | 20 | 6 | 4,535 |
| Procedure | 16 | 3 | 1,653 | 18 | 4 | 6,409 |
| Consultation | 19 | 5 | 465 | 20 | 6 | 652 |
| Consumables |  |  |  |  |  |  |
| Services | 4 | 1 | 2,704 | 10 | 1 | 8,790 |
| Others |  |  |  |  |  |  |
| T2DM+Coronary Artery Disease+Chronic renal failure | | | | | | |
| Medication | 15 | 4 | 3,613 | 17 | 7 | 6,279 |
| Procedure | 14 | 3 | 1,699 | 17 | 5 | 15,625 |
| Consultation | 16 | 4 | 532 | 18 | 5 | 863 |
| Consumables |  |  |  | 1 | 2 | 2,230 |
| Services | 4 | 1 | 750 | 9 | 3 | 7,589 |
| Others |  |  |  |  |  |  |
| T2DM+Heart Failure+Coronary Artery Disease | | | | | | |
| Medication | 32 | 5 | 3,005 | 34 | 7 | 7,021 |
| Procedure | 27 | 3 | 2,628 | 30 | 5 | 23,925 |
| Consultation | 32 | 5 | 524 | 33 | 7 | 1,846 |
| Consumables | 1 | 2 | 2,732 | 5 | 1 | 7,840 |
| Services | 9 | 1 | 2,279 | 21 | 2 | 16,498 |
| Others | 1 | 2 | 94 |  |  |  |
| T2DM+Myocardial infarction+Coronary Artery Disease082 | | | | | | |
| Medication | 34 | 4 | 1,852 | 36 | 8 | 5,160 |
| Procedure | 32 | 2 | 856 | 33 | 4 | 29,888 |
| Consultation | 35 | 4 | 265 | 36 | 8 | 779 |
| Consumables | 1 | 2 | 374 | 4 | 1 | 4,640 |
| Services | 10 | 2 | 295 | 24 | 2 | 5,594 |
| Others | 3 | 1 | 214 | 1 | 1 | 20 |
| T2DM+Myocardial infarction+Coronary Artery Disease+Angina | | | | | | |
| Medication | 8 | 4 | 2,992 | 11 | 8 | 4,717 |
| Procedure | 7 | 3 | 1,222 | 11 | 4 | 31,254 |
| Consultation | 9 | 4 | 269 | 11 | 8 | 1,135 |
| Consumables | 1 | 1 | 150 | 1 | 1 | 458 |
| Services | 3 | 1 | 22 | 8 | 2 | 11,505 |
| Others | 1 | 1 | 250 | 1 | 1 | 5 |
| T2DM+Stroke or TIA+Coronary Artery Disease | | | | | | |
| Medication | 35 | 3 | 1,946 | 37 | 6 | 4,517 |
| Procedure | 26 | 3 | 3,224 | 33 | 4 | 9,846 |
| Consultation | 35 | 4 | 471 | 38 | 6 | 1,278 |
| Consumables | 1 | 2 | 785 | 6 | 2 | 2,858 |
| Services | 4 | 2 | 2,945 | 26 | 2 | 5,850 |
| Others | 1 | 2 | 336 |  |  |  |

Abbreviations: CVD=Cardiovascular disease, HCRU=Healthcare cost utilization, N=Number of patients, T2DM=Type 2 diabetes mellitus, TIA=Transient ischemic attack
