# Supplementary material for: Cost of cardiovascular diseases and renal complications in people with type 2 diabetes mellitus in the Kingdom of Saudi Arabia: A retrospective analysis of claims database
Source: PLoS One. 2022 Oct 20;17(10):e0273836. doi: 10.1371/journal.pone.0273836 (PMC9584438; doi:10.1371/journal.pone.0273836)
Supplement: S19 Table — (DOCX) [file pone.0273836.s019.docx]

### S19 Table: Comparison of pre-index and post-index disease-specific cause cost for various activities (Payer 2, Cohort 2)

|  | **Pre-Index 1 Yr** |  |  | **Post-Index 1 Yr** |  |  | **Post-Index 2 Yr** | | |
| --- | --- | --- | --- | --- | --- | --- | --- | --- | --- |
| **Payer 2** | **Disease-specific Cause** | | | **Disease-specific Cause** | | | **Disease-specific Cause** | | |
| **Cohort 2** | **N** | **HCRU** | **Cost** | **N** | **HCRU** | **Cost** | **N** | **HCRU** | **Cost** |
| **T2DM With One CVD28** | | | | | | | | | |
| T2DM+Angina | 104 | 9 | 2,524 | 87 | 14 | 3,671 | 78 | 13 | 2,664 |
| Medication | 36 | 3 | 1,171 | 28 | 4 | 1,773 | 25 | 4 | 1,524 |
| Procedure | 30 | 2 | 691 | 27 | 3 | 1,219 | 24 | 2 | 760 |
| Consultation | 36 | 3 | 232 | 27 | 5 | 331 | 23 | 4 | 216 |
| Consumables |  |  |  |  |  |  | 1 | 1 | 0 |
| Services | 2 | 1 | 430 | 5 | 1 | 348 | 5 | 1 | 163 |
| Others |  |  |  |  |  |  |  |  |  |
| T2DM+Atrial fibrillation | | | | | | | | | |
| Medication | 5 | 3 | 1,494 | 6 | 4 | 2,398 | 4 | 7 | 5,350 |
| Procedure | 4 | 2 | 1,630 | 5 | 3 | 965 | 3 | 4 | 6,401 |
| Consultation | 4 | 5 | 673 | 5 | 5 | 511 | 4 | 5 | 546 |
| Consumables |  |  |  |  |  |  |  |  |  |
| Services | 2 | 1 | 195 | 3 | 2 | 68 | 3 | 1 | 1,750 |
| Others |  |  |  |  |  |  |  |  |  |
| T2DM+Chronic renal failure | | | | | | | | | |
| Medication | 24 | 6 | 2,982 | 24 | 11 | 7,919 | 27 | 6 | 6,013 |
| Procedure | 23 | 4 | 2,062 | 23 | 8 | 18,074 | 25 | 6 | 19,439 |
| Consultation | 25 | 5 | 392 | 24 | 8 | 1,053 | 27 | 4 | 761 |
| Consumables | 1 | 1 | 19 |  |  |  | 2 | 2 | 88 |
| Services | 10 | 1 | 767 | 12 | 4 | 9,183 | 10 | 5 | 15,445 |
| Others |  |  |  |  |  |  |  |  |  |
| T2DM+Coronary Artery Disease | | | | | | | | | |
| Medication | 217 | 4 | 2,112 | 211 | 6 | 3,717 | 197 | 5 | 2,798 |
| Procedure | 176 | 2 | 974 | 185 | 3 | 7,022 | 164 | 3 | 2,845 |
| Consultation | 218 | 4 | 290 | 213 | 6 | 450 | 193 | 5 | 334 |
| Consumables | 1 | 1 | 120 | 5 | 2 | 1,426 | 3 | 2 | 1,125 |
| Services | 44 | 1 | 113 | 61 | 2 | 1,682 | 38 | 2 | 538 |
| Others | 6 | 2 | 183 | 1 | 4 | 912 |  |  |  |
| T2DM+Dysrhythmia | | | | | | | | | |
| Medication | 1 | 1 | 852 |  |  |  | 1 | 7 | 1,368 |
| Procedure | 1 | 2 | 794 | 1 | 2 | 3,555 | 1 | 2 | 185 |
| Consultation | 1 | 1 | 267 |  |  |  | 1 | 3 | 45 |
| Consumables |  |  |  |  |  |  |  |  |  |
| Services |  |  |  |  |  |  | 1 | 1 | 65 |
| Others |  |  |  |  |  |  |  |  |  |
| T2DM+Heart Failure | | | | | | | | | |
| Medication | 12 | 2 | 1,232 | 12 | 4 | 2,849 | 9 | 3 | 3,099 |
| Procedure | 8 | 2 | 484 | 6 | 3 | 1,103 | 6 | 2 | 1,496 |
| Consultation | 11 | 2 | 164 | 10 | 4 | 538 | 8 | 3 | 353 |
| Consumables |  |  |  |  |  |  |  |  |  |
| Services | 4 | 2 | 250 | 3 | 1 | 0 |  |  |  |
| Others |  |  |  |  |  |  |  |  |  |
| T2DM+Myocardial infarction | | | | | | | | | |
| Medication | 5 | 3 | 570 | 3 | 4 | 2,691 | 4 | 4 | 699 |
| Procedure | 2 | 2 | 127 | 4 | 3 | 6,154 | 3 | 2 | 157 |
| Consultation | 5 | 4 | 197 | 3 | 5 | 485 | 4 | 4 | 135 |
| Consumables |  |  |  |  |  |  |  |  |  |
| Services |  |  |  | 2 | 2 | 7,845 |  |  |  |
| Others |  |  |  |  |  |  |  |  |  |
| T2DM+Other Cardiovascular Disease | | | | | | | | | |
| Medication | 4 | 6 | 1,599 | 4 | 4 | 1,340 | 3 | 4 | 811 |
| Procedure | 3 | 3 | 1,199 | 4 | 2 | 1,568 | 3 | 2 | 590 |
| Consultation | 5 | 3 | 150 | 5 | 3 | 268 | 3 | 5 | 130 |
| Consumables |  |  |  |  |  |  |  |  |  |
| Services |  |  |  |  |  |  |  |  |  |
| Others |  |  |  |  |  |  |  |  |  |
| T2DM+Periphery vascular disease | | | | | | | | | |
| Medication | 1 | 8 | 3,761 | 1 | 6 | 3,711 | 1 | 3 | 4,422 |
| Procedure | 1 | 9 | 3,037 | 1 | 5 | 1,017 | 1 | 5 | 2,307 |
| Consultation | 1 | 9 | 1,130 | 1 | 6 | 680 | 1 | 4 | 730 |
| Consumables |  |  |  |  |  |  |  |  |  |
| Services |  |  |  |  |  |  |  |  |  |
| Others |  |  |  |  |  |  |  |  |  |
| T2DM+Stroke or TIA | | | | | | | | | |
| Medication | 47 | 4 | 1,883 | 45 | 5 | 2,544 | 40 | 4 | 2,408 |
| Procedure | 39 | 2 | 658 | 36 | 3 | 1,166 | 29 | 2 | 873 |
| Consultation | 48 | 3 | 208 | 47 | 5 | 490 | 36 | 4 | 561 |
| Consumables |  |  |  | 1 | 1 | 8 |  |  |  |
| Services | 9 | 1 | 509 | 14 | 1 | 665 | 8 | 1 | 98 |
| Others | 1 | 1 | 110 |  |  |  |  |  |  |
| **T2DM With Multiple CVD** | | | | | | | | | |
| T2DM+Coronary Artery Disease+Angina | | | | | | | | | |
| Medication | 27 | 4 | 1,553 | 32 | 6 | 3,490 | 32 | 5 | 3,143 |
| Procedure | 19 | 3 | 1,731 | 26 | 4 | 15,912 | 25 | 3 | 9,904 |
| Consultation | 28 | 4 | 231 | 32 | 6 | 499 | 31 | 5 | 323 |
| Consumables |  |  |  |  |  |  | 1 | 1 | 301 |
| Services | 8 | 1 | 1,056 | 12 | 2 | 7,316 | 11 | 1 | 801 |
| Others | 1 | 1 | 1,107 | 1 | 1 | 523 |  |  |  |
| T2DM+Coronary Artery Disease+Atrial fibrillation | | | | | | | | | |
| Medication | 7 | 4 | 2,295 | 7 | 6 | 5,556 | 7 | 6 | 5,247 |
| Procedure | 4 | 2 | 2,107 | 6 | 4 | 5,125 | 6 | 3 | 3,974 |
| Consultation | 7 | 4 | 397 | 7 | 6 | 696 | 7 | 5 | 452 |
| Consumables |  |  |  |  |  |  |  |  |  |
| Services | 2 | 1 | 815 | 5 | 1 | 14,229 | 2 | 2 | 683 |
| Others |  |  |  |  |  |  |  |  |  |
| T2DM+Coronary Artery Disease+Chronic renal failure | | | | | | | | | |
| Medication | 3 | 5 | 6,497 | 4 | 11 | 13,066 | 5 | 9 | 6,947 |
| Procedure | 4 | 3 | 1,517 | 5 | 5 | 5,448 | 5 | 6 | 20,446 |
| Consultation | 4 | 4 | 420 | 5 | 8 | 1,017 | 5 | 8 | 872 |
| Consumables |  |  |  |  |  |  |  |  |  |
| Services |  |  |  | 3 | 3 | 2,815 | 3 | 1 | 17 |
| Others |  |  |  |  |  |  |  |  |  |
| T2DM+Heart Failure+Coronary Artery Disease | | | | | | | | | |
| Medication | 13 | 5 | 1,853 | 13 | 7 | 4,020 | 12 | 5 | 1,959 |
| Procedure | 11 | 4 | 1,778 | 13 | 4 | 7,761 | 9 | 3 | 1,719 |
| Consultation | 13 | 5 | 379 | 13 | 7 | 753 | 11 | 4 | 372 |
| Consumables |  |  |  | 2 | 1 | 8,293 | 1 | 1 | 313 |
| Services | 4 | 1 | 571 | 6 | 2 | 4,813 | 3 | 1 | 1,522 |
| Others |  |  |  |  |  |  |  |  |  |
| T2DM+Myocardial infarction+Coronary Artery Disease | | | | | | | | | |
| Medication | 17 | 4 | 1,382 | 17 | 8 | 4,359 | 18 | 5 | 2,242 |
| Procedure | 15 | 2 | 896 | 16 | 5 | 25,878 | 10 | 3 | 8,301 |
| Consultation | 17 | 5 | 241 | 17 | 8 | 619 | 17 | 4 | 281 |
| Consumables |  |  |  |  |  |  |  |  |  |
| Services | 4 | 1 | 66 | 11 | 3 | 4,964 | 3 | 2 | 2,843 |
| Others | 3 | 1 | 214 |  |  |  |  |  |  |
| T2DM+Stroke or TIA+Angina | | | | | | | | | |
| Medication | 2 | 1 | 77 | 2 | 2 | 582 |  |  |  |
| Procedure | 3 | 1 | 465 | 3 | 2 | 2,232 | 1 | 1 | 960 |
| Consultation | 3 | 1 | 33 | 3 | 2 | 183 |  |  |  |
| Consumables |  |  |  |  |  |  |  |  |  |
| Services |  |  |  | 1 | 1 | 226 |  |  |  |
| Others |  |  |  |  |  |  | 1 | 1 | 250 |
| T2DM+Stroke or TIA+Coronary Artery Disease | | | | | | | | | |
| Medication | 18 | 4 | 2,149 | 18 | 7 | 5,337 | 20 | 4 | 3,761 |
| Procedure | 15 | 3 | 2,099 | 16 | 4 | 5,917 | 17 | 2 | 4,174 |
| Consultation | 18 | 4 | 388 | 18 | 8 | 1,181 | 18 | 5 | 580 |
| Consumables |  |  |  | 2 | 2 | 4,042 |  |  |  |
| Services | 1 | 1 | 135 | 11 | 1 | 6,677 | 9 | 1 | 820 |
| Others |  |  |  |  |  |  |  |  |  |

Abbreviations: CVD=Cardiovascular disease, HCRU=Healthcare cost utilization, N=Number of patients, T2DM=Type 2 diabetes mellitus, TIA=Transient ischemic attack
