# Supplementary material for: Cost of cardiovascular diseases and renal complications in people with type 2 diabetes mellitus in the Kingdom of Saudi Arabia: A retrospective analysis of claims database
Source: PLoS One. 2022 Oct 20;17(10):e0273836. doi: 10.1371/journal.pone.0273836 (PMC9584438; doi:10.1371/journal.pone.0273836)
Supplement: S20 Table — (DOCX) [file pone.0273836.s020.docx]

### S20 Table: Comparison of pre-index and post-index disease-specific cause cost for various activities (Payer 2, Cohort 3)

| **Disease-specific Cause** | **Pre-Index 1 Yr** | | | **Post-Index 1 Yr** | | | **Post-Index 2 Yr** | | | **Post-Index 3 Yr** | | |
| --- | --- | --- | --- | --- | --- | --- | --- | --- | --- | --- | --- | --- |
| **Payer 2** |  |  |  | **Disease-specific Cause** |  |  | **Disease-specific Cause** |  |  | **Disease-specific Cause** |  |  |
| **Cohort 3** | **N** | **HCRU** | **Cost** | **N** | **HCRU** | **Cost** | **N** | **HCRU** | **Cost** | **N** | **HCRU** | **Cost** |
| **T2DM WITH ONE CVD** | | | | | | | | | | | | |
| **T2DM+Angina** | **12** | **8** | **4,134** | **9** | **11** | **5,965** | **9** | **11** | **3,595** | **11** | **7** | **2,710** |
| Medication | 3 | 3 | 2,197 | 3 | 4 | 3,139 | 3 | 3 | 1,330 | 3 | 2 | 1,585 |
| Procedure | 4 | 2 | 744 | 3 | 3 | 2,254 | 3 | 3 | 1,464 | 4 | 2 | 844 |
| Consultation | 4 | 3 | 349 | 3 | 5 | 573 | 2 | 4 | 322 | 4 | 3 | 282 |
| Consumables |  |  |  |  |  |  |  |  |  |  |  |  |
| Services | 1 | 1 | 844 |  |  |  | 1 | 2 | 479 |  |  |  |
| Others |  |  |  |  |  |  |  |  |  |  |  |  |
| **T2DM+Atrial fibrillation** | **1** | **3** | **589** | **1** | **2** | **556** | **3** | **3** | **1,504** | **1** | **1** | **1,422** |
| Medication | 1 | 3 | 589 | 1 | 2 | 556 | 1 | 1 | 208 | 1 | 1 | 1,422 |
| Procedure |  |  |  |  |  |  |  |  |  |  |  |  |
| Consultation |  |  |  |  |  |  | 1 | 1 | 50 |  |  |  |
| Consumables |  |  |  |  |  |  |  |  |  |  |  |  |
| Services |  |  |  |  |  |  | 1 | 1 | 1,246 |  |  |  |
| Others |  |  |  |  |  |  |  |  |  |  |  |  |
| **T2DM+Chronic renal failure** | **12** | **10** | **5,817** | **11** | **15** | **14,386** | **12** | **19** | **84,802** | **11** | **23** | **81,102** |
| Medication | 3 | 3 | 2,539 | 3 | 6 | 7,192 | 4 | 4 | 7,362 | 3 | 6 | 7,840 |
| Procedure | 4 | 2 | 1,973 | 3 | 4 | 5,175 | 3 | 7 | 66,267 | 2 | 11 | 65,940 |
| Consultation | 3 | 3 | 668 | 3 | 4 | 806 | 4 | 4 | 917 | 4 | 4 | 1,427 |
| Consumables |  |  |  |  |  |  |  |  |  |  |  |  |
| Services | 2 | 2 | 638 | 2 | 1 | 1,214 | 1 | 4 | 10,256 | 2 | 3 | 5,896 |
| Others |  |  |  |  |  |  |  |  |  |  |  |  |
| **T2DM+Coronary Artery Disease** | **64** | **12** | **3,127** | **61** | **17** | **8,504** | **55** | **16** | **9,008** | **49** | **12** | **5,260** |
| Medication | 21 | 4 | 1,731 | 21 | 6 | 3,130 | 16 | 6 | 3,657 | 17 | 4 | 2,372 |
| Procedure | 17 | 2 | 933 | 16 | 4 | 1,771 | 15 | 3 | 4,486 | 12 | 3 | 1,923 |
| Consultation | 20 | 4 | 330 | 21 | 5 | 432 | 17 | 6 | 448 | 16 | 4 | 326 |
| Consumables |  |  |  |  |  |  |  |  |  |  |  |  |
| Services | 3 | 1 | 63 | 3 | 2 | 3,171 | 7 | 1 | 417 | 4 | 1 | 638 |
| Others | 3 | 1 | 69 |  |  |  |  |  |  |  |  |  |
| **T2DM+Other Cardiovascular Disease** | **3** | **6** | **1,335** | **3** | **11** | **3,363** | **3** | **4** | **1,276** | **3** | **7** | **4,565** |
| Medication | 1 | 2 | 765 | 1 | 5 | 2,253 | 1 | 1 | 1,146 | 1 | 3 | 4,165 |
| Procedure | 1 | 2 | 370 | 1 | 1 | 540 | 1 | 2 | 40 | 1 | 2 | 160 |
| Consultation | 1 | 2 | 200 | 1 | 5 | 570 | 1 | 1 | 90 | 1 | 2 | 240 |
| Consumables |  |  |  |  |  |  |  |  |  |  |  |  |
| Services |  |  |  |  |  |  |  |  |  |  |  |  |
| Others |  |  |  |  |  |  |  |  |  |  |  |  |
| **T2DM+Stroke or TIA** | **20** | **11** | **4,075** | **18** | **13** | **3,543** | **19** | **15** | **5,389** | **15** | **17** | **7,357** |
| Medication | 6 | 5 | 2,798 | 6 | 5 | 2,351 | 6 | 7 | 3,693 | 5 | 6 | 4,672 |
| Procedure | 6 | 2 | 473 | 4 | 2 | 906 | 5 | 2 | 1,152 | 3 | 3 | 1,787 |
| Consultation | 7 | 4 | 152 | 7 | 4 | 278 | 6 | 6 | 327 | 4 | 7 | 294 |
| Consumables |  |  |  | 1 | 1 | 8 |  |  |  | 1 | 1 | 466 |
| Services | 1 | 1 | 653 |  |  |  | 2 | 1 | 217 | 2 | 1 | 139 |
| Others |  |  |  |  |  |  |  |  |  |  |  |  |
| **T2DM WITH MULTIPLE CVD** | | | | | | | | | | | | |
| **T2DM+Coronary Arterial Revascularization+Coronary Artery Disease+Atrial fibrillation+Angina** | **3** | **4** | **1,073** | **4** | **36** | **48,691** | **3** | **16** | **7,071** | **3** | **8** | **9,016** |
| Medication | 1 | 1 | 503 | 1 | 11 | 5,401 | 1 | 5 | 4,680 | 1 | 5 | 3,645 |
| Procedure | 1 | 1 | 450 | 1 | 10 | 33,229 | 1 | 6 | 1,945 | 1 | 1 | 5,150 |
| Consultation | 1 | 2 | 120 | 1 | 10 | 1,160 | 1 | 5 | 445 | 1 | 2 | 220 |
| Consumables |  |  |  |  |  |  |  |  |  |  |  |  |
| Services |  |  |  | 1 | 5 | 8,901 |  |  |  |  |  |  |
| Others |  |  |  |  |  |  |  |  |  |  |  |  |
| **T2DM+Coronary Artery Disease+Angina** | **10** | **14** | **2,432** | **15** | **14** | **32,485** | **14** | **16** | **3,271** | **12** | **14** | **5,329** |
| Medication | 3 | 4 | 528 | 4 | 5 | 2,490 | 4 | 6 | 1,740 | 3 | 3 | 631 |
| Procedure | 2 | 5 | 1,677 | 3 | 3 | 29,020 | 4 | 3 | 915 | 3 | 3 | 4,046 |
| Consultation | 4 | 4 | 208 | 4 | 5 | 396 | 3 | 7 | 545 | 3 | 4 | 142 |
| Consumables |  |  |  |  |  |  |  |  |  | 1 | 2 | 364 |
| Services | 1 | 2 | 20 | 4 | 2 | 578 | 3 | 1 | 71 | 2 | 3 | 145 |
| Others |  |  |  |  |  |  |  |  |  |  |  |  |
| **T2DM+Coronary Artery Disease+Chronic renal failure** | **6** | **12** | **8,392** | **7** | **45** | **35,503** | **7** | **36** | **44,224** | **8** | **20** | **20,669** |
| Medication | 2 | 5 | 6,275 | 2 | 17 | 19,546 | 2 | 13 | 13,646 | 2 | 8 | 13,844 |
| Procedure | 2 | 3 | 1,767 | 2 | 9 | 8,360 | 2 | 9 | 29,191 | 2 | 4 | 5,942 |
| Consultation | 2 | 5 | 350 | 2 | 16 | 1,592 | 2 | 14 | 1,387 | 2 | 7 | 657 |
| Consumables |  |  |  |  |  |  |  |  |  | 1 | 1 | 168 |
| Services |  |  |  | 1 | 4 | 6,007 | 1 | 1 | 0 | 1 | 1 | 58 |
| Others |  |  |  |  |  |  |  |  |  |  |  |  |
| **T2DM+Heart Failure+Coronary Artery Disease** | **2** | **4** | **1,356** | **4** | **22** | **3,237** | **5** | **8** | **9,798** | **4** | **33** | **17,835** |
| Medication | 1 | 2 | 1,136 | 1 | 10 | 2,286 | 1 | 3 | 2,507 | 1 | 10 | 5,659 |
| Procedure |  |  |  | 1 | 3 | 116 | 1 | 1 | 2,473 | 1 | 6 | 9,220 |
| Consultation | 1 | 2 | 220 | 1 | 6 | 550 | 1 | 2 | 696 | 1 | 10 | 1,996 |
| Consumables |  |  |  |  |  |  | 1 | 1 | 313 |  |  |  |
| Services |  |  |  | 1 | 3 | 286 | 1 | 1 | 3,808 | 1 | 7 | 959 |
| Others |  |  |  |  |  |  |  |  |  |  |  |  |
| **T2DM+Myocardial infarction+Coronary Artery Disease** | **3** | **3** | **237** | **3** | **20** | **28,322** | **3** | **26** | **1,452** | **2** | **4** | **256** |
| Medication | 1 | 1 | 122 | 1 | 7 | 1,504 | 1 | 15 | 1,017 | 1 | 2 | 216 |
| Procedure | 1 | 1 | 85 | 1 | 6 | 26,534 | 1 | 4 | 295 |  |  |  |
| Consultation | 1 | 1 | 30 | 1 | 7 | 284 | 1 | 7 | 140 | 1 | 2 | 40 |
| Consumables |  |  |  |  |  |  |  |  |  |  |  |  |
| Services |  |  |  |  |  |  |  |  |  |  |  |  |
| Others |  |  |  |  |  |  |  |  |  |  |  |  |
| **T2DM+Other Cardiovascular Disease+Coronary Artery Disease** | **4** | **15** | **4,187** | **4** | **21** | **7,529** | **3** | **31** | **6,867** | **3** | **12** | **3,881** |
| Medication | 1 | 5 | 865 | 1 | 6 | 2,453 | 1 | 12 | 3,050 | 1 | 5 | 2,231 |
| Procedure | 1 | 4 | 1,367 | 1 | 4 | 3,605 | 1 | 10 | 3,392 | 1 | 3 | 1,460 |
| Consultation | 1 | 4 | 225 | 1 | 8 | 832 | 1 | 9 | 425 | 1 | 4 | 190 |
| Consumables |  |  |  |  |  |  |  |  |  |  |  |  |
| Services | 1 | 2 | 1,730 | 1 | 3 | 639 |  |  |  |  |  |  |
| Others |  |  |  |  |  |  |  |  |  |  |  |  |
| **T2DM+Stroke or TIA+Coronary Artery Disease** | **6** | **10** | **3,304** | **7** | **14** | **6,436** | **6** | **10** | **4,683** | **6** | **8** | **3,684** |
| Medication | 2 | 4 | 1,590 | 2 | 5 | 2,234 | 2 | 4 | 2,987 | 2 | 3 | 2,516 |
| Procedure | 2 | 3 | 1,472 | 2 | 3 | 3,014 | 2 | 2 | 1,310 | 2 | 2 | 718 |
| Consultation | 2 | 4 | 242 | 2 | 6 | 1,021 | 2 | 4 | 387 | 2 | 3 | 450 |
| Consumables |  |  |  |  |  |  |  |  |  |  |  |  |
| Services |  |  |  | 1 | 1 | 167 |  |  |  |  |  |  |
| Others |  |  |  |  |  |  |  |  |  |  |  |  |

Abbreviations: CVD=Cardiovascular disease, HCRU=Healthcare cost utilization, N=Number of patients, T2DM=Type 2 diabetes mellitus, TIA=Transient ischemic attack
