# Supplementary material for: Cost of cardiovascular diseases and renal complications in people with type 2 diabetes mellitus in the Kingdom of Saudi Arabia: A retrospective analysis of claims database
Source: PLoS One. 2022 Oct 20;17(10):e0273836. doi: 10.1371/journal.pone.0273836 (PMC9584438; doi:10.1371/journal.pone.0273836)
Supplement: S21 Table — (DOCX) [file pone.0273836.s021.docx]

### S21 Table: Comparison of pre-index and post-index disease-specific cause cost for various activities (Payer 3, Cohort 1)

|  | **Pre-Index 1 Yr** | | | **Post-Index 1 Yr** | | |
| --- | --- | --- | --- | --- | --- | --- |
| **Payer 3** | **Disease-specific Cause** | | | **Disease-specific Cause** | | |
| **Cohort 1** | **N** | **HCRU** | **Cost** | **N** | **HCRU** | **Cost** |
| **T2DM With One CVD** | | | | | | |
| Coronary Arterial Revascularization+T2DM | | | | | | |
| Medication | 32 | 4 | 7,753 | 33 | 6 | 11,142 |
| Procedure | 26 | 2 | 7,271 | 30 | 3 | 7,032 |
| Consultation | 33 | 4 | 841 | 32 | 7 | 1,462 |
| Consumables | 6 | 3 | 575 | 6 | 3 | 882 |
| Services | 1 | 1 | 3,530 | 2 | 2 | 2,130 |
| Others | 2 | 1 | 80 | 2 | 1 | 80 |
| T2DM+Angina | | | | | | |
| Medication | 396 | 4 | 2,769 | 376 | 5 | 3,790 |
| Procedure | 358 | 3 | 2,434 | 354 | 3 | 3,176 |
| Consultation | 420 | 4 | 575 | 398 | 5 | 725 |
| Consumables | 31 | 2 | 336 | 57 | 2 | 326 |
| Services | 41 | 1 | 2,032 | 55 | 1 | 1,842 |
| Others | 18 | 1 | 1,377 | 24 | 1 | 59 |
| T2DM+Atrial fibrillation | | | | | | |
| Medication | 50 | 3 | 3,948 | 55 | 5 | 5,369 |
| Procedure | 45 | 3 | 3,464 | 47 | 4 | 4,979 |
| Consultation | 54 | 3 | 722 | 53 | 6 | 1,358 |
| Consumables | 7 | 2 | 415 | 12 | 2 | 342 |
| Services | 6 | 1 | 2,251 | 10 | 2 | 2,474 |
| Others | 2 | 1 | 0 | 2 | 2 | 55 |
| T2DM+cardiac ischemia | | | | | | |
| Medication | 1 | 4 | 2,102 | 1 | 3 | 2,782 |
| Procedure | 1 | 1 | 520 | 1 | 1 | 2,165 |
| Consultation | 1 | 4 | 370 | 1 | 3 | 893 |
| Consumables |  |  |  |  |  |  |
| Services |  |  |  |  |  |  |
| Others |  |  |  |  |  |  |
| T2DM+Chronic renal failure | | | | | | |
| Medication | 200 | 4 | 4,805 | 202 | 6 | 7,024 |
| Procedure | 182 | 3 | 3,744 | 195 | 5 | 7,905 |
| Consultation | 212 | 4 | 879 | 207 | 6 | 1,388 |
| Consumables | 32 | 2 | 869 | 50 | 3 | 954 |
| Services | 25 | 1 | 5,141 | 52 | 3 | 13,203 |
| Others | 13 | 1 | 120 | 15 | 2 | 1,545 |
| T2DM+Coronary Artery Disease | | | | | | |
| Medication | 937 | 4 | 3,363 | 915 | 5 | 4,799 |
| Procedure | 711 | 3 | 2,369 | 745 | 3 | 4,267 |
| Consultation | 948 | 4 | 569 | 920 | 5 | 775 |
| Consumables | 106 | 2 | 538 | 118 | 2 | 1,098 |
| Services | 108 | 1 | 1,668 | 171 | 1 | 2,697 |
| Others | 51 | 1 | 69 | 47 | 1 | 77 |
| T2DM+Dysrhythmia1 | | | | | | |
| Medication | 49 | 4 | 1,983 | 48 | 6 | 2,599 |
| Procedure | 41 | 3 | 2,503 | 40 | 4 | 6,330 |
| Consultation | 53 | 4 | 622 | 48 | 6 | 827 |
| Consumables | 2 | 3 | 310 | 5 | 1 | 100 |
| Services | 9 | 1 | 1,817 | 9 | 2 | 33,965 |
| Others | 2 | 2 | 56 | 1 | 1 | 80 |
| T2DM+Heart Failure | 466 | 15 | 19,416 | 461 | 19 | 26,320 |
| Medication | 137 | 4 | 4,734 | 134 | 5 | 6,645 |
| Procedure | 123 | 3 | 3,610 | 121 | 3 | 5,755 |
| Consultation | 146 | 4 | 799 | 132 | 5 | 1,620 |
| Consumables | 27 | 2 | 1,709 | 33 | 3 | 1,927 |
| Services | 19 | 1 | 8,506 | 30 | 2 | 10,320 |
| Others | 14 | 1 | 58 | 11 | 1 | 53 |
| T2DM+Myocardial infarction | | | | | | |
| Medication | 44 | 4 | 2,389 | 42 | 4 | 2,571 |
| Procedure | 39 | 3 | 1,454 | 38 | 2 | 5,289 |
| Consultation | 46 | 4 | 443 | 43 | 4 | 572 |
| Consumables | 1 | 3 | 300 | 4 | 2 | 468 |
| Services | 3 | 1 | 60 | 9 | 1 | 5,238 |
| Others | 4 | 2 | 106 | 3 | 1 | 5,360 |
| T2DM+Other Cardiovascular Disease | | | | | | |
| Medication | 22 | 5 | 3,705 | 22 | 6 | 3,734 |
| Procedure | 21 | 4 | 3,476 | 22 | 3 | 3,449 |
| Consultation | 24 | 5 | 741 | 21 | 6 | 884 |
| Consumables | 4 | 3 | 261 | 2 | 4 | 436 |
| Services | 1 | 1 | 40 | 3 | 1 | 505 |
| Others | 1 | 1 | 30 |  |  |  |
| T2DM+Periphery vascular disease | | | | | | |
| Medication | 13 | 3 | 3,176 | 13 | 4 | 4,551 |
| Procedure | 8 | 2 | 1,311 | 11 | 3 | 3,481 |
| Consultation | 13 | 4 | 466 | 13 | 5 | 687 |
| Consumables | 3 | 2 | 498 | 3 | 3 | 402 |
| Services | 5 | 6 | 867 | 3 | 22 | 13,654 |
| Others |  |  |  | 1 | 1 | 0 |
| T2DM+Stroke or TIA | | | | | | |
| Medication | 432 | 4 | 3,552 | 453 | 5 | 4,727 |
| Procedure | 397 | 3 | 2,577 | 398 | 4 | 4,358 |
| Consultation | 466 | 4 | 735 | 459 | 6 | 1,345 |
| Consumables | 51 | 3 | 472 | 83 | 3 | 508 |
| Services | 44 | 2 | 3,402 | 84 | 2 | 6,629 |
| Others | 21 | 1 | 57 | 18 | 1 | 102 |
| **T2DM With Multiple CVD**700 | | | | | | |
| Coronary Arterial Revascularization+T2DM+Coronary Artery Disease6 | | | | | | |
| Medication | 49 | 4 | 4,935 | 53 | 7 | 6,583 |
| Procedure | 41 | 3 | 6,074 | 45 | 4 | 9,983 |
| Consultation | 53 | 4 | 643 | 53 | 7 | 1,067 |
| Consumables | 3 | 2 | 5,434 | 3 | 2 | 2,737 |
| Services | 7 | 1 | 876 | 15 | 2 | 5,021 |
| Others | 6 | 1 | 79 | 5 | 1 | 3,715 |
| Coronary Arterial Revascularization+T2DM+Coronary Artery Disease+Angina | | | | | | |
| Medication | 17 | 5 | 4,835 | 19 | 8 | 10,813 |
| Procedure | 15 | 4 | 5,320 | 17 | 6 | 28,975 |
| Consultation | 17 | 5 | 907 | 19 | 8 | 1,633 |
| Consumables | 1 | 1 | 318 | 8 | 2 | 3,039 |
| Services | 3 | 1 | 1,102 | 6 | 2 | 4,647 |
| Others | 2 | 1 | 25 | 4 | 1 | 2,713 |
| T2DM+Coronary Artery Disease+Angina | | | | | | |
| Medication | 175 | 5 | 3,952 | 183 | 7 | 6,223 |
| Procedure | 147 | 3 | 2,585 | 166 | 4 | 15,989 |
| Consultation | 177 | 4 | 632 | 184 | 7 | 1,146 |
| Consumables | 14 | 2 | 361 | 27 | 2 | 2,417 |
| Services | 27 | 1 | 1,796 | 86 | 2 | 4,531 |
| Others | 5 | 1 | 67 | 15 | 1 | 1,217 |
| T2DM+Coronary Artery Disease+Atrial fibrillation | | | | | | |
| Medication | 15 | 3 | 3,330 | 17 | 5 | 4,407 |
| Procedure | 10 | 3 | 2,590 | 14 | 4 | 3,121 |
| Consultation | 16 | 3 | 480 | 17 | 6 | 886 |
| Consumables | 2 | 4 | 313 | 1 | 1 | 84 |
| Services | 1 | 1 | 300 | 9 | 2 | 3,664 |
| Others | 1 | 1 | 0 | 1 | 1 | 0 |
| T2DM+Coronary Artery Disease+Chronic renal failure | | | | | | |
| Medication | 33 | 5 | 4,350 | 34 | 7 | 7,419 |
| Procedure | 28 | 3 | 5,067 | 29 | 4 | 6,415 |
| Consultation | 31 | 5 | 955 | 34 | 6 | 1,576 |
| Consumables | 2 | 4 | 10,973 | 6 | 2 | 337 |
| Services | 9 | 2 | 11,683 | 14 | 2 | 5,635 |
| Others | 1 | 1 | 100 | 4 | 1 | 233 |
| T2DM+Heart Failure+Coronary Artery Disease | | | | | | |
| Medication | 61 | 4 | 3,305 | 67 | 6 | 5,650 |
| Procedure | 57 | 3 | 1,632 | 60 | 4 | 14,240 |
| Consultation | 66 | 4 | 713 | 65 | 6 | 1,334 |
| Consumables | 9 | 2 | 149 | 14 | 2 | 8,702 |
| Services | 6 | 2 | 472 | 18 | 2 | 13,624 |
| Others | 2 | 1 | 0 | 8 | 2 | 246 |
| Abbreviations: CVD=Cardiovascular disease, HCRU=Healthcare cost utilization, N=Number of patients, T2DM=Type 2 diabetes mellitus, TIA=Transient ischemic attack | | | | | | |
