# Supplementary material for: Cost of cardiovascular diseases and renal complications in people with type 2 diabetes mellitus in the Kingdom of Saudi Arabia: A retrospective analysis of claims database
Source: PLoS One. 2022 Oct 20;17(10):e0273836. doi: 10.1371/journal.pone.0273836 (PMC9584438; doi:10.1371/journal.pone.0273836)
Supplement: S22 Table — (DOCX) [file pone.0273836.s022.docx]

### S22 Table: Comparison of pre-index and post-index disease-specific cause cost for various activities (Payer 3, Cohort 2)

|  | **Pre-Index 1 Yr** | | | **Post-Index 1 Yr** | | | **Post-Index 2 Yr** | | |
| --- | --- | --- | --- | --- | --- | --- | --- | --- | --- |
| **Payer 3** | **Disease-specific Cause** | | | **Disease-specific Cause** | | | **Disease-specific Cause** | | |
| **Cohort 2** | **N** | **HCRU** | **Cost** | **N** | **HCRU** | **Cost** | **N** | **HCRU** | **Cost** |
| **T2DM With One CVD8**  **108,506** | | | | | | | | | |
| Coronary Arterial Revascularization+T2DM | | | | | | | | | |
| Medication | 10 | 4 | 5,033 | 10 | 9 | 14,538 | 11 | 6 | 6,646 |
| Procedure | 8 | 2 | 13,735 | 10 | 3 | 12,599 | 9 | 2 | 2,023 |
| Consultation | 11 | 3 | 418 | 10 | 9 | 1,617 | 11 | 6 | 892 |
| Consumables |  |  |  |  |  |  | 2 | 2 | 253 |
| Services | 1 | 1 | 3,530 | 2 | 2 | 2,130 | 1 | 1 | 200 |
| Others | 1 | 1 | 160 | 1 | 1 | 160 |  |  |  |
| T2DM+Angina | | | | | | | | | |
| Medication | 138 | 4 | 3,287 | 134 | 5 | 5,073 | 134 | 4 | 3,375 |
| Procedure | 130 | 3 | 2,814 | 130 | 4 | 4,280 | 126 | 3 | 2,011 |
| Consultation | 147 | 4 | 637 | 138 | 5 | 961 | 134 | 4 | 557 |
| Consumables | 13 | 3 | 367 | 29 | 3 | 376 | 19 | 2 | 244 |
| Services | 9 | 1 | 1,744 | 19 | 1 | 1,816 | 4 | 1 | 71 |
| Others | 5 | 1 | 168 | 10 | 1 | 39 | 6 | 1 | 40 |
| T2DM+Atrial fibrillation | | | | | | | | | |
| Medication | 21 | 4 | 4,847 | 22 | 7 | 7,326 | 22 | 5 | 5,495 |
| Procedure | 17 | 3 | 4,852 | 20 | 5 | 6,123 | 20 | 3 | 2,427 |
| Consultation | 22 | 4 | 1,002 | 22 | 7 | 1,946 | 21 | 5 | 998 |
| Consumables | 7 | 2 | 415 | 8 | 3 | 403 | 2 | 2 | 135 |
| Services | 2 | 1 | 4,807 | 4 | 2 | 4,355 | 3 | 1 | 556 |
| Others | 1 | 1 | 0 | 1 | 1 | 0 | 1 | 1 | 0 |
| T2DM+cardiac ischemia | | | | | | | | | |
| Medication | 1 | 4 | 2,102 | 1 | 3 | 2,782 | 1 | 1 | 621 |
| Procedure | 1 | 1 | 520 | 1 | 1 | 2,165 | 1 | 2 | 1,150 |
| Consultation | 1 | 4 | 370 | 1 | 3 | 893 | 1 | 3 | 210 |
| Consumables |  |  |  |  |  |  |  |  |  |
| Services |  |  |  |  |  |  |  |  |  |
| Others |  |  |  |  |  |  |  |  |  |
| T2DM+Chronic renal failure**1** | | | | | | | | | |
| Medication | 64 | 4 | 5,456 | 64 | 6 | 8,192 | 67 | 6 | 5,258 |
| Procedure | 63 | 3 | 3,659 | 65 | 5 | 8,948 | 66 | 5 | 7,171 |
| Consultation | 67 | 4 | 962 | 66 | 6 | 1,648 | 70 | 5 | 1,104 |
| Consumables | 12 | 3 | 1,247 | 15 | 3 | 1,081 | 17 | 2 | 370 |
| Services | 7 | 1 | 1,388 | 16 | 2 | 12,824 | 12 | 3 | 5,038 |
| Others |  |  |  | 3 | 1 | 43 | 6 | 1 | 19 |
| T2DM+Coronary Artery Disease | | | | | | | | | |
| Medication | 347 | 4 | 3,656 | 341 | 6 | 5,651 | 333 | 5 | 3,829 |
| Procedure | 276 | 3 | 2,644 | 290 | 4 | 5,159 | 270 | 3 | 2,416 |
| Consultation | 347 | 4 | 642 | 343 | 6 | 927 | 329 | 5 | 563 |
| Consumables | 45 | 3 | 850 | 56 | 3 | 447 | 47 | 2 | 291 |
| Services | 34 | 1 | 1,088 | 64 | 1 | 1,387 | 44 | 2 | 1,237 |
| Others | 26 | 1 | 43 | 25 | 1 | 45 | 15 | 1 | 82 |
| T2DM+Dysrhythmia | | | | | | | | | |
| Medication | 18 | 4 | 2,100 | 21 | 7 | 3,525 | 20 | 6 | 3,237 |
| Procedure | 14 | 2 | 1,180 | 18 | 4 | 5,110 | 18 | 3 | 1,957 |
| Consultation | 20 | 4 | 525 | 21 | 6 | 840 | 20 | 5 | 560 |
| Consumables |  |  |  | 1 | 1 | 21 | 3 | 3 | 128 |
| Services | 3 | 1 | 304 | 4 | 2 | 72,716 | 1 | 1 | 1,420 |
| Others | 2 | 2 | 56 |  |  |  | 2 | 1 | 88 |
| T2DM+Heart Failure | | | | | | | | | |
| Medication | 51 | 4 | 5,430 | 50 | 6 | 9,153 | 50 | 5 | 5,913 |
| Procedure | 52 | 3 | 4,200 | 50 | 4 | 8,279 | 40 | 3 | 3,167 |
| Consultation | 53 | 4 | 1,108 | 50 | 7 | 2,365 | 50 | 4 | 935 |
| Consumables | 11 | 3 | 991 | 16 | 3 | 2,176 | 19 | 2 | 439 |
| Services | 4 | 1 | 13,347 | 13 | 2 | 12,616 | 4 | 1 | 3,345 |
| Others | 4 | 2 | 133 | 4 | 2 | 30 | 1 | 1 | 0 |
| T2DM+Myocardial infarction | | | | | | | | | |
| Medication | 10 | 4 | 2,247 | 9 | 4 | 2,728 | 10 | 4 | 3,358 |
| Procedure | 8 | 2 | 1,726 | 9 | 2 | 16,661 | 8 | 4 | 2,481 |
| Consultation | 9 | 4 | 544 | 10 | 3 | 886 | 10 | 4 | 464 |
| Consumables | 1 | 3 | 300 | 1 | 2 | 1,559 | 5 | 1 | 121 |
| Services |  |  |  | 3 | 1 | 14,031 |  |  |  |
| Others |  |  |  | 1 | 2 | 0 | 1 | 1 | 67 |
| T2DM+Other Cardiovascular Disease | | | | | | | | | |
| Medication | 13 | 6 | 5,015 | 15 | 6 | 4,633 | 14 | 4 | 3,228 |
| Procedure | 14 | 4 | 3,933 | 15 | 3 | 4,089 | 14 | 3 | 2,074 |
| Consultation | 15 | 5 | 953 | 14 | 7 | 1,103 | 15 | 4 | 549 |
| Consumables | 4 | 3 | 261 | 2 | 4 | 436 | 2 | 3 | 361 |
| Services | 1 | 1 | 40 | 3 | 1 | 505 | 3 | 1 | 130 |
| Others |  |  |  |  |  |  |  |  |  |
| T2DM+Periphery vascular disease | | | | | | | | | |
| Medication | 3 | 2 | 6,685 | 3 | 5 | 10,295 | 3 | 2 | 4,690 |
| Procedure | 2 | 3 | 2,581 | 3 | 5 | 7,390 | 3 | 2 | 2,821 |
| Consultation | 3 | 3 | 630 | 3 | 5 | 1,301 | 3 | 2 | 439 |
| Consumables | 1 | 4 | 1,200 | 1 | 4 | 1,086 | 1 | 2 | 504 |
| Services | 1 | 24 | 922 | 1 | 64 | 40,491 |  |  |  |
| Others |  |  |  |  |  |  |  |  |  |
| T2DM+Stroke or TIA | | | | | | | | | |
| Medication | 148 | 5 | 4,227 | 155 | 6 | 6,020 | 145 | 5 | 3,966 |
| Procedure | 142 | 3 | 3,064 | 140 | 4 | 5,116 | 139 | 3 | 2,877 |
| Consultation | 158 | 5 | 876 | 155 | 6 | 1,607 | 150 | 5 | 839 |
| Consumables | 24 | 3 | 339 | 28 | 3 | 522 | 33 | 3 | 368 |
| Services | 17 | 2 | 4,950 | 21 | 2 | 2,409 | 21 | 1 | 2,681 |
| Others | 9 | 1 | 34 | 7 | 1 | 20 | 5 | 1 | 20 |
| **T2DM With Multiple CVD** | | | | | | | | | |
| Coronary Arterial Revascularization+T2DM+Coronary Artery Disease | | | | | | | | | |
| Medication | 21 | 5 | 6,052 | 23 | 7 | 7,628 | 22 | 7 | 6,326 |
| Procedure | 20 | 4 | 11,133 | 23 | 4 | 9,305 | 21 | 4 | 11,968 |
| Consultation | 23 | 5 | 902 | 23 | 7 | 1,442 | 22 | 7 | 1,337 |
| Consumables | 3 | 2 | 5,434 | 2 | 2 | 3,917 | 1 | 2 | 318 |
| Services | 4 | 1 | 1,424 | 7 | 2 | 2,855 | 6 | 2 | 1,250 |
| Others | 5 | 1 | 63 | 2 | 1 | 11 | 2 | 1 | 0 |
| Coronary Arterial Revascularization+T2DM+Coronary Artery Disease+Angina | | | | | | | | | |
| Medication | 8 | 4 | 6,437 | 8 | 7 | 10,310 | 8 | 8 | 8,824 |
| Procedure | 8 | 3 | 4,716 | 6 | 5 | 17,844 | 5 | 4 | 12,887 |
| Consultation | 8 | 5 | 998 | 8 | 7 | 1,166 | 8 | 6 | 1,307 |
| Consumables |  |  |  | 2 | 1 | 101 | 1 | 4 | 588 |
| Services | 1 | 1 | 315 | 1 | 2 | 1,500 | 1 | 3 | 9,656 |
| Others |  |  |  | 1 | 2 | 10,800 | 1 | 1 | 0 |
| T2DM+Coronary Artery Disease+Angina | | | | | | | | | |
| Medication | 72 | 5 | 4,404 | 75 | 8 | 7,611 | 72 | 6 | 4,702 |
| Procedure | 58 | 4 | 2,773 | 67 | 5 | 15,304 | 64 | 4 | 7,410 |
| Consultation | 71 | 5 | 648 | 74 | 8 | 1,347 | 72 | 6 | 792 |
| Consumables | 7 | 2 | 365 | 12 | 3 | 4,879 | 12 | 3 | 981 |
| Services | 12 | 2 | 1,214 | 33 | 2 | 5,372 | 17 | 1 | 2,353 |
| Others | 3 | 1 | 12 | 10 | 1 | 1,811 | 5 | 2 | 216 |
| T2DM+Coronary Artery Disease+Atrial fibrillation | | | | | | | | | |
| Medication | 6 | 4 | 2,818 | 7 | 4 | 3,252 | 8 | 4 | 3,380 |
| Procedure | 4 | 4 | 839 | 5 | 5 | 2,603 | 6 | 5 | 3,410 |
| Consultation | 7 | 3 | 339 | 7 | 6 | 705 | 7 | 6 | 858 |
| Consumables | 1 | 6 | 475 |  |  |  | 1 | 1 | 3 |
| Services |  |  |  | 4 | 1 | 1,309 |  |  |  |
| Others | 1 | 1 | 0 | 1 | 1 | 0 | 1 | 1 | 100 |
| T2DM+Coronary Artery Disease+Chronic renal failure | | | | | | | | | |
| Medication | 13 | 4 | 4,326 | 14 | 7 | 9,129 | 13 | 5 | 7,474 |
| Procedure | 12 | 3 | 4,145 | 12 | 5 | 6,635 | 12 | 4 | 7,362 |
| Consultation | 13 | 4 | 790 | 14 | 6 | 2,298 | 13 | 5 | 1,120 |
| Consumables | 1 | 1 | 225 | 2 | 2 | 414 | 5 | 1 | 136 |
| Services | 3 | 1 | 3,395 | 5 | 2 | 9,217 | 7 | 2 | 7,447 |
| Others |  |  |  | 2 | 2 | 147 | 2 | 1 | 597 |
| T2DM+Heart Failure+Angina | | | | | | | | | |
| Medication | 6 | 4 | 2,038 | 7 | 8 | 4,134 | 7 | 6 | 4,746 |
| Procedure | 5 | 2 | 2,946 | 6 | 4 | 6,119 | 7 | 6 | 4,146 |
| Consultation | 7 | 3 | 472 | 8 | 6 | 1,070 | 8 | 7 | 1,183 |
| Consumables | 2 | 2 | 208 | 1 | 4 | 553 | 4 | 4 | 457 |
| Services | 1 | 1 | 800 | 3 | 1 | 3,338 | 2 | 1 | 769 |
| Others |  |  |  |  |  |  |  |  |  |

Abbreviations: CVD=Cardiovascular disease, HCRU=Healthcare cost utilization, N=Number of patients, T2DM=Type 2 diabetes mellitus, TIA=Transient ischemic attack
