# Supplementary material for: Cost of cardiovascular diseases and renal complications in people with type 2 diabetes mellitus in the Kingdom of Saudi Arabia: A retrospective analysis of claims database
Source: PLoS One. 2022 Oct 20;17(10):e0273836. doi: 10.1371/journal.pone.0273836 (PMC9584438; doi:10.1371/journal.pone.0273836)
Supplement: S23 Table — (DOCX) [file pone.0273836.s023.docx]

### S23 Table: Comparison of pre-index and post-index disease-specific cause cost for various activities (Payer 3, Cohort 3)

| **Disease-specific Cause** | **Pre-Index 1 Yr** | | | **Post-Index 1 Yr** | | | **Post-Index 2 Yr** | | | **Post-Index 3 Yr** | | |
| --- | --- | --- | --- | --- | --- | --- | --- | --- | --- | --- | --- | --- |
| **Payer 3** |  |  |  |  |  |  |  |  |  |  |  |  |
| **Cohort 3** | **N** | **HCRU** | **Cost** | **N** | **HCRU** | **Cost** | **N** | **HCRU** | **Cost** | **N** | **HCRU** | **Cost** |
| **T2DM WITH ONE CVD** | | | | | | | | | | | | |
| **Coronary Arterial Revascularization+T2DM** | **15** | **9** | **37,538** | **16** | **25** | **46,545** | **16** | **15** | **12,005** | **16** | **16** | **10,748** |
| Medication | 5 | 3 | 7,781 | 5 | 10 | 19,440 | 5 | 5 | 8,478 | 5 | 5 | 6,635 |
| Procedure | 4 | 2 | 25,724 | 5 | 4 | 21,899 | 5 | 2 | 2,175 | 5 | 2 | 1,801 |
| Consultation | 5 | 3 | 503 | 5 | 10 | 1,675 | 5 | 7 | 1,017 | 4 | 6 | 1,204 |
| Consumables |  |  |  |  |  |  | 1 | 1 | 336 | 1 | 2 | 336 |
| Services | 1 | 1 | 3,530 | 1 | 1 | 3,530 |  |  |  | 1 | 1 | 772 |
| Others |  |  |  |  |  |  |  |  |  |  |  |  |
| **T2DM+Angina** | **112** | **16** | **5,671** | **114** | **19** | **13,583** | **110** | **20** | **9,411** | **112** | **15** | **8,178** |
| Medication | 36 | 4 | 2,576 | 33 | 5 | 7,185 | 34 | 5 | 5,056 | 33 | 4 | 3,236 |
| Procedure | 33 | 3 | 1,865 | 33 | 4 | 4,653 | 31 | 4 | 3,134 | 33 | 3 | 2,428 |
| Consultation | 37 | 4 | 448 | 34 | 6 | 1,155 | 33 | 6 | 769 | 33 | 4 | 589 |
| Consumables | 4 | 4 | 697 | 8 | 2 | 440 | 7 | 2 | 319 | 8 | 2 | 200 |
| Services |  |  |  | 3 | 1 | 95 | 2 | 2 | 72 | 3 | 1 | 1,689 |
| Others | 2 | 2 | 85 | 3 | 1 | 55 | 3 | 1 | 60 | 2 | 1 | 35 |
| **T2DM+Atrial fibrillation** | **10** | **20** | **16,224** | **12** | **28** | **22,536** | **10** | **20** | **10,894** | **9** | **17** | **10,405** |
| Medication | 3 | 5 | 4,264 | 3 | 8 | 6,798 | 3 | 8 | 6,336 | 3 | 8 | 6,535 |
| Procedure | 2 | 6 | 4,508 | 3 | 5 | 5,894 | 3 | 4 | 2,754 | 3 | 2 | 2,705 |
| Consultation | 3 | 6 | 1,025 | 3 | 11 | 3,365 | 3 | 8 | 1,803 | 3 | 8 | 1,165 |
| Consumables | 1 | 1 | 630 | 1 | 2 | 683 |  |  |  |  |  |  |
| Services | 1 | 1 | 5,797 | 1 | 1 | 5,797 |  |  |  |  |  |  |
| Others |  |  |  | 1 | 1 | 0 | 1 | 1 | 0 |  |  |  |
| **T2DM+Chronic renal failure** | **41** | **15** | **8,623** | **40** | **24** | **23,150** | **43** | **19** | **13,712** | **47** | **19** | **11,234** |
| Medication | 12 | 4 | 5,387 | 12 | 7 | 7,927 | 13 | 6 | 6,564 | 13 | 5 | 4,330 |
| Procedure | 12 | 3 | 2,064 | 13 | 6 | 8,328 | 13 | 5 | 5,557 | 13 | 3 | 3,004 |
| Consultation | 12 | 5 | 741 | 12 | 7 | 1,907 | 13 | 6 | 1,287 | 13 | 5 | 950 |
| Consumables | 3 | 1 | 101 | 1 | 2 | 318 | 4 | 2 | 304 | 5 | 3 | 390 |
| Services | 2 | 2 | 330 | 2 | 2 | 4,671 |  |  |  | 2 | 2 | 2,560 |
| Others |  |  |  |  |  |  |  |  |  | 1 | 1 | 0 |
| **T2DM+Coronary Artery Disease** | **179** | **17** | **6,410** | **180** | **24** | **12,413** | **174** | **24** | **8,838** | **182** | **18** | **9,035** |
| Medication | 58 | 5 | 2,762 | 58 | 7 | 6,469 | 55 | 7 | 4,627 | 59 | 5 | 3,383 |
| Procedure | 48 | 3 | 1,622 | 52 | 4 | 3,574 | 51 | 4 | 2,295 | 52 | 3 | 3,233 |
| Consultation | 59 | 5 | 377 | 58 | 7 | 930 | 55 | 7 | 586 | 59 | 5 | 398 |
| Consumables | 6 | 2 | 240 | 5 | 3 | 566 | 4 | 2 | 228 | 5 | 2 | 269 |
| Services | 2 | 1 | 1,380 | 5 | 2 | 875 | 8 | 2 | 1,103 | 4 | 2 | 1,751 |
| Others | 6 | 1 | 29 | 2 | 2 | 0 | 1 | 1 | 0 | 3 | 1 | 0 |
| **T2DM+Dysrhythmia** | **22** | **9** | **2,616** | **25** | **18** | **6,918** | **23** | **21** | **8,027** | **25** | **11** | **3,896** |
| Medication | 6 | 3 | 1,212 | 8 | 5 | 2,566 | 7 | 6 | 3,856 | 8 | 3 | 2,483 |
| Procedure | 7 | 2 | 1,159 | 7 | 4 | 3,361 | 7 | 4 | 3,274 | 8 | 2 | 911 |
| Consultation | 7 | 2 | 189 | 8 | 6 | 968 | 7 | 7 | 709 | 7 | 4 | 290 |
| Consumables |  |  |  | 1 | 1 | 21 | 2 | 4 | 188 | 2 | 3 | 212 |
| Services |  |  |  | 1 | 1 | 2 |  |  |  |  |  |  |
| Others | 2 | 2 | 56 |  |  |  |  |  |  |  |  |  |
| **T2DM+Heart Failure** | **33** | **15** | **8,794** | **36** | **28** | **68,101** | **34** | **19** | **25,204** | **33** | **17** | **6,786** |
| Medication | 10 | 4 | 5,064 | 9 | 7 | 16,921 | 10 | 5 | 7,282 | 10 | 6 | 3,850 |
| Procedure | 10 | 2 | 2,031 | 9 | 4 | 14,783 | 6 | 4 | 6,719 | 8 | 2 | 1,564 |
| Consultation | 10 | 4 | 832 | 9 | 8 | 5,405 | 10 | 5 | 1,428 | 10 | 6 | 1,022 |
| Consumables | 3 | 4 | 867 | 6 | 3 | 3,073 | 6 | 3 | 632 | 5 | 3 | 350 |
| Services |  |  |  | 2 | 3 | 27,919 | 1 | 1 | 9,143 |  |  |  |
| Others |  |  |  | 1 | 3 | 0 | 1 | 1 | 0 |  |  |  |
| **T2DM+Myocardial infarction** | **3** | **18** | **3,324** | **3** | **15** | **1,795** | **3** | **26** | **2,590** | **3** | **7** | **929** |
| Medication | 1 | 7 | 2,144 | 1 | 7 | 1,060 | 1 | 10 | 1,680 | 1 | 3 | 739 |
| Procedure | 1 | 5 | 770 | 1 | 4 | 585 | 1 | 7 | 560 | 1 | 2 | 110 |
| Consultation | 1 | 6 | 410 | 1 | 4 | 150 | 1 | 9 | 350 | 1 | 2 | 80 |
| Consumables |  |  |  |  |  |  |  |  |  |  |  |  |
| Services |  |  |  |  |  |  |  |  |  |  |  |  |
| Others |  |  |  |  |  |  |  |  |  |  |  |  |
| **T2DM+Other Cardiovascular Disease** | **14** | **23** | **11,991** | **13** | **26** | **19,125** | **14** | **19** | **11,291** | **15** | **13** | **4,762** |
| Medication | 4 | 7 | 4,159 | 4 | 7 | 8,118 | 4 | 6 | 5,949 | 4 | 3 | 1,973 |
| Procedure | 4 | 7 | 6,508 | 4 | 6 | 8,721 | 4 | 5 | 4,127 | 4 | 4 | 1,984 |
| Consultation | 4 | 7 | 1,101 | 4 | 8 | 1,642 | 4 | 5 | 853 | 4 | 3 | 626 |
| Consumables | 2 | 3 | 223 | 1 | 5 | 645 | 2 | 3 | 361 | 2 | 2 | 179 |
| Services |  |  |  |  |  |  |  |  |  |  |  |  |
| Others |  |  |  |  |  |  |  |  |  | 1 | 1 | 0 |
| **T2DM+Periphery vascular disease** | **8** | **37** | **15,222** | **8** | **89** | **68,954** | **7** | **9** | **10,818** | **9** | **30** | **27,762** |
| Medication | 2 | 3 | 9,649 | 2 | 7 | 15,001 | 2 | 2 | 6,143 | 2 | 7 | 16,305 |
| Procedure | 2 | 3 | 2,581 | 2 | 7 | 10,475 | 2 | 3 | 3,612 | 2 | 5 | 7,564 |
| Consultation | 2 | 4 | 870 | 2 | 8 | 1,901 | 2 | 3 | 559 | 2 | 7 | 1,313 |
| Consumables | 1 | 4 | 1,200 | 1 | 4 | 1,086 | 1 | 2 | 504 | 2 | 5 | 401 |
| Services | 1 | 24 | 922 | 1 | 64 | 40,491 |  |  |  | 1 | 7 | 2,180 |
| Others |  |  |  |  |  |  |  |  |  |  |  |  |
| **T2DM+Stroke or TIA** | **103** | **15** | **10,304** | **103** | **23** | **18,638** | **103** | **19** | **11,034** | **104** | **16** | **8,367** |
| Medication | 30 | 4 | 3,940 | 32 | 6 | 7,492 | 32 | 5 | 4,816 | 31 | 4 | 4,017 |
| Procedure | 30 | 3 | 3,052 | 32 | 4 | 6,837 | 29 | 3 | 3,976 | 29 | 3 | 2,573 |
| Consultation | 31 | 4 | 796 | 31 | 7 | 2,115 | 32 | 5 | 987 | 32 | 4 | 818 |
| Consumables | 7 | 2 | 341 | 6 | 3 | 880 | 6 | 4 | 497 | 8 | 2 | 355 |
| Services | 2 | 1 | 2,105 | 2 | 3 | 1,314 | 2 | 1 | 708 | 2 | 1 | 560 |
| Others | 3 | 1 | 71 |  |  |  | 2 | 2 | 50 | 2 | 3 | 45 |
| **T2DM WITH MULTIPLE CVD** | | | | | | | | | | | | |
| **Coronary Arterial Revascularization+T2DM+Coronary Artery Disease** | **12** | **18** | **7,419** | **13** | **21** | **15,537** | **13** | **20** | **13,846** | **16** | **16** | **64,270** |
| Medication | 4 | 5 | 3,732 | 4 | 8 | 10,875 | 4 | 7 | 7,820 | 4 | 5 | 5,893 |
| Procedure | 3 | 4 | 2,258 | 4 | 3 | 2,981 | 4 | 4 | 4,420 | 3 | 3 | 54,865 |
| Consultation | 4 | 5 | 679 | 4 | 8 | 1,081 | 4 | 7 | 1,289 | 4 | 6 | 1,004 |
| Consumables | 1 | 4 | 750 | 1 | 3 | 600 | 1 | 2 | 318 | 3 | 2 | 1,917 |
| Services |  |  |  |  |  |  |  |  |  | 2 | 1 | 591 |
| Others |  |  |  |  |  |  |  |  |  |  |  |  |
| **Coronary Arterial Revascularization+T2DM+Coronary Artery Disease+Angina** | **9** | **10** | **8,426** | **10** | **21** | **20,994** | **9** | **18** | **17,099** | **13** | **26** | **26,377** |
| Medication | 3 | 3 | 5,315 | 3 | 8 | 15,651 | 3 | 10 | 13,851 | 3 | 9 | 8,583 |
| Procedure | 3 | 2 | 2,702 | 3 | 4 | 4,223 | 3 | 2 | 2,533 | 3 | 4 | 14,346 |
| Consultation | 3 | 4 | 410 | 3 | 8 | 952 | 3 | 6 | 715 | 3 | 9 | 1,213 |
| Consumables |  |  |  | 1 | 1 | 168 |  |  |  | 1 | 1 | 220 |
| Services |  |  |  |  |  |  |  |  |  | 1 | 2 | 1,685 |
| Others |  |  |  |  |  |  |  |  |  | 2 | 2 | 330 |
| **Coronary Arterial Revascularization+T2DM+Heart Failure+Coronary Artery Disease** | **7** | **12** | **4,042** | **7** | **24** | **21,027** | **7** | **20** | **8,565** | **7** | **21** | **13,211** |
| Medication | 2 | 4 | 1,633 | 2 | 5 | 12,961 | 2 | 7 | 6,745 | 2 | 9 | 5,379 |
| Procedure | 2 | 4 | 1,953 | 1 | 7 | 4,285 | 2 | 3 | 568 | 1 | 3 | 3,520 |
| Consultation | 2 | 4 | 405 | 2 | 6 | 1,869 | 2 | 8 | 780 | 2 | 7 | 663 |
| Consumables | 1 | 1 | 51 | 1 | 5 | 1,448 | 1 | 3 | 473 | 1 | 2 | 600 |
| Services |  |  |  | 1 | 1 | 465 |  |  |  | 1 | 1 | 3,050 |
| Others |  |  |  |  |  |  |  |  |  |  |  |  |
| **T2DM+Chronic renal failure+Angina** | **4** | **16** | **6,775** | **5** | **28** | **27,051** | **7** | **26** | **27,091** | **7** | **22** | **19,435** |
| Medication | 1 | 5 | 2,288 | 2 | 8 | 10,161 | 2 | 8 | 14,646 | 2 | 7 | 8,106 |
| Procedure | 1 | 6 | 3,931 | 1 | 11 | 13,669 | 2 | 7 | 9,057 | 2 | 6 | 8,762 |
| Consultation | 2 | 5 | 556 | 2 | 10 | 3,221 | 2 | 10 | 3,305 | 2 | 8 | 2,468 |
| Consumables |  |  |  |  |  |  | 1 | 1 | 84 |  |  |  |
| Services |  |  |  |  |  |  |  |  |  |  |  |  |
| Others |  |  |  |  |  |  |  |  |  | 1 | 1 | 100 |
| **T2DM+Coronary Artery Disease+Angina** | **35** | **19** | **10,286** | **41** | **23** | **46,352** | **34** | **17** | **10,253** | **36** | **16** | **10,514** |
| Medication | 11 | 5 | 4,688 | 11 | 6 | 13,514 | 11 | 5 | 5,325 | 11 | 4 | 4,778 |
| Procedure | 10 | 4 | 2,144 | 11 | 4 | 14,897 | 9 | 4 | 3,507 | 11 | 3 | 3,295 |
| Consultation | 11 | 5 | 672 | 11 | 7 | 2,116 | 11 | 5 | 807 | 10 | 5 | 930 |
| Consumables | 1 | 2 | 360 | 2 | 3 | 12,725 | 1 | 1 | 84 | 2 | 3 | 222 |
| Services | 1 | 1 | 2,396 | 5 | 1 | 3,091 | 2 | 2 | 530 | 2 | 2 | 1,288 |
| Others | 1 | 2 | 25 | 1 | 1 | 10 |  |  |  |  |  |  |
| **T2DM+Coronary Artery Disease+Atrial fibrillation** | **8** | **25** | **7,030** | **9** | **33** | **16,801** | **6** | **34** | **18,739** | **5** | **13** | **8,561** |
| Medication | 2 | 6 | 4,531 | 2 | 9 | 9,097 | 2 | 11 | 9,343 | 2 | 4 | 3,543 |
| Procedure | 2 | 6 | 1,444 | 2 | 10 | 3,864 | 2 | 10 | 7,248 | 1 | 5 | 4,720 |
| Consultation | 2 | 6 | 580 | 2 | 12 | 1,290 | 2 | 13 | 2,148 | 2 | 4 | 298 |
| Consumables | 1 | 6 | 475 |  |  |  |  |  |  |  |  |  |
| Services |  |  |  | 2 | 1 | 2,550 |  |  |  |  |  |  |
| Others | 1 | 1 | 0 | 1 | 1 | 0 |  |  |  |  |  |  |
| **T2DM+Coronary Artery Disease+Chronic renal failure** | **16** | **11** | **22,536** | **17** | **17** | **30,573** | **14** | **13** | **11,966** | **18** | **15** | **13,020** |
| Medication | 5 | 3 | 6,387 | 5 | 4 | 12,636 | 4 | 4 | 7,221 | 5 | 5 | 9,689 |
| Procedure | 4 | 3 | 5,243 | 4 | 3 | 5,748 | 3 | 3 | 3,263 | 5 | 3 | 2,349 |
| Consultation | 5 | 3 | 942 | 5 | 4 | 1,770 | 4 | 4 | 1,137 | 5 | 4 | 812 |
| Consumables | 1 | 1 | 225 | 1 | 3 | 530 | 2 | 1 | 45 | 2 | 2 | 170 |
| Services | 1 | 1 | 9,740 | 1 | 1 | 9,740 | 1 | 1 | 300 |  |  |  |
| Others |  |  |  | 1 | 1 | 150 |  |  |  | 1 | 1 | 0 |
| **T2DM+Heart Failure+Angina** | **7** | **21** | **12,955** | **5** | **19** | **17,044** | **7** | **36** | **14,374** | **7** | **19** | **11,680** |
| Medication | 2 | 7 | 4,427 | 2 | 7 | 5,080 | 2 | 10 | 7,085 | 2 | 6 | 5,247 |
| Procedure | 1 | 4 | 6,190 | 1 | 3 | 10,320 | 2 | 8 | 4,352 | 2 | 4 | 5,291 |
| Consultation | 2 | 8 | 1,220 | 2 | 10 | 1,644 | 2 | 12 | 2,133 | 2 | 6 | 805 |
| Consumables | 1 | 2 | 318 |  |  |  | 1 | 7 | 804 | 1 | 4 | 336 |
| Services | 1 | 1 | 800 |  |  |  |  |  |  |  |  |  |
| Others |  |  |  |  |  |  |  |  |  |  |  |  |
| **T2DM+Heart Failure+Coronary Artery Disease** | **9** | **17** | **5,349** | **10** | **20** | **15,353** | **6** | **20** | **11,101** | **11** | **14** | **5,463** |
| Medication | 3 | 6 | 2,578 | 3 | 7 | 8,236 | 2 | 8 | 7,464 | 3 | 4 | 2,877 |
| Procedure | 2 | 2 | 880 | 2 | 3 | 3,194 | 2 | 5 | 2,810 | 3 | 2 | 1,723 |
| Consultation | 3 | 5 | 626 | 3 | 7 | 1,435 | 2 | 7 | 828 | 3 | 5 | 573 |
| Consumables |  |  |  |  |  |  |  |  |  | 1 | 1 | 90 |
| Services | 1 | 4 | 1,265 | 1 | 2 | 2,405 |  |  |  | 1 | 1 | 200 |
| Others |  |  |  | 1 | 1 | 83 |  |  |  |  |  |  |
| **T2DM+Heart Failure+Coronary Artery Disease+Atrial fibrillation** | **6** | **6** | **2,774** | **6** | **14** | **12,556** | **10** | **14** | **16,742** | **4** | **2** | **129** |
| Medication | 2 | 2 | 886 | 2 | 5 | 5,261 | 2 | 5 | 4,037 | 2 | 1 | 79 |
| Procedure | 2 | 2 | 1,381 | 2 | 3 | 5,047 | 2 | 3 | 6,318 |  |  |  |
| Consultation | 2 | 2 | 508 | 2 | 6 | 2,249 | 2 | 3 | 1,313 | 2 | 1 | 50 |
| Consumables |  |  |  |  |  |  | 2 | 2 | 785 |  |  |  |
| Services |  |  |  |  |  |  | 1 | 1 | 4,289 |  |  |  |
| Others |  |  |  |  |  |  | 1 | 1 | 0 |  |  |  |
| **T2DM+Myocardial infarction+Coronary Artery Disease** | **18** | **10** | **4,188** | **18** | **28** | **52,578** | **17** | **16** | **6,453** | **18** | **13** | **6,595** |
| Medication | 5 | 2 | 1,661 | 5 | 9 | 8,554 | 5 | 6 | 3,721 | 5 | 4 | 2,422 |
| Procedure | 4 | 2 | 1,483 | 4 | 7 | 34,800 | 5 | 2 | 1,649 | 4 | 2 | 2,026 |
| Consultation | 5 | 3 | 219 | 5 | 10 | 1,999 | 5 | 6 | 636 | 5 | 4 | 458 |
| Consumables | 2 | 2 | 17 |  |  |  |  |  |  | 1 | 1 | 5 |
| Services | 2 | 1 | 808 | 4 | 2 | 7,225 | 2 | 2 | 448 | 2 | 1 | 1,294 |
| Others |  |  |  |  |  |  |  |  |  | 1 | 1 | 390 |
| **T2DM+Stroke or TIA+Angina** | **8** | **7** | **2,636** | **7** | **16** | **10,103** | **10** | **15** | **13,519** | **7** | **13** | **5,724** |
| Medication | 3 | 2 | 952 | 2 | 6 | 3,892 | 3 | 5 | 2,315 | 2 | 7 | 4,078 |
| Procedure | 2 | 2 | 1,531 | 2 | 3 | 4,870 | 3 | 3 | 5,081 | 2 | 2 | 1,145 |
| Consultation | 3 | 3 | 153 | 2 | 6 | 1,247 | 3 | 6 | 448 | 3 | 5 | 501 |
| Consumables |  |  |  | 1 | 1 | 94 |  |  |  |  |  |  |
| Services |  |  |  |  |  |  | 1 | 1 | 5,675 |  |  |  |
| Others |  |  |  |  |  |  |  |  |  |  |  |  |
| **T2DM+Stroke or TIA+Chronic renal failure** | **17** | **17** | **8,536** | **16** | **43** | **35,179** | **19** | **22** | **11,953** | **17** | **25** | **20,818** |
| Medication | 5 | 4 | 3,845 | 4 | 8 | 12,843 | 5 | 6 | 4,689 | 4 | 7 | 7,166 |
| Procedure | 5 | 5 | 3,629 | 4 | 7 | 12,273 | 5 | 3 | 4,163 | 5 | 5 | 7,706 |
| Consultation | 5 | 4 | 789 | 4 | 10 | 3,852 | 5 | 7 | 2,063 | 5 | 7 | 1,766 |
| Consumables | 2 | 4 | 273 | 2 | 6 | 1,087 | 2 | 2 | 332 | 1 | 1 | 84 |
| Services |  |  |  | 1 | 11 | 4,682 | 1 | 2 | 432 | 1 | 3 | 4,095 |
| Others |  |  |  | 1 | 2 | 441 | 1 | 2 | 275 | 1 | 2 | 0 |
| **T2DM+Stroke or TIA+Coronary Artery Disease** | **12** | **19** | **9,578** | **13** | **22** | **11,154** | **13** | **22** | **11,634** | **9** | **8** | **4,715** |
| Medication | 4 | 8 | 4,642 | 4 | 8 | 5,137 | 4 | 8 | 6,196 | 2 | 5 | 2,981 |
| Procedure | 3 | 3 | 3,537 | 3 | 4 | 3,213 | 2 | 5 | 2,367 | 3 | 1 | 1,351 |
| Consultation | 4 | 7 | 1,369 | 4 | 9 | 2,392 | 4 | 8 | 1,065 | 4 | 3 | 383 |
| Consumables |  |  |  |  |  |  |  |  |  |  |  |  |
| Services | 1 | 1 | 30 | 2 | 2 | 412 | 2 | 1 | 1,996 |  |  |  |
| Others |  |  |  |  |  |  | 1 | 1 | 10 |  |  |  |
| **T2DM+Stroke or TIA+Coronary Artery Disease+Atrial fibrillation** | **7** | **13** | **8,282** | **7** | **15** | **15,448** | **8** | **41** | **31,994** | **6** | **22** | **14,639** |
| Medication | 2 | 4 | 3,448 | 2 | 6 | 7,339 | 2 | 13 | 11,080 | 2 | 8 | 7,601 |
| Procedure | 2 | 4 | 4,137 | 2 | 5 | 6,586 | 2 | 11 | 10,928 | 2 | 7 | 5,725 |
| Consultation | 2 | 3 | 504 | 2 | 4 | 1,426 | 2 | 10 | 1,636 | 2 | 8 | 1,313 |
| Consumables | 1 | 2 | 193 | 1 | 1 | 98 | 1 | 5 | 542 |  |  |  |
| Services |  |  |  |  |  |  | 1 | 3 | 7,808 |  |  |  |
| Others |  |  |  |  |  |  |  |  |  |  |  |  |

Abbreviations: CVD=Cardiovascular disease, HCRU=Healthcare cost utilization, N=Number of patients, T2DM=Type 2 diabetes mellitus, TIA=Transient ischemic attack
