# Supplementary material for: Cost of cardiovascular diseases and renal complications in people with type 2 diabetes mellitus in the Kingdom of Saudi Arabia: A retrospective analysis of claims database
Source: PLoS One. 2022 Oct 20;17(10):e0273836. doi: 10.1371/journal.pone.0273836 (PMC9584438; doi:10.1371/journal.pone.0273836)
Supplement: Attachment — Submitted filename: Response to Reviewers.docx [file pone.0273836.s024.docx]

**Rebuttal letter**

To	Date: 3/08/2022

The Editor-In-Chief

PLOS ONE

Subject: Responses to peer reviewers and editors’ comments for original research (Manuscript number: PONE-D-22-02281)

Title: Cost of cardiovascular diseases and renal complications in people with type 2 diabetes mellitus in the Kingdom of Saudi Arabia: A retrospective analysis of claims database.

Dear Managing Editor,

We would like to thank reviewers for their valuable time and constructive comments in our manuscript. We appreciate that the inputs they have given will help to improve the manuscript. Following are the point-by-point responses to the reviewer’s comments.

| **S.No.** | **Comments** | **Responses** |
| --- | --- | --- |
| **REVIEWER 1 COMMENTS** | | |
| 1 | The abstract is very clear. There are too many keywords in the abstract, I suggest you review this point. You can  leave only 5 keywords. | Thank you for the comments.  We have revised and retained only 5 keywords  Type 2 diabetes mellitus; cardiovascular disease; claims database; healthcare resource utilization; chronic renal failure |
| 2 | Introduction is very clear with the use of recent bibliographic references.  Results and Methods are clear, but please review Table 4 as it seems that the table is not fully appearing. | Thank you for the comment  We have reviewed Table 4 and taken care of formatting for complete visibility of the table |
| 3 | Please review the conclusion part of the article. | Thank you for the comment.  We have revised the conclusion part as below.  The current analyses shed light on the substantial economic burden in patients with T2DM with comorbid CVD and renal diseases. The study observed a greater frequency of hospitalization in patients with T2DM with associated CVD and renal comorbidities. The findings also suggest a gradual increase in average healthcare resource cost over the 1-year post-index period compared with the 1-year pre-index period.  Evidence suggests that glucagon-like peptide-1 (GLP-1) agonists are associated with reduced risk of CVS and renal events and sodium-glucose-cotransporter-2 (SGLT2) have improved renal outcomes in patients with T2DM. Therefore, early initiation of these T2DM therapies would reduce long-term cardiovascular and renal outcomes and reduce the associated costs in patients with T2DM. |
|  | **REVIEWER 2 COMMENTS** |  |
|  | The authors submitted a research article in which they assessed the healthcare resource utilization and costs related to cardiovascular and renal complications among patients with T2DM. They designed non-interventional, longitudinal, retrospective, cohort study collected secondary data from three insurance claims databases across Kingdom of Saudi Arabia. The authors found that the healthcare resource utilization and cost gradually increased over the 3-year post-index period compared with the 1-year pre-index period. Finally, they suggested that better management of T2DM to limit the associated  cost burden is need of the hour. The strength of the study is a large sample size, the weakness is a retropsective design. Albeit the findings seem to be impressive, I would like to out forward several issues to discuss. |  |
| 1. | Did authors evaluate a bias of the study related to duration of the T2DM complication treating? Whether both healthcare resource utilization and cost are regarded to be attributed to severity of the disease? | Thank you for the comment  In the current study, HCRU and cost was evaluated in T2DM patients with one or multiple comorbidities (CVD/ renal disease). Claims and costs pertaining to visit and activity types were evaluated for pre-index and post-index period across all three databases. An increase in costs incurred based various visit and activity types was noted during the post-index period as compared to pre-index period.  Generally, T2DM with CVD or renal complication will be associated with increased cost with severity and duration of disease. However, in the current study, we could not assess the effect of severity of disease on healthcare cost, as the patient number reduced with the follow-up period. We were not able to rule out any bias related to duration and severity of the T2DM complication. |
| 2. | Was sensitivity analysis performed? Please, give an extensive explanation about it. | Thank you for the comment  Sensitivity analysis was not performed in the current study. The study included data from 3 insurance claims databases in KSA covering both the local Saudi population and expatriate population working in the private sector. Each database included longitudinal, anonymized, patient level data, representing a geographically varied range of administrative claims information and capturing data from hospitals, pharmacies, clinics, polyclinics, laboratories, and other providers.  In the current study, descriptive analysis was used to analyse the patient demographics (age, gender and nationality) and HCRU and costs. As the patient numbers in the 1year post-index period are high and we have used the average cost, which could be representative of true cost. |
| 3. | The conclusive part should compose of a clear and practically useful recommendation. | Thank you for the comment  We have revised the conclusion part as below.  The current analyses shed light on the substantial economic burden in patients with T2DM with comorbid CVD and renal diseases. The study observed a greater frequency of hospitalization in patients with T2DM with associated CVD and renal comorbidities. The findings also suggest a gradual increase in average healthcare resource cost during 1-year post-index period compared with the 1-year pre-index period.  Evidence suggests that glucagon-like peptide-1 (GLP-1) agonists are associated with reduced risk of CVS and renal events and sodium-glucose-cotransporter-2 (SGLT2) have improved renal outcomes in patients with T2DM. Therefore, early initiation of these T2DM therapies would reduce long-term cardiovascular and renal outcomes and the associated costs in patients with T2DM. |
|  | Editors Comments |  |
| 1 | Please ensure that your manuscript meets PLOS ONE's style requirements, including those for file naming. The PLOS ONE style templates can be found at  <https://journals.plos.org/plosone/s/file?id=wjVg/PLOSOne_formatting_sample_main_body.pdf>  and  <https://journals.plos.org/plosone/s/file?id=ba62/PLOSOne_formatting_sample_title_authors_affiliations.pdf> | Thank you for the comments  Manuscript content has been amended appropriately to meet the PLOS ONE style template requirements pertaining to “Manuscript body formatting”, “Author list and affiliations formatting”, “Supporting Information formatting” |
| 2 | We note that the grant information you provided in the ‘Funding Information’ and ‘Financial Disclosure’ sections do not match.  When you resubmit, please ensure that you provide the correct grant numbers for the awards you received for your study in the ‘Funding Information’ section. | Thank you for the comment  During resubmission, appropriate changes to funding information will be made |
| 3 | Thank you for stating the following in the Acknowledgments/Funding Section of your manuscript:  The study was funded by NovoNordisk Saudi Arabia  We note that you have provided funding information that is not currently declared in your Funding Statement. However, funding information should not appear in the Acknowledgments section or other areas of your manuscript. We will only publish funding information present in the Funding Statement section of the online submission form.  Please remove any funding-related text from the manuscript and let us know how you would like to update your Funding Statement. Currently, your Funding Statement reads as follows:  The author(s) received no specific funding for this work.  Please include your amended statements within your cover letter; we will change the online submission form on your behalf. | Thank you for the comment  As suggested, we have removed the funding related information from the manuscript.  We have updated the Funding statement as stated below shall include the below amended statement within the cover letter  The study was funded by Novo Nordisk, KSA and had role in the study design, study methodology and decision to publish. |
| 4 | Please review your reference list to ensure that it is complete and correct. If you have cited papers that have been retracted, please include the rationale for doing so in the manuscript text, or remove these references and replace them with relevant current references. Any changes to the reference list should be mentioned in the rebuttal letter that accompanies your revised manuscript. If you need to cite a retracted article, indicate the article’s retracted status in the References list and also include a citation and full reference for the retraction notice. | Thank you for the comments  We have reviewed the reference list and the list is complete and correct. None of the cited references have been retracted. We have not made any changes to the existing reference list. |
